# Genome editing reveals that pSCL4 is required for chromosome linearity in *Streptomyces clavuligerus*

**DOI:** 10.1099/mgen.0.000669

**Published:** 2021-11-08

**Authors:** Juan Pablo Gomez-Escribano, Lis Algora Gallardo, Kenan A. J. Bozhüyük, Steven G. Kendrew, Benjamin D. Huckle, Nicola A. Crowhurst, Mervyn J. Bibb, Andrew J. Collis, Jason Micklefield, Paul R. Herron, Barrie Wilkinson

**Affiliations:** ^1^​ Department of Molecular Microbiology, John Innes Centre, Norwich Research Park, Norwich NR4 7UH, UK; ^2^​ Strathclyde Institute of Pharmacy and Biomedical Sciences, University of Strathclyde, 161 Cathedral Street, Glasgow G4 0RE, UK; ^3^​ Biotechnology and Environmental Shared Service, GlaxoSmithKline, Southdown View Way, Worthing BN14 8QH, UK; ^4^​ Engineered Biodesign Limited, Cambridge CB1 3SN, UK; ^5^​ Department of Chemistry, Manchester Institute for Biotechnology, University of Manchester, 131 Princess Street, Manchester M1 7DN, UK; ^†^​ Present address: Department of Bioresources for Bioeconomy and Health Research, Leibniz Institute, DSMZ-German Collection of Microorganisms and Cell Cultures, Inhoffenstraße 7B, 38124 Braunschweig, Germany; ^‡^​ Present address: Molecular Biotechnology, Department of Biosciences, Goethe University Frankfurt, 60438 Frankfurt am Main, Germany

**Keywords:** actinomycetes genomics, Bionano optical mapping, CRISPR-Cas9, next-generation sequencing, PacBio, plasmid curing

## Abstract

*
Streptomyces clavuligerus* is an industrially important actinomycete whose genetic manipulation is limited by low transformation and conjugation efficiencies, low levels of recombination of introduced DNA, and difficulty in obtaining consistent sporulation. We describe the construction and application of versatile vectors for Cas9-mediated genome editing of this strain. To design spacer sequences with confidence, we derived a highly accurate genome assembly for an isolate of the type strain (ATCC 27064). This yielded a chromosome assembly (6.75 Mb) plus assemblies for pSCL4 (1795 kb) and pSCL2 (149 kb). The strain also carries pSCL1 (12 kb), but its small size resulted in only partial sequence coverage. The previously described pSCL3 (444 kb) is not present in this isolate. Using our Cas9 vectors, we cured pSCL4 with high efficiency by targeting the plasmid’s *parB* gene. Five of the resulting pSCL4-cured isolates were characterized and all showed impaired sporulation. Shotgun genome sequencing of each of these derivatives revealed large deletions at the ends of the chromosomes in all of them, and for two clones sufficient sequence data was obtained to show that the chromosome had circularized. Taken together, these data indicate that pSCL4 is essential for the structural stability of the linear chromosome.

## Data Summary

DNA sequence data generated during this study have been deposited at the National Center for Biotechnology Information with accession numbers PRJNA579405 (BioProject), CP045847, CP045848, CP045849 and CP045850 for the main assembly, and BioProjects PRJNA587782 and PRJNA587886 for the Illumina data.

**Impact Statement**

*
Streptomyces* species and allied actinomycetes provide the origin of over half of the antibiotics in clinical use. *Streptomyces clavuligerus* is the industrial producer of the β-lactamase inhibitor clavulanic acid, which is formulated with the β-lactam antibiotic amoxicillin to yield the World Health Organization's essential medicine Augmentin. In this work, we report the most comprehensive assembly of the *Streptomyces clavuligerus* genome to date, in addition to new vectors that allow effective CRISPR-Cas9 gene editing in this strain. This is significant as *S. clavuligerus* is poorly recombinogenic and the availability of these tools will facilitate future studies with this industrially important organism.

## Introduction

*Streptomyces clavuligerus* is the industrial producer of the commercially important β-lactamase inhibitor clavulanic acid [1]. Clavulanate potassium (the potassium salt of clavulanic acid) formulated along with the semi-synthetic β-lactam antibiotic amoxicillin forms the antibacterial treatment Augmentin (also sold under the generic name co-amoxiclav), which is on the World Health Organization’s list of essential medicines [2]. *Streptomyces clavuligerus* is also one of the few actinomycetes known to produce β-lactam antibiotics, the penicillin-related cephamycin C and its biosynthetic intermediates isopenicillin N and penicillin N [3, 4]. *Streptomyces clavuligerus* additionally produces a plethora of metabolites with antifungal and antibacterial activity that are biosynthetically related to clavulanic acid and known as 5*S*-clavams, of which alanylclavam is the most abundantly produced [5]. The biosynthetic capacity of this micro-organism does not end there, it also produces the pyrrothin antibiotics holomycin and holothin [6], the nucleoside antibiotic tunicamycin [7, 8] and the flavonoid naringenin amongst others [9].

There has been significant effort put into delineating the biosynthesis of these molecules, and into understanding the molecular genetics and microbial physiology of *Streptomyces clavuligerus* [5, 7, 10–12]. The genetics of *Streptomyces clavuligerus* has been extensively studied for nearly 40 years and with up to four linear plasmids, two of them among the largest plasmids known, the genome of this species is one of the most complex among bacteria [11, 13–17]. Genetic manipulation of *Streptomyces clavuligerus* can be difficult using standard *Streptomyces* protocols, which often require considerable modification for efficiency [9, 10]. Work in this strain has been further hampered by low transformation and conjugation efficiencies, and low levels of recombination compared to, for example, the model organism *Streptomyces coelicolor *, meaning relatively long segments of homologous DNA and multiple rounds of sporulation are required to obtain double-crossover recombinants.

A proven strategy to efficiently select for double-crossover events where regions of sequence homology exist is to cause double-strand breaks in the DNA. This has been successfully implemented in *Streptomyces* by using the yeast meganuclease SceI [18]. More recently, the CRISPR-Cas9 system from *Streptococcus pyogenes* [19] has been adapted for genetic manipulation in *Streptomyces* [20–23], and the *Streptococcus pyogenes* Cas9 can recognize any target sequence followed by the triplet NGG [19]. *Streptomyces* and other actinomycete genomes typically possess a G+C content of approximately 70 mol% and, therefore, a high frequency of NGG sequence motifs. Consequently, for these organisms, the availability of a high-quality genome sequence is an important criterion for the precise identification of unique target sites and reliable CRISPR-Cas9 editing.

Two good-quality genome assemblies of the *Streptomyces clavuligerus* type strain were available when this work was started. These were obtained by the University of Groningen [13] (accession no. GCA_000163875.1, hereinafter referred to as the ‘Groningen assembly’, and derived from the *Streptomyces clavuligerus* ATCC 27064 deposit) [5], and by the Korea Research Institute of Bioscience and Biotechnology [14] (accession no. GCA_000148465, hereinafter referred to as the ‘KRIBB assembly’, and derived from the *Streptomyces clavuligerus* NRRL 3585 deposit) [6]. In principle, these two isolates of the type strain should be the same (see Table 1). Another assembly, but of lower quality, had been released by the Broad Institute (accession no. GCA_000154925.1, hereinafter referred to as the ‘Broad assembly’, and derived from the *Streptomyces clavuligerus* ATCC 27064 deposit). Unfortunately, all these assemblies are fragmented to different degrees and likely to lack significant sequence information. Two further high-quality assemblies for *Streptomyces clavuligerus* exist (accession numbers GCA_001693675.1 [8] and GCA_003454755.1), but these are derived from industrial isolates where the origin and history of the strains is unknown. The first of these non-type industrial strain assemblies has recently been adopted as the representative genome for *Streptomyces clavuligerus* by the National Center for Biotechnology Information (NCBI) (https://www.ncbi.nlm.nih.gov/genome/?term=txid1901). These data are summarized in Table 2 (at the time of writing this paper, a new high-quality assembly of a type isolate was made public [17], whose relationship with ours and the previously published ones will be discussed).

**Table 1.** *
Streptomyces clavuligerus* strains used and constructed during this work

Strain designation	Strain description	Source	Reference
León isolate	Type strain clone source of the genomic DNA sequenced in this study	Laboratory of Paloma Liras, University of León, Spain	[64]
ATCC 27064	Type strain, originally deposited by Higgens and Kastner	American Type Culture Collection (ATCC)	[4]; https://www.atcc.org/products/27064
NRRL 3585	Type strain, originally deposited by Higgens and Kastner	Northern Regional Research Laboratory (NRRL)	[4]
DSM 738	Type strain, transferred to DSMZ from ATCC	German Collection of Microorganisms and Cell Cultures GmbH (DSMZ)	www.dsmz.de/collection/catalogue/details/culture/DSM-738
NCIMB 12785	Type strain, transferred to NCIMB from the Japanese Collection of Microorganisms (JCM)	National Collection of Industrial Food and Marine Bacteria (NCIMB)	https://store.ncimb.com/page/Strains%20record%20name%20display/56655
NCIMB 14335	Type strain, transferred to NCIMB from DSMZ	National Collection of Industrial Food and Marine Bacteria (NCIMB)	https://store.ncimb.com/page/Strains%20record%20name%20display/59992
BW0216	León isolate cured of pSCL4 Clear evidence of loss of terminal sequence and of chromosome having circularized	This work	This work
BW0217	León isolate cured of pSCL4 Clear evidence of loss of terminal sequence	This work	This work
BW0218	León isolate cured of pSCL4 Clear evidence of loss of terminal sequence	This work	This work
BW0219	León isolate cured of pSCL4 Clear evidence of loss of terminal sequence and of chromosome having circularized.	This work	This work
BW0220	León isolate cured of pSCL4 Clear evidence of loss of terminal sequence.	This work	This work

**Table 2.** *
Streptomyces clavuligerus* genome assemblies available from the NCBI as of 15th March 2020 These were extracted from NCBI’s ‘Genome Assembly and Annotation’ and ‘Plasmid Annotation’ reports (https://www.ncbi.nlm.nih.gov/genome/genomes/834) or are reported in this work. All apart from the Broad assembly contain one scaffold per replicon; all PacBio assemblies contain one contig per replicon.

Assembly designation	Strain	Accession no.	Total size (kb)	Replicons as named in the original submission (ungapped length in bp)	No. of genes	No. of proteins	Release date	Sequencing technology	Coverage	Reference
JIC2020	León derivative of ATCC 27064	GCA_015708605.1	8704.802	Chromosome (6748580), pSCL1 (11696), pSCL2 (149702), pSCL4 (1794824)	7227	7012	2020-12-31	PacBio, Illumina	200×	This work
KAIST	ATCC 27064^T^	GCA_005519465	8544.09	Chromosome (6784591), pCLA1 (1795495)	7157	6873	2019-05-20	PacBio	50×	[17]
KRIBB	NRRL 3585^T^	GCA_000148465	9143.38	Chromosome (6736475), pSCL1 (10266), pSCL2 (149326), pSCL3 (442792), pSCL4 (1796117)	7655	7379	2010-09-17	Sanger, 454	80.7×	[14]
Groningen	ATCC 27064^T^	GCA_000163875	8556.89	Chromosome (6735502), pSCL4 (1792895)	7127	6837	2010-04-01	Sanger	11.8×	[13]
Broad	ATCC 27064^T^	GCA_000154925	6941.74	597 contigs in 158 scaffolds (total length 6729086)	–	–	2008-02-28	Unknown	Unknown	NCBI
pSCL1	NRRL 3585^T^	X54107	11.696	pSCL (11696)	6	6	1993-02-03	Sanger	Unknown	[16]
Industrial1	F613-1	GCA_001693675	7590.76	Chromosome (6883702), pSCL4 (707056)	6340	6097	2016-07-26	Illumina, PacBio	60×	[53]
Industrial2	F1D-5	GCA_003454755	8059.12	Chromosome (6900908), pSCL1 (1051520), pSCL2 (106697)	6692	6330	2018-09-05	Illumina, PacBio	320×	NCBI

T, Type strain.

Given the lack of a definitive high-quality genome sequence for a type strain, we set out to establish one for an isolate of the type strain *Streptomyces clavuligerus* ATCC 27064 obtained from the Liras laboratory at the University of León (Spain): this is arguably the most studied isolate of the species. Our *de novo* genome sequence was obtained using Pacific Biosciences RSII SMRT technology (PacBio) and complemented with Illumina paired-end data, and the assembly was verified by optical mapping using Bionano Irys. To assess plasmid content, we additionally sequenced the genomes of five type strain deposits that were obtained directly from four culture collections; this was achieved using Illumina paired-end sequencing. To overcome problems encountered when attempting gene editing in *Streptomyces clavuligerus *, we modified previously published CRISPR-Cas9 vectors and report these herein. To initiate our studies on the maintenance of linear plasmids in *Streptomyces clavuligerus,* these vectors were used to target pSCL4 *parB*, the gene encoding the centromere binding protein ParB essential for plasmid partitioning [24, 25]. This resulted in curing of the megaplasmid, the first example, as far as we are aware, of Cas9-mediated genome editing in *Streptomyces clavuligerus *. Curing the ~1.79 Mb pSCL4 revealed that the plasmid is essential for maintenance of the linear chromosome structure, a defining feature of the genus *Streptomyces *.

## Methods

### Strains and culture conditions

*Streptomyces clavuligerus* ATCC 27064 León isolate was obtained from the laboratory of Professor Paloma Liras at the University of León (Spain). *Streptomyces clavuligerus* type strain deposits ATCC 27064, NRRL 3585, DSM 738 and NCIMB 12785 and NCIMB 14335 were obtained directly from ATCC (American Type Culture Collection), NRRL (Northern Regional Research Laboratory), DSMZ (German Collection of Microorganisms and Cell Cultures) and NCIMB (National Collection of Industrial Food and Marine Bacteria) culture collections, respectively. *Streptomyces clavuligerus* was cultivated using tryptone soya broth (TSB)-agar or TSB-liquid medium (Oxoid) for general cultivation. *Streptomyces clavuligerus* was cultivated in ME (sporulation medium) medium for sporulation [26]. *Escherichia coli* DH5α was used as general-purpose cloning host following established procedures [27]. *E. coli* ET12567/pUZ8002 was used as donor host in *E. coli–Streptomyces* conjugation, following established methods [28]. *Streptomyces coelicolor* M145 [28] was used as a recipient control for conjugation experiments. *Streptomyces clavuligerus* BW0216–BW0220 are pSCL4-cured strains derived from the León isolate during this work.

### Vectors and plasmids

Table S2 provides a list and brief description of vectors used and plasmids constructed during this work, and is followed by a detailed description of the construction of each plasmid made during this work (see Supplementary Material, where a full annotated sequence in GenBank format and detailed graphical representation of the most relevant vectors are also provided). Restriction enzymes, PCR and other molecular biology kits were used according to manufacturers’ instructions.

### DNA sequence analysis software

General visualization, analysis and manipulation of DNA sequence data were performed with computer programs ApE (M. Wayne Davis; https://jorgensen.biology.utah.edu/wayned/ape/), Artemis [29], Artemis Comparison Tool [30] and NotePad++ (http://notepad-plus-plus.org/). Next-generation sequencing reads and contigs mapping was performed with bwa [31, 32] and SAMtools [33] as previously described [34]. Assembly files were visualized with BAMView [35] and their quality was assessed with Qualimap 2.2.1 [36, 37]. Alignments and assembly of sequences were performed with the Staden package [38, 39]. blast+ [40] searches were performed with the NCBI web server (http://www.ncbi.nlm.nih.gov/blast/), or on a standalone computer with prfectBLAST 2.0 [41]. Annotation of gene function and genetic features was performed with rast [42, 43] and antiSMASH [44]. The busco pipeline [45] was used to assess completeness of the genome assembly. Full details about bioinformatics analysis are given in the Supplementary Material.

### Genome sequencing and optical mapping

Illumina sequencing was commissioned to MicrobesNG (IMI – School of Biosciences, University of Birmingham, Birmingham, UK). PacBio sequencing (Pacific Biosciences of California) was commissioned to the Earlham Institute (Norwich Research Park, Norwich, UK) and the Centre for Genomic Research (CGR) of the University of Liverpool (Liverpool, UK). All sequence data reported in this study have been deposited in public databases (Table S1). A fully detailed account of the methodology and data output is provided in the Supplementary Material (data statistics are given in Tables S3, S4, S5).

Optical mapping was performed with Bionano Irys technology (Bionano Genomics). The experimental part (DNA extraction, labelling and data collection, including processing of images to extract BNX files with molecule information) was outsourced to the Genomics facility at Queen Mary University of London (QMUL), London, UK (https://www.qmul.ac.uk/sbcs/research/facilities/genomics-facility/). Data analysis was performed with Bionano’s software IrysView Genomic Analysis Viewer, version 2.5.1.29842, following guidelines and advice from BioNano Technical Support (see the Supplementary Materials for details).

## Results

### Complete *de no*vo genome sequence of *Streptomyces clavuligerus* ATCC 27064 (León isolate)

**Fig. 1.**
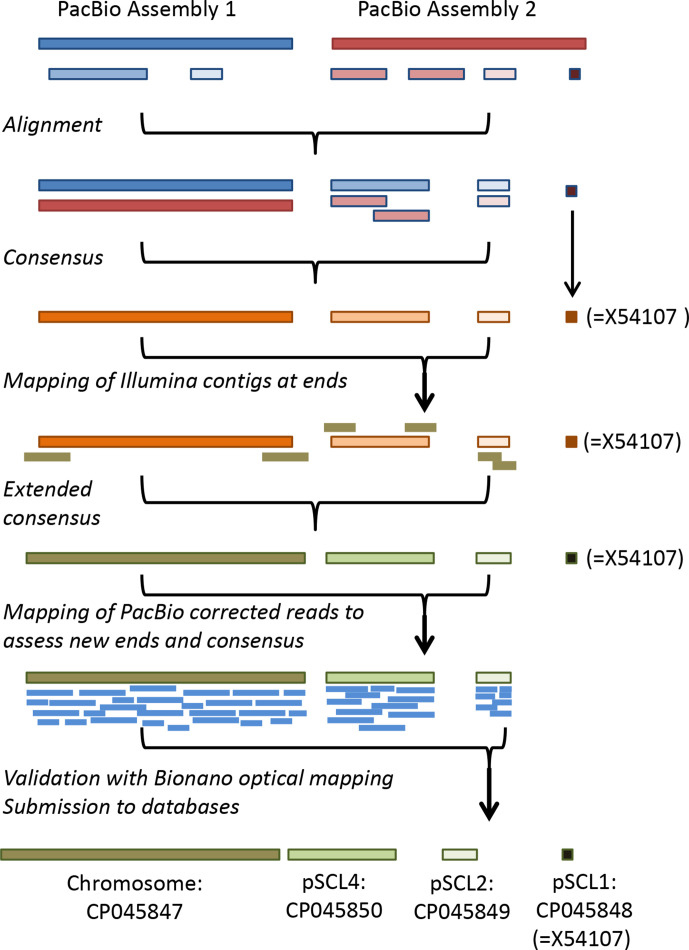
Workflow and strategies leading to the genome sequence reported in this work. The linear plasmid pSCL1 was not fully sequenced by PacBio nor completely assembled by the Illumina pipeline; the read data for both were mapped to the previously published sequence for pSCL1 (accession no. X54107) indicating the full published sequence is present in, and equivalent to, the León isolate.

Fig. 1 illustrates the complete workflow leading to the genome sequence here described (further details are given in Supplementary Materials and Fig. S1). Two independent assemblies were generated using PacBio RSII technology at two different providers using DNA samples extracted from different cultures grown several months apart. By combining these datasets, we obtained single contigs for the chromosome, pSCL2 and pSCL4, and found evidence for pSCL1 in a short 1.4 kb contig [further analysis indicated that the entire 12 kb plasmid was present in the strain (see below)]. The amount of data obtained, with over 200× mean coverage for each assembly, provides a high degree of confidence in the final assembly (see Table S3 for information on sequencing and assembly). No sequence data were obtained for the linear pSCL3, which is present in the KRIBB assembly and in the Broad Institute’s data, but which is absent from all other genome assemblies of *Streptomyces clavuligerus *. We then generated genome sequence data using Illumina 250 nt paired-end reads to a coverage of approximately 200×. Although these data yielded a fragmented assembly of around 1700 contigs, with coverage that was clearly influenced by the use of PCR amplification during library preparation (see Methods), these data proved very useful in allowing us to extend the ends of the linear replicons and to assess the completeness of the pSCL1 assembly.

To build a consensus sequence, we first merged the contigs of both PacBio assemblies. Misassembly problems for pSCL4 in one of the assemblies were resolved by PCR and Sanger sequencing (see Supplementary Material). We then added Illumina contigs found close to the termini of the PacBio assemblies to extend the sequences at the ends of the replicons (the method has been published previously [34] and is explained in the Supplementary Material). The PacBio corrected reads were then mapped back to the new sequences at the extended ends to assess their accuracy (see Fig. 1 for a schematic of the entire process).

Because PacBio uses relatively long DNA fragments, it was unlikely that fragments of the small 12 kb pSCL1 were included in the sequencing library. Despite this, one of the PacBio assemblies yielded a 1.4 kb fragment of pSCL1. Searching the PacBio full data using blastn with the original published sequence for pSCL1 [16] (accession no. X54107) revealed several unassembled corrected-reads that, after alignment, covered almost the entire pSCL1×54107 sequence (with only one possible bp difference identified). Addition of the Illumina assembled contigs to this alignment extended the coverage of the left end of X54107. In a separate analysis, we mapped the Illumina reads to X54107, which resulted in a very-good-quality alignment with only a few differences to the published sequence (which probably reflect errors in the sequencing data, as they were located in regions with very low coverage that arise due to bias of the PCR-based library preparation method) and at the very ends of the linear plasmid where coverage is low. In fact, the Illumina data did not include the last 7 nucleotides of the inverted-repeat at each end of X54107 (5′-CCCGCGG-3′). Taken together, these combined analyses indicate that pSCL1 is present in this strain and that its sequence is identical to that previously published. Thus, although confirmatory, our data does not add any further sequence to that reported in accession number X54107. This sequence data for pSCL1 is consistent with the presence of a DNA fragment of approximately 12 kb in both standard and PFGE-based agarose gels of total DNA obtained from this strain. Therefore, we concluded that the León isolate of the ATCC 27064 type strain carries pSCL1 with the same sequence as previously published [16, 46], the sequence of which (taken from accession number X54107.1) is included in our assembly.

The final length of the sequence for each of the replicons after this process were: chromosome, 6 748 580 bp; pSCL4, 1 794 824 bp; pSCL2, 149 702 bp (which includes a further 254 bp extension to the left end of the PacBio assembly that was obtained from Illumina contigs generated during sequencing of the five pSCL4-cured isolates, see below); pSCL1, 11 696 bp (as in the original deposited sequence X54107.1). These consensus sequences have been deposited at GenBank/ENA/DDBJ through the NCBI submission portal (BioProject PRJNA579405; BioSample SAMN13111356; Assembly GCA_015708605.1; sequence accession numbers CP045847 to CP045850. These data are summarized in Table 2 and compared with previously published assemblies.

This assembly was further analysed using the BUSCO pipeline [45] and the *Streptomycetales* dataset (18th May 2021 dataset). This gave the result C:98.9 % [S:98.6 %, D:0.3 %], F:0.3 %, M:0.8 %, *n*:1579 [1561 complete BUSCOs (C); 1557 complete and single-copy BUSCOs (S); 4 complete and duplicated BUSCOs (D); 4 fragmented BUSCOs (F); 14 missing BUSCOs (M); 1579 total BUSCO groups searched]. A more detailed report is given in the Supplementary Material, but our results provide additional support for the completeness of the deposited assembly.

Despite the essentially complete nature of our genome assembly, we could only unambiguously identify the putative terminal inverted repeats (TIRs) for pSCL2: a blastn search using the first 2 kb of our pSCL2 sequence revealed an inverted repeat of 348 bp with 99 % identity (only four differences). The TIR would run for the first 413 bp at the left end of the reported sequence, with an almost complete repeat (missing the first 65 bp) at the right end.

Finally, we validated our consensus sequence by optical mapping using Bionano Irys and two nickases, Nt.BspQI (GCTCTTC) and Nt.BbvCI (CCTCAGC). Two different bioinformatic analyses were performed. First, the Bionano molecules were aligned to the genome assembly with Bionano IrysView software and the resulting statistical indicators obtained for both enzymes indicated that the Bionano data aligned very well with the reference sequence. Second, the Bionano data were processed using the Bionano Solve pipeline to construct a *de novo* assembly and to assess structural variations. These results highlighted one possible error (a putative artificial insertion in the chromosome assembly) that was discarded after careful examination of the Bionano data, of our assembly, of the translated protein sequence for the affected gene, and of the similarity of our sequence to both previously published genome assemblies (Figures S2 to S5 with full details available in the accompanying text in Supplementary Materials).

### pSCL3 is present in all deposited type strain isolates of *Streptomyces clavuligerus*

The presence of two linear plasmids in *Streptomyces clavuligerus* with approximate lengths of 120 kb (pSCL2) and 430 kb (pSCL3) was first reported by Netolitzky and co-workers following PFGE analysis of the NRRL 3585 type strain deposit [15]. Sequence data for pSCL2 and pSCL3 were first reported by Song and co-workers for the NRRL 3585 type strain deposit as part of the KRIBB assembly [14]. However, the first deposited genome sequence for the *Streptomyces clavuligerus* ATCC 27064 type strain [13] did not include sequences for either plasmid. Other genome sequences from non-type strains of *Streptomyces clavuligerus* (industrial isolates) also lack any sequences for pSCL2 and pSCL3. For the León isolate of ATCC 27064 described here, neither the PacBio data nor the Illumina data contained sequences corresponding to pSCL3, but did contain pSCL2 as described.

Thus, all previous reports describing pSCL2 and pSCL3 used the deposited type strain isolate NRRL 3585, whereas the two assemblies which did not [13, 17], and the one reported here, were all derived after sequencing isolates of the deposited type strain ATCC 27064. To better understand these apparent differences in plasmid content, we obtained the following five isolates of the type strain directly from the respective culture collections: ATCC 27064, NRRL 3585, DSM 738, NCIMB 12785 and NCIMB 14335 (see Table 1).

To assess the presence of pSCL2 and pSCL3, we obtained a shotgun genome sequence for each deposited type strain isolate using Illumina sequencing (250 nt paired-end reads, PCR-based library preparation; coverage between 60× and 100×). The reads were mapped to our consensus sequence for the chromosome, pSCL4 and pSCL2, plus the previously published sequences for pSCL1 (accession no. X54107.1) and pSCL3 (accession no. CM001018.1), and yielded sequence data with complete coverage of all five replicons, including pSCL3 and pSCL2. This contrasts sharply with the Illumina data that we obtained for the León isolate, which contained complete sequences of all the replicons apart from pSCL3. For each deposited isolate, we then calculated the relative coverage of each replicon with respect to that of the chromosome and found differences in apparent copy number between the different isolates, most notably for pSCL3. Coverage for pSCL4 was close to one copy per chromosome across all deposits. For pSCL1 and pSCL2, the coverage varied between 1 and 2 copies per chromosome, whereas for pSCL3 the number ranged from 0.2 copies per chromosome in ATCC 27064 to 1 copy per chromosome in NCIMB 14335 (Table S6).

### Construction of vectors for gene editing in *Streptomyces clavuligerus*

pSCL4 is one of the largest plasmids identified in bacteria and its genetic content has been proposed to have fundamental implications for the biology of the strain [13, 47]. Elimination of pSCL4 from *Streptomyces clavuligerus* has been reported previously, although this was achieved with difficulty using standard genetic manipulation based on homologous recombination [47], and by using both PCR targeting and after multiple rounds of subculture [48]. Thus, for a proof of principle study, we decided to use Cas9-mediated genome editing to cure pSCL4 in a targeted manner.

To achieve this, we first attempted to use the previously reported pCRISPomyces-2 vector [21], but failed to obtain exconjugants in an initial control experiment where we attempted conjugation into *Streptomyces clavuligerus* using the empty vector. As a further test, we introduced pCRISPomyces-2 into the model organism *Streptomyces coelicolor* M145 via conjugation, but the resulting *Streptomyces coelicolor* exconjugant primary colonies also grew poorly. When several of these exconjugants were subcultured in the presence or absence of antibiotic selection, it was revealed that more than half were antibiotic sensitive and had apparently lost the plasmid. This is consistent with observations made for other *Streptomyces* spp.; therefore, we concluded that pCRISPomyces-2 is not suitable for use in *Streptomyces clavuligerus *. We then tested a second reported vector, pCRISPR-Cas9 [20], but the conjugation efficiency with the empty vector was also very low. Therefore, we decided to generate alternative vectors to suit our requirements.

Both previously reported vectors are based on pGM1190, a plasmid vector that utilizes the temperature-sensitive origin of replication of pSG5 [18] and provides a mechanism for its selective curing. Assuming this origin of replication might be problematic in *Streptomyces clavuligerus *, we decided to develop alternative vectors based on the origin of replication of pIJ101 [28]. We chose to transfer the Cas9 functionality from pCRISPomyces-2 into pIJ86 (Table S2), a self-replicative vector with an apramycin-resistance gene *aac(3)IV* for selection. This plasmid replicates in *Streptomyces clavuligerus *, but is lost readily in the absence of selection (as it is in other *Streptomyces* spp.).

**Fig. 2.**
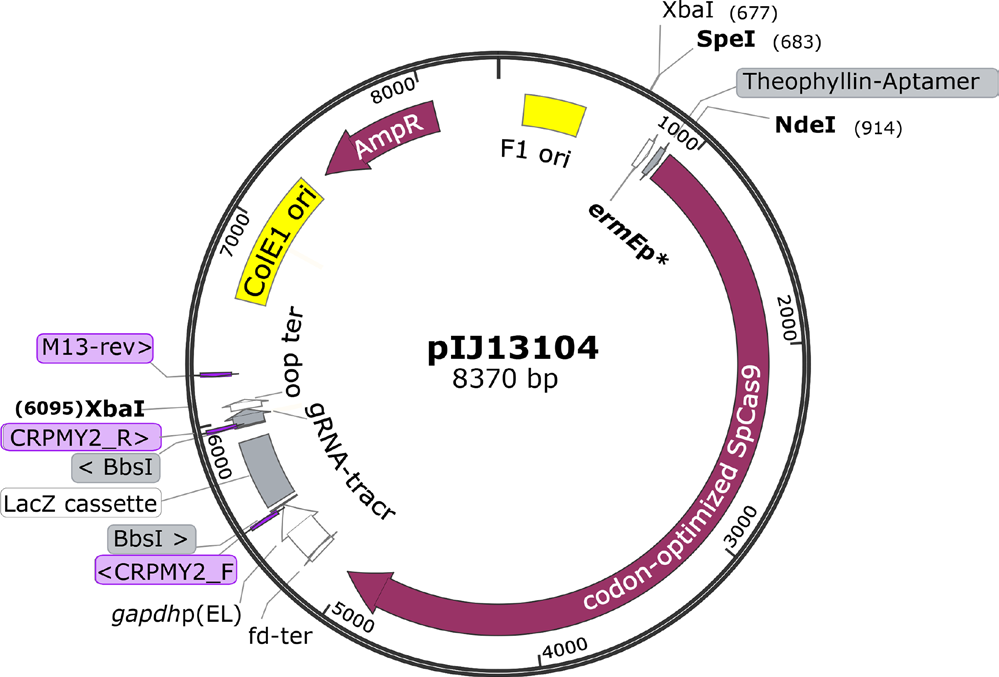
Map of gene editing vector pIJ13104 showing relevant genetic features. Positions of the sequencing primers M13-rev (5′-TCACACAGGAAACAGCTATGAC-3′), CRPMY2_F (5′-ATAAGGCTTGCAGCATCTGG-3′) and CRPMY2_R (5′-CGGTGCCACTTTTTCAAGTT-3′) are shown. Details of the genetic elements inherited from pBluescript II KS+ (ColE1 origin of replication, AmpR ampicillin-resistance gene, F1 origin of ssDNA replication), pCRISPomyces2 [SpCas9 gene, transcriptional promoter *gapdhp*(EL) and terminators fd-ter and oop ter, LacZ cassette, and gRNA-tracr], ermEp* promoter, and theophylline riboswitch can be found in the relevant references [21, 49, 65, 66]. Sites *Spe*I and *Nde*I can be used to exchange the SpCas9 gene promoter; sites *Xba*I or *Xba*I/*Spe*I can be used to excise the full CRISPR cassette.

To retain the ability to clone spacer sequences using Golden-Gate cloning (as with pCRISPomyces-2), we first cloned the Cas9-encoding sequence and *lacZ-tracrRNA* system from pCRISPomyces-2 into pBluescript II KS+. The resulting plasmid pIJ13101 is a versatile intermediate construct that allows insertion of any desired promoter to control the expression of *cas9*. We then added the validated constitutive promoter *ermE*p* followed by a theophylline riboswitch [49, 50] to control the expression of *cas9*. The resulting vector pIJ13104 can then be used to insert the spacer sequence (or sequences) by Golden-Gate cloning using the unique *Bbs*I restriction site as in pCRISPomyces-2. Fig. 2 provides a graphic representation of pIJ13104 (additional details including fully annotated sequences for both plasmids can be found in the Supplementary Material). After addition of the spacer sequence to pIJ13104, the entire *promoter-cas9-sgRNA* cassette can be excised with *Spe*I/*Xba*I and cloned into pIJ86 digested with *Xba*I. This restores a unique *Xba*I site available for insertion of DNA fragments required for homologous recombination. pIJ13105, a pIJ86 derivative with the CRISPR-Cas9 *Spe*I/*Xba*I cassette from pIJ13104 but no spacer sequence (i.e. no targeting functionality), yielded the highest number of exconjugants obtained by us with *Streptomyces clavuligerus* for any CRISPR-Cas9 vector. The plasmid was retained under antibiotic selection, but readily lost when subcultured in the absence of antibiotic.

### Curing of pSCL4 from *Streptomyces clavuligerus* by Cas9-mediated targeting of *parB*

To eliminate pSCL4, we set out to introduce a DNA double-strand break into *parB*, which is essential for high-fidelity partitioning of a single-copy replicon during cell division and sporulation. Without the additional provision of duplicated homologous segments of DNA to repair the double-strand break, we expected the loss of *parB* to result in the loss of pSCL4. A specific spacer sequence was designed discarding any possible off-target recognition sites using blast analyses and our newly derived complete genome sequence (see the Supplementary Material). pIJ13106, which carries the *parB*-targeting cassette, was transferred into *Streptomyces clavuligerus* (León isolate) resulting in many exconjugants, several of which were sub-cultured in the presence and absence of apramycin. Five of the apramycin-sensitive isolates, *Streptomyces clavuligerus* BW0216 to BW0220**,** were selected for further analysis.

The presence of pSCL4 was first assessed using several PCR primer pairs (see the Supplementary Material). All five of the putative pSCL4-cured isolates gave negative results for all of the PCR reactions (with the parental strain used as a positive control). They were then subjected to whole-genome shotgun sequencing using Illumina technology [250 nt paired-end reads, PCR-based library preparation; the full Illumina sequencing data and assembly have been deposited at NCBI (BioProject PRJNA587886)]. Both the reads and the assembled contigs were used for analysis. The trimmed reads from each strain were independently mapped over our complete-genome sequence and the known sequence of pSCL3 (as a negative control since the parental strain had been shown not to carry this plasmid). All five isolates lacked pSCL3 and pSCL4 sequences, while complete coverage of pSCL1 and pSCL2 was obtained (Supplementary Materials and Table S7). These results demonstrated that we had indeed achieved the targeted curing of pSCL4.

### pSCL4 is required for maintenance of the *Streptomyces clavuligerus* linear chromosome

Analysis of the genome assemblies of the pSCL4-cured isolates revealed substantial changes towards the ends of the chromosomes. Deletions were identified in all clones. Strikingly, we obtained clear evidence for chromosome circularization in two of the isolates (BW0216 and BW0219, see below), which yielded reads and assembled contigs that spanned and overlapped the ends of the parental linear chromosome sequence; this was consistent with circularization of the chromosome concomitant with the loss of large terminal segments. The data for the remaining three isolates was insufficient to verify circularization of the genome, but does not rule out the likelihood that this has occurred.

**Fig. 3.**
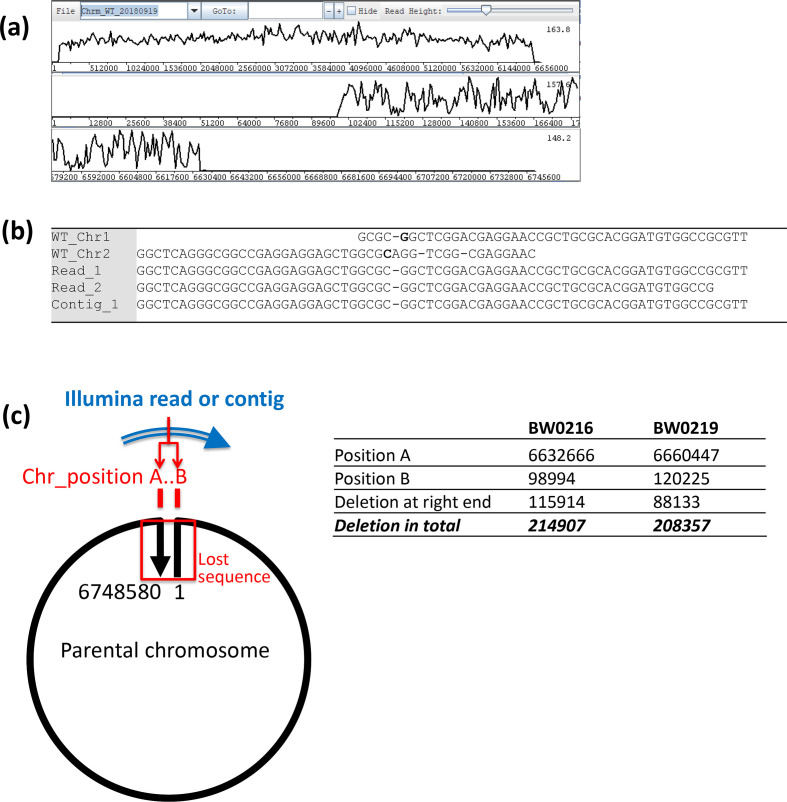
Analysis of chromosome circularization in pSCL4-cured strain BW0216. (a) Plot of Illumina reads for the BW0216 chromosome sequence aligned against the León isolate reference showing a loss of coverage at both termini (top panel); the alignments for the two termini are expanded in middle and bottom panels. (**b**) Comparison of sequence alignments for sections of the reference chromosome sequence ends versus two Illumina reads and one assembled contig that cover the intersection resulting in circularization for BW0216; the bold letters indicate the first and last chromosome positions present in the mutants (a detailed analysis including for all five mutants can be found in the Supplementary Material). (**c**) Interpretation of the blastn results of Illumina contigs and reads overlapping both ends of the chromosome of strains BW0216 and BW0219 along with nucleotide positions and deletion sizes inferred from the analysis.

Alignment of the Illumina reads of BW0216 over the full genome assembly gave no coverage at both ends of the linear chromosomal sequence (Fig. 3a). Detailed analysis revealed a sharp cut-off of the aligned reads around position 98 990 of the left end of the chromosome and around position 6 632 670 of the right end of the chromosome (see the GAP5 alignment in the Supplementary Material). In addition to the Illumina reads alignment, we also identified an assembled contig (NODE_89_length_14998_cov_34.2776) that runs across the same coordinates (Fig. 3b) providing further support for circularization. Taken together, this demonstrates contiguity across positions 98 994 and 6 632 666 of the linear chromosome (Fig. 3c), and indicates that during the process of circularization the first 99 kb and last 116 kb of DNA have been lost from the chromosome ends. By analysing the Illumina reads and contigs data for clone BW0219, we observed a lack of read coverage before position 120 225 and after position 6 660 447 of the parental chromosome, and an assembled contig (NODE_171_length_10901_cov_46.975) that runs across the same positions of the parental chromosome (see Supplementary Material). This again demonstrates contiguity with deletion of the first 120 kb and last 88 kb (Fig. 3c), leading to circularization of the linear replicon.

For the three pSCL4-cured isolates BW0217/218/220, the Illumina whole-genome read and assembled contig data were insufficient for establishing chromosome circularization (we could not identify reads nor contigs that sufficiently and confidently overlap across a putative junction of both ends). However, the data clearly revealed loss of large segments of DNA at the ends of the chromosome, with peaks of coverage that may reflect other rearrangements at one of the ends concomitant with sequence loss. A detailed analysis of the whole-genome shotgun sequencing data of all five clones, including how we deduced chromosome circularization in two of them, is provided in the Supplementary Material.

## Discussion

In this work, we report the most complete genome sequence yet for the type strain of the industrially important *Streptomyces clavuligerus* (Table 2). The assembly was obtained using data from both short- and long-read sequencing methods and validated through physical analysis using Bionano optical mapping. The result of the busco [45] analysis supports a complete assembly based on the general protein-encoding genes expected for *Streptomyces* spp.

Despite the abundance of sequence data, we were not able to identify the starts of the TIRs for the chromosome and pSCL4, potentially indicating that the TIRs of these linear molecules are all relatively short (the size reported in the literature for other *Streptomyces* spp. varies from a probably underestimated 11 bp [51] to over 1 Mb [52]). In our experience, the large DNA fragment size used in PacBio libraries makes it difficult to obtain an assembly approaching the ends of linear replicons, while Illumina assemblies do reach much further towards the ends [34]. The presence of short TIRs would likely cause difficulties for the incorporation of enough DNA representing the TIRs in the whole-genome shotgun libraries, even in the short 500 bp insert Illumina library, and reflects the most likely explanation for the lack of clearly identifiable TIRs in the final replicon assembly.

At the time of writing this paper, a further assembly for the type strain was published as part of a transcriptomics study (accession no. GCA_005519465.1), also derived from the *Streptomyces clavuligerus* ATCC 27064 deposit [17]. The sequences for the chromosome and pSCL4 (called pCLA1 by these authors) are very similar to ours, but this assembly does not report any sequence for pSCL1, pSCL2 or pSCL3 (the lack of pSCL1 can be explained by the small size of the plasmid and its under-representation in the PacBio library, as explained above). We believe, therefore, that the sequence reported here is the most complete and accurate sequence for the chromosome, pSCL2 and pSCL4 of a type strain of *Streptomyces clavuligerus* published thus far (PacBio at over 400× aggregated coverage with optical mapping validation).

A notable feature of our genome assembly is the lack of any sequence for the previously described pSCL3 [14], which had only been identified in one of the three published genome sequences of the type strain. This prompted us to investigate more thoroughly whether pSCL3 was present in type strain isolates obtained directly from each of the four major strain repositories. Whole-genome sequencing confirmed its presence in each isolate, although there was significant variance in the apparent copy number compared to the chromosome. The DNA used for sequencing these five isolates was extracted from liquid cultures inoculated with a fraction of the lyophilized mycelium received from the culture collections. While mechanisms presumably exist to ensure segregation of each of the plasmids into spores, their copy number per chromosome in vegetative mycelial compartments may be quite variable, and in many cases they may be absent. Consequently, the observed differences in coverage and, hence, in apparent copy number of each of the replicons may simply reflect the nature of the mycelial inoculum.

Since we were not able to detect any sequences corresponding to pSCL3 in the PacBio or Illumina data for the León isolate of ATCC 27064 (see above), we presume that the plasmid has been lost at some point after acquisition of the strain from the culture collection, probably during single spore isolation. The absence of pSCL3 from two of the other whole-genome assemblies of this type isolate [5, 7] and both genome assemblies of industrial strains (Table 2) may also reflect the relative instability of this plasmid. In contrast, the assembly reported in this paper contains an almost complete sequence of pSCL2, which was also found in each of the deposited type strains (Table S6) despite its absence again from the other two whole-genome assemblies of the ATCC 27064 type strain isolate [5, 7] and one industrial strain [53]. The sequence of pSCL3 and clarification of its presence in a type strain is the subject of a forthcoming paper regarding the *Streptomyces clavuligerus* type strain deposit DSM 738 (Algora Gallardo *et al*., manuscript in preparation).

Several plasmids for CRISPR-Cas9-based gene editing of *Streptomyces* spp. have been published [20, 21], but their application has not been widely reported. One consequence of the high G+C content of *Streptomyces* genomes (around 70 mol% G+C) is that the protospacer associated motif (PAM) NGG required for a sequence to be recognized as a target by Cas9 occurs by chance at high frequency, and to avoid potentially deleterious off-target cleavage it may be beneficial to maintain the expression of *cas9* at a low level. The CRISPR-Cas9 editing platform we describe here uses the theophylline riboswitch to, in principle, control *cas9* expression. However, as our successful curing of pSCL4 shows that we did not need to add theophylline to induce expression, the constitutive low-level transcription of *cas9* appears sufficient to allow the product nuclease to function efficiently without hampering the development of the resulting exconjugant colony. A further advantage of the pIJ86-based platform is the ready loss of this plasmid when antibiotic selection is removed. While in the process of publishing this work, we were made aware of a recent report using a theophylline riboswitch to control *cas9* expression [54].

We are not the first to report curing of pSCL4 from a *Streptomyces clavuligerus* type strain. Charusanti and co-workers found that loss of pSCL4 following several rounds of selective pressure by co-culture with *Staphylococcus aureus* was likely responsible for the activation of holomycin production [48]. Alvarez-Alvarez and co-workers constructed a pSCL4-cured type strain derivative by deleting *parA-parB* using PCR-targeting [47], and the same group also cured pSCL4 from an *oppA2*-deleted mutant by protoplast regeneration [47] (the same technique reported for *Streptomyces rochei* [55]), a technique demonstrated to lead to unpredictable genetic rearrangements and loss of sequence [56]. Unfortunately, while the data available for these mutants indicates significant loss of sequence and rearrangements near the chromosome termini, they do not allow their topology to be determined. However, based on the results presented here, and those obtained from similar work with *Streptomyces rochei* [37] (see below), we speculate they would also most likely contain circular chromosomes.

It was noted previously that the *Streptomyces clavuligerus* chromosome does not encode any *tap-tpg* homologues [13], a gene-pair essential for the replication of linear replicons in *Streptomyces* [57, 58], but that a *tap-tpg* gene pair was located on pSCL4 [13]. This suggests that replication of the linear chromosome is dependent upon the presence of pSCL4, and is analogous to the situation in *Streptomyces rochei* 7434AN4 in which *tap-tpg* gene pairs are located on two of the three plasmids present in the strain, but not on the chromosome [55]. One of these copies was shown to be required for chromosome replication, and curing of the plasmids led to circularization of the *Streptomyces rochei* chromosome [55, 59]. Our demonstration that pSCL4-cured strains harbour circular chromosomes (two clones confirmed, the other three likely, bearing in mind similar loss of chromosomal terminal sequence) demonstrates that pSCL4, and therefore presumably the encoded *tap-tpg* gene pair, is also required for maintaining the linearity of the chromosome in *Streptomyces clavuligerus *.

Notably, the linear plasmids pSCL2 and pSCL1 were not affected by the loss of pSCL4 in any of the five cured isolates (confirmed by Illumina sequencing). Subsequently, we inspected the pSCL2 sequence and have identified additional copies of the *tap-tpg* gene pair. The pSCL2-encoded proteins have higher amino acid sequence identity to the products of the chromosomal *tap-tpg* gene pair in *Streptomyces coelicolor* than do the pSCL4-encoded *tap-tpg* (see Supplementary Material and [60]). This suggests that there are two pairs of *tap-tpg* genes for the replication of linear replicons in *Streptomyces clavuligerus *, one encoded by pSCL4 and responsible for the correct replication of pSCL4 and the chromosome, and a second pair encoded by pSCL2 and responsible for its replication and that of pSCL1, which does not encode any putative *tap-tpg* homologues. The possibility that some pSCL4-cured mutants might not have undergone chromosome circularization, although the current data does not rule it out, might indicate a role of pSCL2-encoded Tap-Tpg in chromosome replication. However, this hypothesis would need further investigation.

Taken together, our observations suggest that pSCL4 is essential for the maintenance of a linear chromosome in *Streptomyces clavuligerus* and is likely to be required for correct morphological development as also reported by Charusanti and co-workers [48] and Alvarez-Alvarez and co-workers [47]. It is likely, however, that characteristics such as a lack of sporulation could reflect loss of vigour or impaired replication due to loss of sequence close to the chromosome termini or specific genes located within the lost sequence, and not reflect a specific role of pSCL4 in sporulation. Large pleiotropic effects are also likely, bearing in mind these mutants have lost over 20 % of the genome. Since impaired morphological development has been observed upon deletion or mutation of a single gene involved in pleiotropic regulation [11, 61, 62] and even genes involved in antibiotic production but not in sporulation [63], it is not prudent to suggest single genes responsible for the lack of sporulation in our pSCL4-cured mutants. Moreover, none of the five mutants have the same genotype, having lost different segments of the chromosomal ends, and other rearrangements pointed at, but not resolved, by the Illumina data. It is, therefore, necessary to further study the possibility of curing pSCL4 without provoking circularization of the chromosome, loss of chromosomal sequence or impairment in morphological development. Whatever the reason for the observed phenotype, our demonstration that directly targeting double-strand breaks to *parB* genes leads to loss of plasmid replicons should facilitate further studies of *Streptomyces* chromosome structure, an area of significant industrial and fundamental biological interest.

## Supplementary Data

Supplementary material 1
