# Supplementary material for: Genome editing reveals that pSCL4 is required for chromosome linearity in Streptomyces clavuligerus
Source: Microb Genom. 2021 Nov 8;7(11):000669. doi: 10.1099/mgen.0.000669 (PMC8743545; doi:10.1099/mgen.0.000669)
Supplement: Supplementary material 1 [file mgen-7-0669-s001.pdf]

Supplementary Material to "Genome editing reveals that pSCL4 is required for chromosome linearity in *Streptomyces clavuligerus*" by J. P. Gomez-Escribano, L. Algora Gallardo, K. A. J. Bozhüyük, S. G. Kendrew, B. D. Huckle, N. Crowhurst, M. J. Bibb, A. J. Collis, J. Micklefield, P. R. Herron and B. Wilkinson

Table of Contents

Access to the sequence data reported in this work 2

MATERIALS AND METHODS 2

- **Strains and culture conditions 2**
- **Vectors and plasmids 3**

GENOME SEQUENCING 4

- **PacBio RSII SMRT 4**
- **Illumina 5**
- **Sequence data analysis 5**
- **Merging of PacBio assemblies and extension with Illumina data 5**

BUSCO ANALYSIS 7

BIONANO OPTICAL MAPPING 10

- **HMW DNA extraction 10**
- **DNA labelling and data collection 10**
- **Bionano data analysis 10**
- **Resolution of deletions called by Bionano Solve De-Novo Assembly and Structural Variant Analysis .. 12**

ASSESSMENT OF PRESENCE OF pSCL3 IN TYPE-STRAIN DEPOSITS 18

- **ATCC 27064 Leon-isolate 18**
- **ATCC 27064 culture collection deposit 19**
- **DSM 738 culture collection deposit 19**
- **NRRL 3585 culture collection deposit 20**
- **NCIMB 12785 culture collection deposit 20**
- **NCIMB 14335 culture collection deposit 21**

CURING OF pSCL4 23

- **PCR test of pSCL4-cured candidate strains 23**

Analysis of Illumina whole-genome sequencing of pSCL4-cured clones 23

- **Analysis of BW0216 .... 24**
- **Analysis of BW0217 .... 27**
- **Analysis of BW0219 .... 33**
- **Analysis of BW0220 .... 37**

ANALYSIS OF ADDITIONAL *tpg-tap* GENES IN pSCL2 40

Sequence of relevant vectors useful as genetic tools (in Genbank format) and graphical representation of relevant features. 46

- **pIJ13103 .... 46**
- **pIJ13104 .... 51**

Access to the sequence data reported in this work

Table S1. The Whole Genome Shotgun projects described in this paper have been deposited at DDBJ/ENA/GenBank through the NCBI Submission Portal. The Illumina trimmed reads have been deposited at NCBI's Sequence Read Archive (SRA). This table provides the information required to access the DNA sequences and other specific details of the projects.

<table>
<tr><td colspan="5">Streptomyces clavuligerus ATCC 27064 Leon-isolate - High-quality genome assembly
BioProject Accession: PRJNA579405; BioSample Accession: SAMN13111356
The version described in this paper is version 1 (e.g. CP045847.1)</td></tr>
<tr><td>Accession</td><td>Replicon</td><td colspan="3">Sequence version deposited</td></tr>
<tr><td>CP045847</td><td>Chromosome</td><td colspan="3">Chrm_WT_20180919</td></tr>
<tr><td>CP045848</td><td>pSCL1</td><td colspan="3">pSCL1_X54107_20180919</td></tr>
<tr><td>CP045849</td><td>pSCL2</td><td colspan="3">pSCL2_extended_20190410</td></tr>
<tr><td>CP045850</td><td>pSCL4</td><td colspan="3">pSCL4_WT_20180927</td></tr>
<tr><td></td><td></td><td colspan="3"></td></tr>
<tr><td colspan="5">Streptomyces clavuligerus type-strain deposits whole-genome sequencing
BioProject Accession: PRJNA587782
The version described in this paper is version 1 (i.e. XXXX01000000)
MicrobesNG project site: https://microbesng.com/portal/projects/A9708D93-8EA4-4196-A23A-A3ADAF0CF7E5/</td></tr>
<tr><td>BioSample Accession</td><td>Genome Accession</td><td>Strain</td><td>MicrobesNG reference (barcode)</td><td>Sequence Read Archive (SRA)</td></tr>
<tr><td>SAMN13111356</td><td>WKJT00000000</td><td>Leon-isolate</td><td>22226</td><td>SRX7098215</td></tr>
<tr><td>SAMN13218685</td><td>WKJU00000000</td><td>ATCC 27064</td><td>22227</td><td>SRX7098216</td></tr>
<tr><td>SAMN13218686</td><td>WKJV00000000</td><td>NRRL 3585</td><td>22228</td><td>SRX7098217</td></tr>
<tr><td>SAMN13218687</td><td>WKJW00000000</td><td>DSM 738</td><td>22229</td><td>SRX7098218</td></tr>
<tr><td>SAMN13218688</td><td>WKJX00000000</td><td>NCIMB 12785</td><td>22230</td><td>SRX7098219</td></tr>
<tr><td>SAMN13218689</td><td>WKJY00000000</td><td>NCIMB 14335</td><td>22226</td><td>SRX7098220</td></tr>
<tr><td colspan="5">Streptomyces clavuligerus pSCL4-cured derivates from the Leon-isolate of type-strain
BioProject Accession: PRJNA587886
The version described in this paper is version 1 (i.e. XXXX01000000)
MicrobesNG project site: https://microbesng.com/portal/projects/185DC9A4-8DEC-2C49-BB1F-03FA37FB6D6A/</td></tr>
<tr><td>BioSample Accession</td><td>Genome Accession</td><td>Strain (clone)</td><td>MicrobesNG reference (barcode)</td><td>Sequence Read Archive (SRA)</td></tr>
<tr><td>SAMN13220697</td><td>WMCC00000000</td><td>BW0216 (A1)</td><td>22856</td><td>SRX7101996</td></tr>
<tr><td>SAMN13220698</td><td>WMCB00000000</td><td>BW0217 (B4)</td><td>22857</td><td>SRX7101997</td></tr>
<tr><td>SAMN13220699</td><td>WMCA00000000</td><td>BW0218 (C5)</td><td>22858</td><td>SRX7101998</td></tr>
<tr><td>SAMN13220700</td><td>WMBZ00000000</td><td>BW0219 (D11)</td><td>22859</td><td>SRX7101999</td></tr>
<tr><td>SAMN13220701</td><td>WMBY00000000</td><td>BW0220 (E16)</td><td>22860</td><td>SRX7102000</td></tr>
</table>

MATERIALS AND METHODS

Strains and culture conditions

S. clavuligerus ATCC 27064 Leon-isolate was obtained from the laboratory of Prof. Paloma Liras at the University of Leon (Spain), and is the isolate sourced in January 2005 and used for the studies on the regulation of antibiotic production by the stringent response in *S. clavuligerus* [1, 2] undertaken at the University of Leon (Leon, Spain) and the John Innes Centre (Norwich, UK). *S. clavuligerus* type strain deposits ATCC 27064, NRRL 3585, DSM 738, and NCIMB 12785 and NCIMB 14335 were obtained directly

from ATCC, NRRL, DSMZ and NCIMB culture collections respectively. *S. clavuligerus* was cultivated using TSB-agar or TSB-liquid medium for general cultivation (Tryptone Soya Broth, Oxoid CM0129). *S. clavuligerus* was cultivated in ME medium for sporulation [3]. *Escherichia coli* DH5alpha was used as general-purpose cloning host following established procedures [4]. *E. coli* ET12567/pUZ8002 was used as donor host in *E. coli-Streptomyces* conjugation following established methods [5]. *Streptomyces coelicolor* M145 [5] was used as recipient control for conjugation experiments. *S. clavuligerus* BW0216 to BW0220 are pSCL4-cured strains derived from the Leon-isolate during this work.

Vectors and plasmids

Table S2: List and brief description of vectors used and plasmids constructed during this work.

Plasmid	Description	Reference or source
pBluescript II KS(+)	General cloning vector	[6]
pGM1190	RK2 conjugative, apramycin resistance, pSG5 temperature-sensitive origin of replication	[7]
pCRISPomyces-2	Vector for CRISPR-Cas9 based genome editing in *Streptomyces*	[8]
pCRISPR-Cas9	Vector for CRISPR-Cas9 based genome editing in *Streptomyces*	[9]
pGusT-ermEp1-E*	Source of ermE*-theophylline riboswitch	[10, 11]
pIJ86	Conjugative (*oriT* from RK2) self-replicative (pIJ101 origin) expression vector for *Streptomyces*, with *ermE**p, apramycin resistance gene (*aac(3)IV*),	JIC StrepStrains jic.strepstrains@jic.ac.uk
pIJ13103	pBluescript II KS(+) derivative with *lacZ-tracrRNA* system from pCRISPomyces-2	This work
pIJ13104	pIJ13103 derivative with the promoter *ermE*p* and theophylline riboswitch driving *cas9* expression	This work
pIJ13105	pIJ86 derivative with CRISPR-Cas9 functionality from pIJ13104 but no spacer	This work
pIJ13106	pIJ86 derivative with CRISPR-Cas9 functionality to target pSCL4-*parB*	This work
pIJ13107	pCRISPomyces-2 with *cas9* deleted	This work

Construction of **pIJ13103:** the Cas9 coding sequence and *lacZ-tracrRNA* system was PCR-amplified from pCRISPomyces-2 with primers JP212 (5'-CTGGCCTCTAGATAAAAAACGC-3'; anneals at the unique XbaI site, underlined, of pCRISPomyces-2) and JP237 (5'-ACTAGT*CATATG*GACAAGAAGTACAG-3'; anneals at *cas9* start codon and carries sites for SpeI, underlined, and NdeI, italics). The PCR product was blunt-cloned in SmaI-digested pBluescript II KS+; the desired orientation with *cas9* start codon at the M13-forward primer (5'-CGCCAGGGTTTTCCCAGTCACGAC-3') annealing end of pBluescript MCS was selected. A sequence in Genbank format is provided at the end of this document.

Construction of **pIJ13104:** the constitutive promoter *ermE*p* followed by the theophylline riboswitch from pGusT-ermEp1-E* [10, 11] was PCR-amplified with primers JP239 (5'-TTCATATGGTTGCCTCCTTAGCAGG-3'; NdeI site underlined) and JP240 (5'-AAACTAGTGGCCTTTTGCTCACATGTAA-3'; SpeI site underlined). The PCR

product was digested with NdeI and SpeI and cloned upstream *cas9* in pIJ13103 digested with the same enzymes. The desired genetic arrangement was assessed by Sanger sequencing with primer JP230 (5'-ATGGTGGGGTACTTCTCGTG-3'; anneals at 5'-end of *cas9*, for sequencing of promoter) and the sequencing assessment of several candidate clones revealed that there is an SpeI site present in the PCR product that was not reported in the original sequence of pGusT-ermEp1-E* and that led to the loss of the fd-terminator in many clones. The clone chosen as pIJ13104 keeps the full fd-terminator as originally designed.

Construction of **pIJ13106:** the spacer sequence 5'-CCCCGACGAAATACTGGAAT-3', which is followed by a canonical PAM motif CGG, was identified within the codding sequence of *parB* (CDS with locus_tag="GE265_32075" in the published sequence accession CP045850) with CRISPy-web (https://crispy.secondarymetabolites.org/). We used the sequence for a BLASTn search against our full genome assembly and discarded any off-target position (the best BLASTn hits were CCCCGACGACATTCTCGAAT, with 3 mistatches, in pSCL4, and CCCCGACGGACTGCTGGAA with 3 mismatches in the chromosome; neither of them was followed by the required NGG PAM motif. No hit as close as these was found in our sequence of either pSCL1 or pSCL2, or in the known sequence of pSCL3). The oligonucleotides JP216 (5'-acgcCCCCGACGAAATACTGGAAT-3') and JP217 (5'-aaacATTCCAGTATTTCGTCGGGG-3') were annealed as described in the original pCRISPomyces-2 publications [8, 12] and cloned into pIJ13104 digested with BbsI. A correct clone was chosen after assessment by Sanger sequencing with universal primers M13-reverse (5'-TCACACAGGAAACAGCTATGAC-3') and custom primers CRPMY2_F (5'-ATAAGGCTTGCAGCATCTGG-3') and CRPMY2_R (5'-CGGTGCCACTTTTTCAAGTT-3'). The targeting cassette was excised by digestion with SpeI and XbaI and cloned into XbaI-digested pIJ86 to generate construct pIJ13106.

Construction of **pIJ13107:** pCRISPomyces-2 was digested with BglII and the large restriction fragment re-ligated, resulting in the deletion of most of *cas9* coding sequence.

GENOME SEQUENCING

PacBio RSII SMRT

PacBio sequencing (Pacific Biosciences of California, Inc) was commissioned to two different providers: the Earlham Institute (Norwich Research Park, Norwich, NR4 7UZ, United Kingdom) and the Centre for Genomic Research (CGR) of the University of Liverpool (Crown Street, Liverpool, L69 7ZB, United Kingdom). High molecular weight DNA samples were extracted from early-stationary-phase cultures in TSB medium, following the Salting-out protocol as described previously [13]. Sample quality was first assessed by standard agarose-gel electrophoresis and pulse-field gel electrophoresis, and quantification was performed with NanoDrop (Thermo Fisher Scientific) and Qubit (Thermo Fisher Scientific). Samples sequenced at Earlham Institute were extracted on January 2018 and samples sequenced at Liverpool CGR were extracted on June 2018. The sequencing at Earlham Institute was performed with C4-P6 chemistry on three SMRT cells; the data were processed with HGAP4, assembled with Falcon and polished with Quiver. At Liverpool CGR sequencing was also performed with C4-P6 chemistry on two SMRT cells; data was processed with HGAP4, assembled with Canu and polished with Arrow. Table S2 contains a summary of the data output from each sequencing provider.

Illumina

Illumina sequencing was commissioned to MicrobesNG (IMI - School of Biosciences, University of Birmingham, Edgbaston, BIRMINGHAM, B15 2TT, United Kingdom). Briefly, as extracted from the full protocol accessed at https://microbesng.uk/documents/5/MicrobesNG_Methods_Document_-_PDF.pdf on 5th May 2019: PCR-based libraries were prepared using Nextera XT Library Prep Kit (Illumina, San Diego, USA) and sequenced on an Illumina HiSeq 2500 using a 250bp paired end protocol. Reads were adapter trimmed using Trimmomatic 0.30 with a sliding window quality cutoff of Q15. De novo assembly was performed on samples using SPAdes version 3.7, and contigs were annotated using Prokka 1.11. Tables S3a and S3b provide information about the output and quality of assemblies.

Sequence data analysis

General visualisation, analysis, and manipulation of DNA sequence data was performed with computer programs ApE (M. Wayne Davis, https://jorgensen.biology.utah.edu/wayned/ape/), Artemis [14], Artemis Comparison Tool [15], and NotePad++ (http://notepad-plus-plus.org/). Next-generation sequencing reads and contigs mapping was performed with BWA [16, 17] and SAMtools [18] as previously described [13]. Assembly files were visualised with BAMView [19] and their quality was assessed with Qualimap 2.2.1 [20, 21]. Alignments and assembly of sequences was performed with Staden Package [22, 23]. BLAST+ [24] searches were performed at the NCBI web server (http://www.ncbi.nlm.nih.gov/blast/), or on a standalone computer with prfectBLAST 2.0 [25]. Annotation of gene function and genetic features was performed with RAST [26, 27] and antiSMASH [28].

Merging of PacBio assemblies and extension with Illumina data

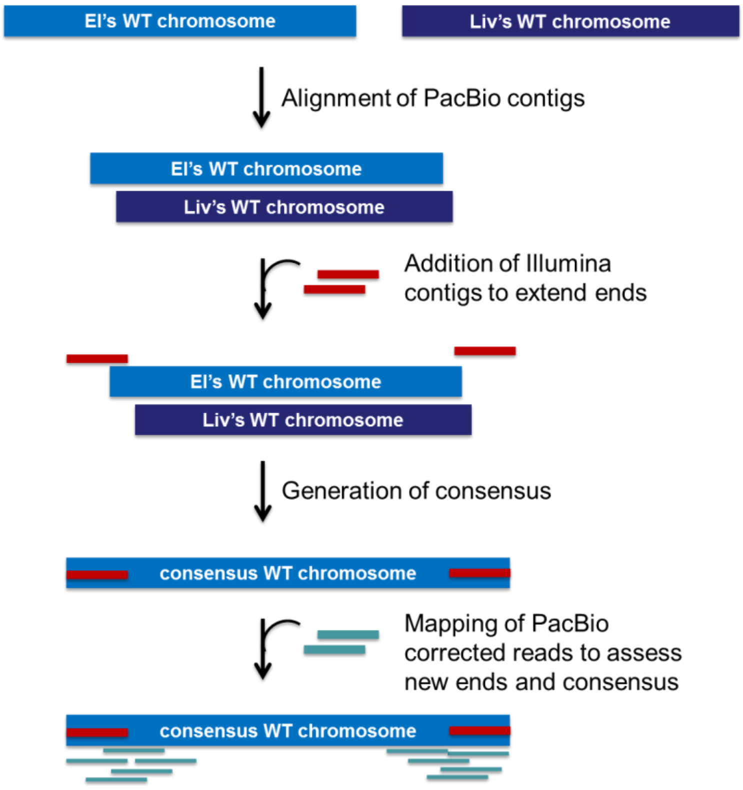

To achieve the final published sequences, contigs from both PacBio assemblies were aligned with Staden Package. Illumina contigs matching the ends of the contigs were identified by BLAST with the last 5 kb of each contig and added to the alignment in Staden Package. PacBio corrected reads matching the fully extended ends were added to the alignment; this confirmed the correctness of the sequence but did not add any extra sequence at the ends. The process is depicted in the following illustration and was performed for each independent replicon (Fig S1, left).

Figure S1. Scheeme illustrating the strategy to extend the PacBio assemblies with Illumina data.

One of the PacBio assemblies reported pSCL4 in two contigs (see Table S3). Alignment of all three pSCL4 contigs showed that the first 21 kb of one of the contigs is a stretch of chromosome sequence, while the rest of the contig perfectly matches the expected stretch of pSCL4 with extensive overlap with the other contigs. The possibility of such a genomic arrangement was probed by PCR and Sanger-sequencing with oligonucleotides spanning the intersection (from 5' to 3': JP402 CTCCTGGACGTCAAGCTCTC, JP403 GTATCCCTCGTCGTCATCGT, JP404 GGTCCGGGTCTCCAAGTATC, JP405 GCCATCTGACGAAGCAACTC, JP406 TCCGGTACTACGGGTGTCTC, JP407 ATATGGCACTGGAGCTGGAC, JP408 GACTGAACCTGCGGAATCTC). The different combinations of these oligonucleotides allow for 4 primer-pairs for the possible chromosomal insertion (and 8 primer-pairs for the lack of such as control). None of the reactions gave any PCR product that would support the existence of such arrangement and was therefore deemed to be a miss-assembly problem.

Table S3. Summary of the output for each PacBio genome assembly project. Due to the different pipeline used for raw-data processing and assembly, the information originates from different software and receives different terminology.

	**Earlham Institute (EI)**	**Liverpool CGR (Liv)**
**Sequencing information**	**(HGAP realigned reads info)**	**(Canu post-filter info)**
Number of SMRT cells	3	2
Polymerase Read Length Mean	7,250	9,870; 8,943
Polymerase Read N50	11,054	14,454; 12,748
Polymerase Read Length Max (CGR, original raw read)	41,132	51,474
Subread Length Mean (CGR, per cell)	4,998	8,263; 7,961
Subread Length N50 (CGR, per cell)	7,013	12,680; 11,707
Subread Length Max (CGR, Canu corrected read raw/filtered_subreads_file)	34,514	49,704/53,865
Number of Polymerase Reads	329,664	202,713
Number of Subreads	455,845	234,680
Subread bases (CGR, SMRT report totalised)	2,278,323,303	1,904,763,227
**Assembly output**		
No. contigs	3	5
total length	8678058	8707940
max length	6743022	6735045
N50	6743022	6735045
N90	1790659	868878
Mean coverage	239,24x	218.74x
Contigs, as matching published replicons (original name, size in bp)		
Chromosome (6.8 Mb)	000000F, 6743022	tig00000001, 6735045
pSCL1 (12 kb)	Not reported	tig00000048, 1352
pSCL2 (120-150 kb)	000002F, 144377	tig00000010, 144708
pSCL3 (430-444 kb)	Not reported	Not reported
pSCL4 (1.8 Mb)	000001F, 1790659	tig00000005, 868878<br>tig00000006, 957957

Table S4. Summary of the trimmed reads output for each Illumina genome assembly project.

Sample id	Median insert size	Mean coverage	Mean coverage excluding 0s	Number of reads	Number of reads with insert size >300
Leon isolate	495	193.633	193.72	3593366	1114928
ATCC27064	438	82.4088	82.4208	1661343	468457
NRRL3585	437	61.571	61.5759	1287697	323704
DSM738	488	57.6536	57.6595	1149441	319085
NCIMB12785	459	67.9208	67.9301	1390146	364297
NCIMB14335	442	93.4944	93.509	1850738	510268

Table S5. Summary of the assembled contigs output for each Illumina genome assembly project.

**Sample id**	**Leon isolate**	**ATCC27064**	**NRRL3585**	**DSM738**	**NCIMB12785**	**NCIMB14335**
**# contigs (>= 0 bp)**	1895	1693	1936	1716	1852	1942
**# contigs (>= 1000 bp)**	1401	1385	1578	1440	1504	1552
**Total length (>= 0 bp)**	8440590	8857537	8816681	8819387	8834566	8836090
**Total length (>= 1000 bp)**	8174273	8665395	8586420	8634123	8614893	8591200
**# contigs**	1648	1595	1833	1650	1733	1809
**Largest contig**	54481	50814	46207	49137	50513	56188
**Total length**	8352264	8817879	8773206	8791680	8785114	8780621
**GC (%)**	72.27	72.26	72.23	72.24	72.23	72.22
**N50**	8784	9600	7716	9059	8460	8038
**N75**	4435	4918	4067	4653	4403	4142
**L50**	274	275	322	282	304	313
**L75**	611	597	716	625	670	690
**# N's per 100 kbp**	0	0.01	0.02	0	0	0.01

BUSCO ANALYSIS

The final assembly as deposited on NCBI database was run through the BUSCO pipeline [29] to assess completeness. The “short summary” as outputted by the pipeline was:

```
# BUSCO version is: 5.1.2
# The lineage dataset is: streptomycetales_odb10 (Creation date:
2020-03-06, number of genomes: 145, number of BUSCOs: 1579)
# Summarized benchmarking in BUSCO notation for file
/busco_wd/GCA_015708605.1_ASM1570860v1_genomic.fna
# BUSCO was run in mode: genome
# Gene predictor used: prodigal
    ***** Results: *****
    C:98.9%[S:98.6%,D:0.3%],F:0.3%,M:0.8%,n:1579
    1561    Complete BUSCOs (C)
    1557    Complete and single-copy BUSCOs (S)
    4    Complete and duplicated BUSCOs (D)
    4    Fragmented BUSCOs (F)
    14    Missing BUSCOs (M)
    1579    Total BUSCO groups searched
Dependencies and versions:
    hmmsearch: 3.2
    prodigal: 2.6.3
```

The missing BUSCOs IDs were annotated with the OrthoDB [30] website https://www.orthodb.org/ (on 18th May 2021):

pub_og_id	og_name	level_taxid	organism_taxid	organism_name	int_prot_id	pub_gene_id	description
29386at85011	Tetrapyrrole biosynthesis,	85011	1888_0	Streptomyces albus	1888_0:0007d4	WP_030770335.1	Transcriptional regulator
30999at85011	oxidoreductase	85011	1888_0	Streptomyces albus	1888_0:001562	WP_030305336.1	Oxidoreductase
32769at85011	4Fe-4S ferredoxin, iron-sulphur binding,	85011	1888_0	Streptomyces albus	1888_0:001650	SFR_RS29525	Succinate dehydrogenase iron-sulfur protein
3311at85011	Fumarate reductase/succinate	85011	1888_0	Streptomyces albus	1888_0:00164f	SFR_RS29520	Succinate dehydrogenase flavoprotein subunit
40785at85011	Tetratricopeptide-like helical domain	85011	1888_0	Streptomyces albus	1888_0:00103f	SFR_RS21810	C1 regulatory protein
42296at85011	Luciferase-like domain	85011	1888_0	Streptomyces albus	1888_0:000b98	WP_061404589.1	Luciferase
45026at85011	Histidine N-alpha-methyltransferase	85011	1888_0	Streptomyces albus	1888_0:001655	egtD	Histidine N-alpha-methyltransferase
47468at85011	Succinate dehydrogenase	85011	1888_0	Streptomyces albus	1888_0:00164e	WP_010639588.1	succinate dehydrogenase
50546at85011	Putative adhesin	85011	1888_0	Streptomyces albus	1888_0:0006ae	WP_053933972.1	A0A126Y1S4_9ACTN
50637at85011	N-acetylmuramoyl-L-alanine amidase	85011	1888_0	Streptomyces albus	1888_0:0011e0	WP_003947809.1	Cell wall hydrolase/autolysin
65544at85011	Lipoprotein						
68949at85011	GNAT domain	85011	1888_0	Streptomyces albus	1888_0:000baf	WP_061404592.1	Transcriptional regulator
75619at85011	Thioredoxin	85011	1888_0	Streptomyces albus	1888_0:000147	SFR_RS01655	Thiol-disulfide isomerase/thioredoxin
80808at85011	Dodecin	85011	1888_0	Streptomyces albus	1888_0:001653	SFR_RS29540	A0A126YH09_9ACTN

BUSCO analysis of the genome without pSCL4:

```
# BUSCO version is: 5.1.2
# The lineage dataset is: streptomycetales_odb10 (Creation date:
2020-03-06, number of genomes: 145, number of BUSCOs: 1579)
# Summarized benchmarking in BUSCO notation for file
/busco_wd/GCA_015708605.1_ASM1570860v1_genomic_NOpSCL4.fna
# BUSCO was run in mode: genome
# Gene predictor used: prodigal

  ***** Results: *****

  C:98.8%[S:98.6%,D:0.2%],F:0.3%,M:0.9%,n:1579
  1560  Complete BUSCOs (C)
  1557  Complete and single-copy BUSCOs (S)
  3     Complete and duplicated BUSCOs (D)
  4     Fragmented BUSCOs (F)
  15    Missing BUSCOs (M)
  1579  Total BUSCO groups searched

Dependencies and versions:
  hmmsearch: 3.2
  prodigal: 2.6.3
```

Only one ortholog group is present in pSCL4 and not the rest of the genome, which does not provide any further indication about its role (Group 59148at85011 annotated as “Pentapeptide repeat”).

BUSCO analysis of pSCL4 alone:

```
# BUSCO version is: 5.1.2
# The lineage dataset is: streptomycetales_odb10 (Creation date:
2020-03-06, number of genomes: 145, number of BUSCOs: 1579)
# Summarized benchmarking in BUSCO notation for file
/busco_wd/CP045850.1_pSCL4.fna
# BUSCO was run in mode: genome
# Gene predictor used: prodigal

  ***** Results: *****

  C:0.9%[S:0.8%,D:0.1%],F:0.0%,M:99.1%,n:1579
  15    Complete BUSCOs (C)
  13    Complete and single-copy BUSCOs (S)
  2     Complete and duplicated BUSCOs (D)
  0     Fragmented BUSCOs (F)
  1564  Missing BUSCOs (M)
  1579  Total BUSCO groups searched

Dependencies and versions:
  hmmsearch: 3.2
  prodigal: 2.6.3
```

BIONANO OPTICAL MAPPING

Optical mapping was performed with Bionano Irys technology (Bionano Genomics, San Diego, USA). The experimental part (DNA-extraction, labelling and data collection including processing of images to extract BNX files with molecules information) was outsourced to the Genomics facility at Queen Mary University of London (QMUL; https://www.qmul.ac.uk/sbcs/research/facilities/genomics-facility/, Mile End Road, London E1 4NS) and performed by Martin Tran. Early stationary phase mycelium from a TSB-liquid culture was harvested by centrifugation at the John Innes Centre, washed with glycerol 20%, resuspended in glycerol 20%, fast-frozen in liquid nitrogen, and shipped to QMUL in dry-ice, where the samples were processed with the following methods.

HMW DNA extraction

Methodology kindly provided by Martin Tran from QMUL

High Molecular Weight (HMW) DNA was extracted following the Bionano Prep Cell Culture DNA Isolation Protocol (Bionano Genomics 30026). *Streptomyces spp.* cells were pelleted, washed and resuspended in Cell Suspension Buffer (Bionano Genomics, 20340). Cells were then embedded in a thin layer of 2% agarose (Bio-Rad) plugs. Bacterial cell wall was removed by incubating the agarose plugs with lysozyme (2mg/ml Sigma-Aldrich, cat. no. L6876) and achromopeptidase (1mg/ml Sigma-Aldrich, cat. no. A3547) in Cell Suspension Buffer at 37 °C for 2 hours with intermittent swirling. The cells were then lysed with Puregene Proteinase K (Qiagen, cat. no, 158920) and Puregene RNase A (Qiagen, cat. no. 158924) in Lysis Buffer (Bionano Genomics). The plugs were washed 7 times with 1x Wash Buffer (Bionano Genomics) and five times with TE buffer, then solubilised using Agarase (Thermo Fisher, cat. no. EO0461). HMW DNA was purified further by drop dialysis (Millipore, #VCWP04700) with TE buffer for 1 hour. DNA was homogenised overnight and quantified with Qubit dsDNA BR Assay Kit (ThermoFisher Scientific, cat. no. Q32853)

DNA labelling and data collection

Methodology kindly provided by Martin Tran from QMUL

HMW DNA was labelled by following the Bionano Prep Labeling NLRS protocol (30024) with the DNA Labelling Kit NLRS (Bionano Genomics, cat. no. 80001). 300ng of DNA was nicked with one of the following; 10 units of Nt.BspQI (New England BioLabs cat. no. R0644S) or 4 units of Nb.BbvCI (New England BioLabs, cat. no. R0631S). The nicked DNA was labelled with fluorescent-dUTP nucleotide analog (Bionano Genomics) using Taq DNA polymerase (New England BioLabs, cat. no. M0208S), then repaired with Taq DNA Ligase (New England BioLabs, cat. no. M0267S). The DNA backbone was then stained overnight withYOYO-1 (Bionano Genomics, cat. no. 80001). The labelled and stained DNA was loaded onto an Irys Chip (Bionano Genomics, cat. no. 20249). The Bionano Genomics Irys System was used to linearised and visualised the labelled and stained DNA molecules by method of automated electrophoresis into Irys Chip nanochannels.

Bionano data analysis

The BNX files with Bionano molecules information, received from QMUL facility, were processed with Bionano's software IrysView Genomic Analysis Viewer, version 2.5.1.29842, with versions r5134 of

PipelineCL.py, r5122 of RefAligner.cpp, r5122 of Assembler.cpp, and r5146 of Hybrid Scaffold; all running on Microsoft Windows 7 64 bits with Python 2.7.8. IrysView was used for alignment of Bionano molecules over the consensus genome sequence and visualisation, following the guidelines in the Bionano document "IrysView® v2.5.1 Software Training Guide. Document Number: 30035 Document Revision: G, 2016"; the result of Molecule Quality Report (alignment over reference sequence) was interpreted according to the document "Guidelines for Interpreting the Bionano Molecule Quality Report. Document number30175, Rev A" and following advice from BioNano Technical Support.

The **Molecules-to-Reference Alignment** was performed with 3 iterations and these parameters:

```
 -nosplit 2 -BestRef 1 -biaswt 0 -Mfast 0 -FP 1.5 -FN 0.15 -sf 0.2 -sd 0.0 -A 5 -outlier
1e-3 -outlierMax 40 -endoutlier 1e-4 -S -1000 -sr 0.03 -se 0.2 -MaxSF 0.25 -MaxSE 0.5 -
resbias 4 64 -maxmem 64 -M 3 3 -minlen 150 -T 1e-7 -maxthreads 32 -hashgen 5 3 2.4 1.5
0.05 5.0 1 1 3 -hash -hashdelta 10 -hashoffset 1 -hashmaxmem 64 -insertThreads 4 -maptype
0 -PVres 2 -PVendoutlier -AlignRes 2.0 -rres 0.9 -resEstimate -ScanScaling 2 -RepeatMask
5 0.01 -RepeatRec 0.7 0.6 1.4 -maxEnd 50 -usecolor 1 -stdout -stderr
```

For Nt.BspQI the Map Rate of 74.6% (Molecules showing high similarity to reference as percentage of 'N Molecules') is at the upper limit of the acceptable range (60-80%). The statistics for labels present or absent, which were FP 0.53 per 100kb, FP 2.4%, and FN 20.9%, are also well under the limits stated in the Guidelines (which are FP (/100kbp) < 1.7; FP (%) < 15%; FN (%) < 21%). Despite the Map Rate for Nt.BbvCI was only 48.4% the relevant statistic indicators for the quality of mapping were well within the range established by Bionano, at FP 1.28/100kbp, FP 8.8%, and FN 18.4%. "FP (/100kb)" means density of molecule labels absent in the reference map (relative to reference labels); "FP (%)" means percentage of molecule labels absent in the reference map (relative to reference labels); "FN (%)" means percentage of reference labels absent in the aligned molecules (relative to reference labels); see Bionano's document number30175 for details.

Therefore, the Bionano data aligns well with the reference sequence, supporting the correctness of our NGS assembly.

The BNX files with Bionano molecules information, received from QMUL facility, were also processed with Bionano Solve Pipeline for constructing a ***de-novo* assembly and calling for Structural Variations**.

Solve pipeline was installed in a 64bits Ubuntu 16.04.1 system following guidelines in the document "30205-Guidelines-for-Running-Bionano-Solve-Pipeline-on-Command-Line.pdf". The actual version used was Solve3.4.1_09262019. Analysis was also performed following the guidelines in the aforementioned document. The genome assembly reported in this paper was provided as reference (used only to automatically calculate initial internal parameter of the pipeline, and later for Structural Variations call against the reference provided). For visualisation of the results we used MapOptics (https://doi.org/10.1093/bioinformatics/bty1013) specifically the version uploaded to the github repository (https://github.com/FadyMohareb/mapoptics) on 21st Nov 2018.

The command used to run Solve was:

```
python Solve/Pipeline/09262019/pipelineCL.py -T 240 -j 60 -N 4 -f 0.2 -i 5 -y -b all.bnx
-l '/media/WorkingHD/bbv' -t '/media/WorkingHD/Solve/RefAligner/8949.10020rel' -a
'/media/WorkingHD/Solve/RefAligner/8949.10020rel/optArguments_haplotype_irys.xml' -r
'/media/WorkingHD/reference.cmap'
```

Resolution of deletions called by Bionano Solve De-Novo Assembly and Structural Variant Analysis

The Bionano Solve Structural Variant Analysis based on alignment of contigs from a de-novo assembly of Bionano molecules over a reference sequence, had called 3 and 2 putative deletions for the data obtained with the enzyme Nt.BspQI and Nt.BbvCI respectively (data extracted from file `solve_output/contigs/exp_refineFinal1_sv/EXP_REFINEFINAL1.smap`):

Replicon	RefStartPos	RefEndPos	SVsize (estimated bp)	Confidence	Enzyme
Chromosome	5147150.0	5148103.0	537.4	0.90	Nt.BspQI
**Chromosome**	**5203317.5**	**5216661.0**	**5397.5**	**0.99**	**Nt.BspQI**
**Chromosome**	**5203317.5**	**5219278.0**	**5471.4**	**1.00**	**Nt.BspQI**
pSCL4	1344546.0	1348570.0	573.5	0.90	Nt.BspQI
**Chromosome**	**5199608.0**	**5221829.0**	**5416.7**	**0.99**	**Nt.BbvCI**
Chromosome	5746580.0	5747954.0	583.4	0.85	Nt.BbvCI

It must be noted that the positions are those of the labelling sites present in the reference sequence, they are not precise nucleotide positions since Bionano molecules do not have nucleotide information but only distances between labels. The SVsize, annotated by Solve, corresponds to the difference between the size of “RefEndPos-RefStartPos” and “QryEndPos-QryStartPos”. The two overlapping lines for the Chromosome with Nt.BspQI are due to the assembly of two independent contigs that seem to contain this deletion.

Despite the apparent high confidence assigned to all calls, detailed analysis of the alignment of Bionano molecules to the reference sequence showed very good mapping and did not provide a clear reason for the call of a deletion in these segments. Furthermore, the putative deletions on the regions 5.14Mb and 5.74 Mb of the chromosome and the putative deletion of pSCL4, are supported by only one enzyme, while the other enzyme supports the PacBio assembly. We therefore dismiss these calls.

Only the putative deletion between 5.20Mb and 5.21Mb of the chromosome is supported by the data obtained with both enzymes. Detailed analysis of the alignment revealed that one theoretical labelling site for each enzyme did not find a matching label in the molecules, for both enzymes, while all other labelling sites around are clearly identified in the molecules, indicating an apparent loss of only the sequence where those labelling sites are present (Fig S2 and S3).However, a detailed analysis with Bionano Access of an independent Solve assembly of Nt.BspQI data (kindly processed and provided by Bionano Technical Support) reveals the existence of molecules that fully support the DNA assembly (Fig S4).

A detail study of the DNA sequence at these positions reveals the two expected labelling sites inside a coding sequence (Fig S5). It is interesting to look at the GC Frame Plot in Fig S4, which changes dramatically at the left of the sites towards the 3’-end of the codding sequence (bear in mind that the labelling sites are on the bottom strand, which is also the coding strand); the change is, however, not indicative of a clear shift in the reading frame provoked by an error in the sequence. Also, a Blast search using the full gene coding sequence reveals full matches at 100% identity (without any difference or gap) when searching against each original PacBio assembly independently, the corrected-reads from each PacBio assembly, and

even a 45kb Illumina contig (NODE_3_length_44944_cov_92.486) contains the full coding sequence with no difference to the PacBio final assembly. Furthermore, a Blast search at NCBI revealed 100% identity without gaps or differences with all other high-quality genome sequences available for *S. clavuligerus*. The hits are not perfect with the Korean or Groningen assemblies, but both labelling sites are present. This gene ctg1_4289 has been annotated as encoding a "putative sensor and ATPase, component of G- protein-coupled receptor (GPCR) system" by RAST. antiSMASH did not annotate a putative function or product but it did annotate a Pfam HAMP domain, and according to InterPro database (http://www.ebi.ac.uk/interpro/entry/IPR003660) it is "an approximately 50-amino acid alpha-helical region present in Histidine kinases, Adenyl cyclases and Methyl-accepting proteins and Phosphatases. It is found in bacterial sensor and chemotaxis proteins and in eukaryotic histidine kinases. A blast search at NCBI with the encoded amino-acid sequence provides mostly *Streptomyces* proteins annotated as "HAMP domain-containing protein" and the HSPs cover all of the ctg1_4289 amino-acid sequence, with most variation towards the carboxi-terminus.

A more comprehensive sequence analysis was performed with the full stretch from position 5203317 to 5219279. Blast analysis using this sequence as query against databases of the original independent PacBio assemblies, the Illumina contigs and reads independently, and the corrected reads and the subreads independently, provided in all cases complete coverage of the sequence without any indication of a >5kb insertion. As a last test, this sequence aligns almost perfectly in two HSPs with the Korean assembly, and in three HSPs with a minor gap of 183 nt with the Groningen assembly.

Therefore, we are highly confident that the PacBio assembly published in this paper is correct. We have made multiple toes of investigation into this disagreement between the Bionano data and the PacBio assembly, and we deemed this call an artifact of the Bionano optical mapping assembly.

Figure S2. MapOptics view of the Bionano assembled contigs with Nt.BspQI (top) and Nt.BbvCI (bottom) at the conflict region. The strip immediately below the ruler corresponds to the reference chromosome sequence (as it does the position in the ruler); below it there are two strips (for Nt.BspQI) and one strip (for Nt.BbvCI) that represent the *de-novo* assembled Bionano contigs. It is interesting to notice the missing site (black vertical line in the reference strip just at the left of the 5214.75 kp mark) not present in any of the contigs covering this region, and the also missing site just at the right of the 5214.75 kb mark but only in one of the two Bionano contigs. It is also interesting to notice that in the Nt.BbvCI assembly there are an abundance of labels in this chromosomal region and that, in addition to the missing label at the right of the 5206.01 kb mark which was called as a deletion, there are some other missing labels (black lines between green ones) that have not been deemed reliable enough for the software to call a deletion.

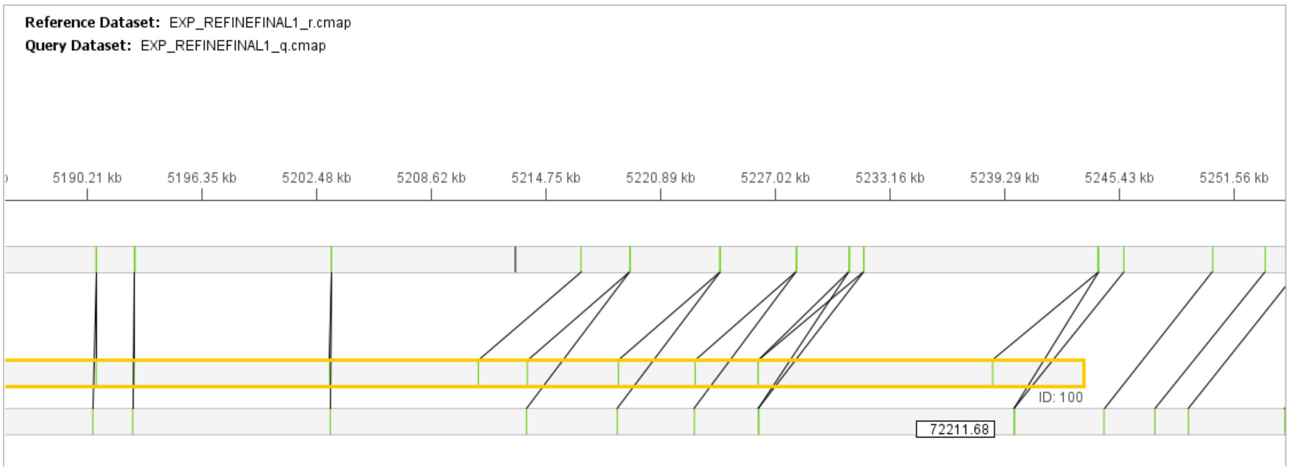

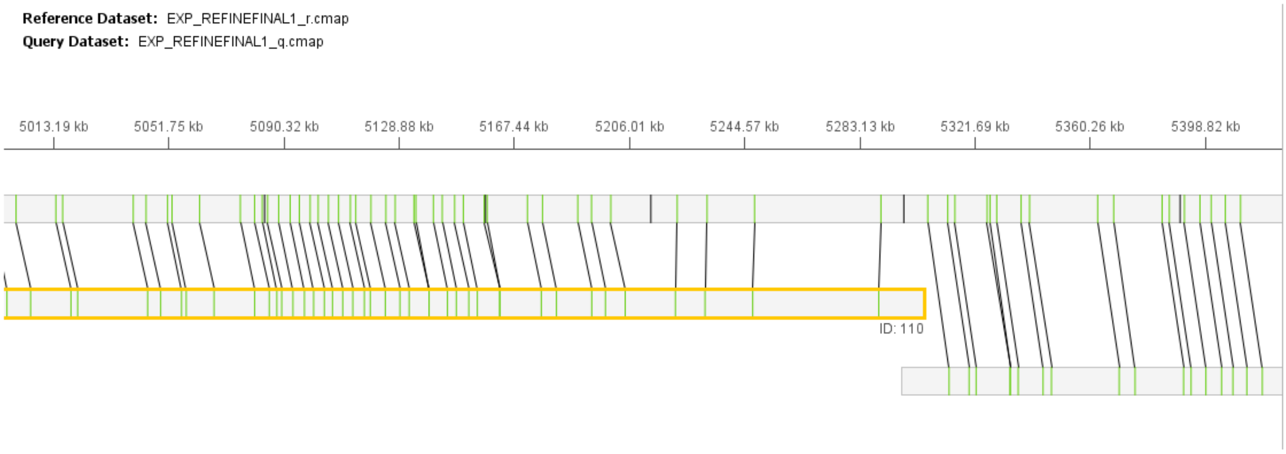

Figure S3 (next page). View of the alignment of Bionano molecules with Nt.BspQI (top) and Nt.BbvCI (bottom) nickases. For each enzyme, the view with "stretch matches" activated and deactivated (top and bottom respectively for each enzyme) are shown; this option does not change the actual result, it is just a visual aid to facilitate the understanding of the decisions taken by the software when assigning a detected label in the molecule to a predicted labelling site in the DNA sequence. It is noticeable in the "stretch_matches" views that there is not any Nt.BspQI molecule, and only one Nt.BbvCI, aligned with confidence threshold of 25 that contains a suitable label that could match the theoretical site present in the DNA sequence of the chromosome at position ~5.213Mb. It is also noticeable in the non-"stretch_matches" views that this region contains abundant labelling sites and that the limitations of the resolution in calculating distances between labels could impede the software finding an accurate match for each label on the reference.

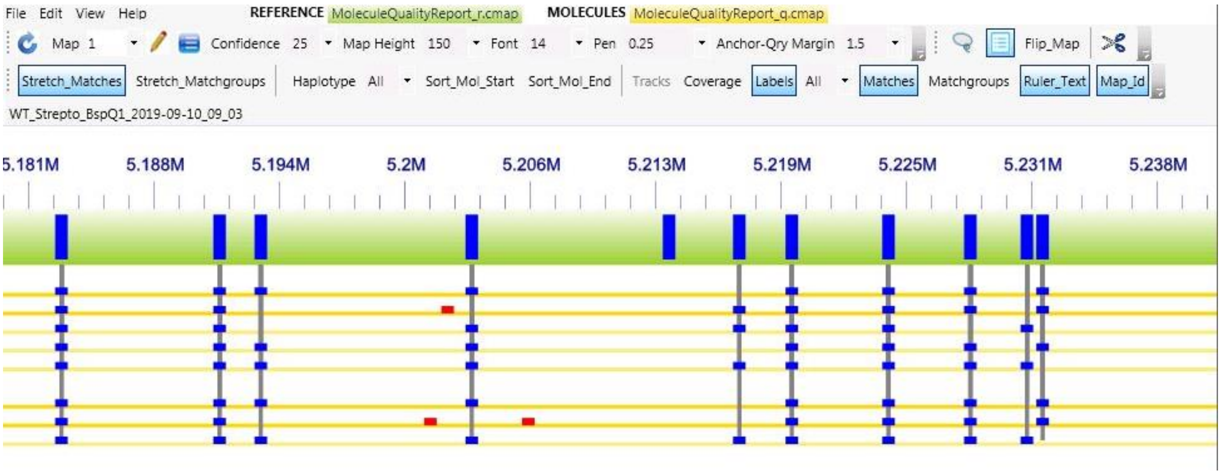
File Edit View Help
REFERENCE MoleculeQualityReport_r.cmap
MOLECULES MoleculeQualityReport_q.cmap
Map 1 Confidence 25 Map Height 150 Font 14 Pen 0.25 Anchor-Qry Margin 1.5
Flip_Map
Stretch_Matches Stretch_Matchgroups Haplotype All Sort_Mol_Start Sort_Mol_End Tracks Coverage Labels All Matches Matchgroups Ruler_Text Map_Id
WT_Strepto_BspQ1_2019-09-10_09_03
5.181M 5.188M 5.194M 5.2M 5.206M 5.213M 5.219M 5.225M 5.231M 5.238M

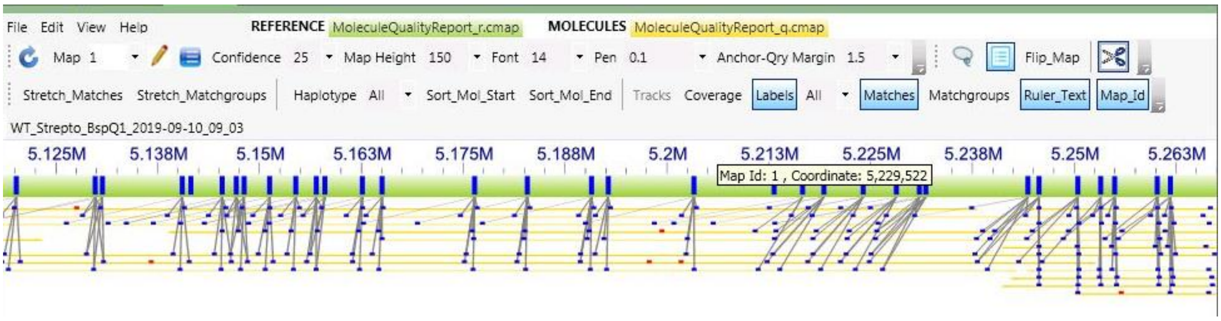
File Edit View Help
REFERENCE MoleculeQualityReport_r.cmap
MOLECULES MoleculeQualityReport_q.cmap
Map 1 Confidence 25 Map Height 150 Font 14 Pen 0.1 Anchor-Qry Margin 1.5
Flip_Map
Stretch_Matches Stretch_Matchgroups Haplotype All Sort_Mol_Start Sort_Mol_End Tracks Coverage Labels All Matches Matchgroups Ruler_Text Map_Id
WT_Strepto_BspQ1_2019-09-10_09_03
5.125M 5.138M 5.15M 5.163M 5.175M 5.188M 5.2M 5.213M 5.225M 5.238M 5.25M 5.263M
Map Id: 1 , Coordinate: 5,229,522

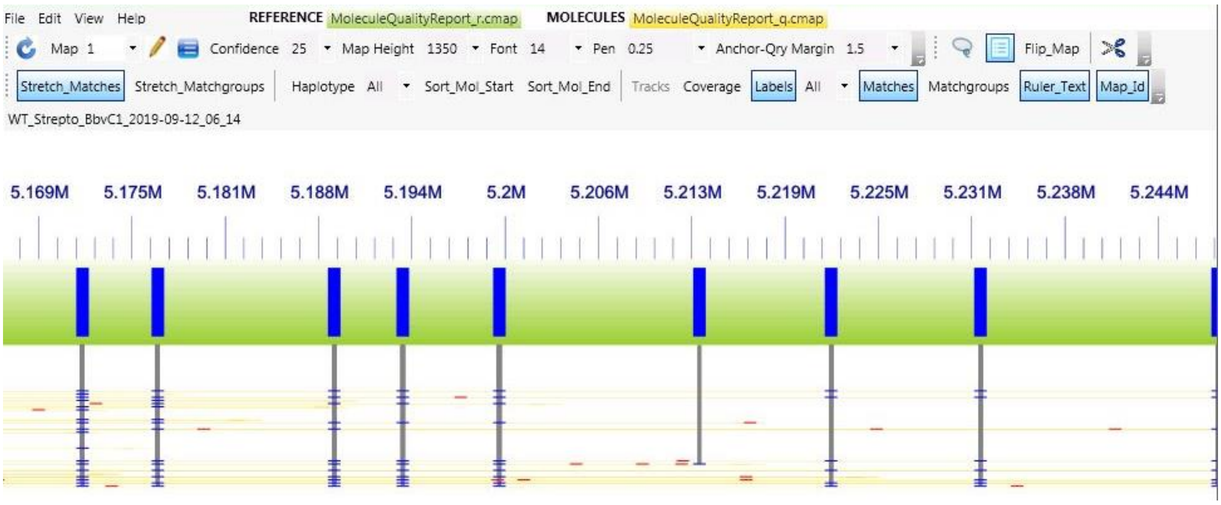
File Edit View Help
REFERENCE MoleculeQualityReport_r.cmap
MOLECULES MoleculeQualityReport_q.cmap
Map 1 Confidence 25 Map Height 1350 Font 14 Pen 0.25 Anchor-Qry Margin 1.5
Flip_Map
Stretch_Matches Stretch_Matchgroups Haplotype All Sort_Mol_Start Sort_Mol_End Tracks Coverage Labels All Matches Matchgroups Ruler_Text Map_Id
WT_Strepto_BbvC1_2019-09-12_06_14
5.169M 5.175M 5.181M 5.188M 5.194M 5.2M 5.206M 5.213M 5.219M 5.225M 5.231M 5.238M 5.244M

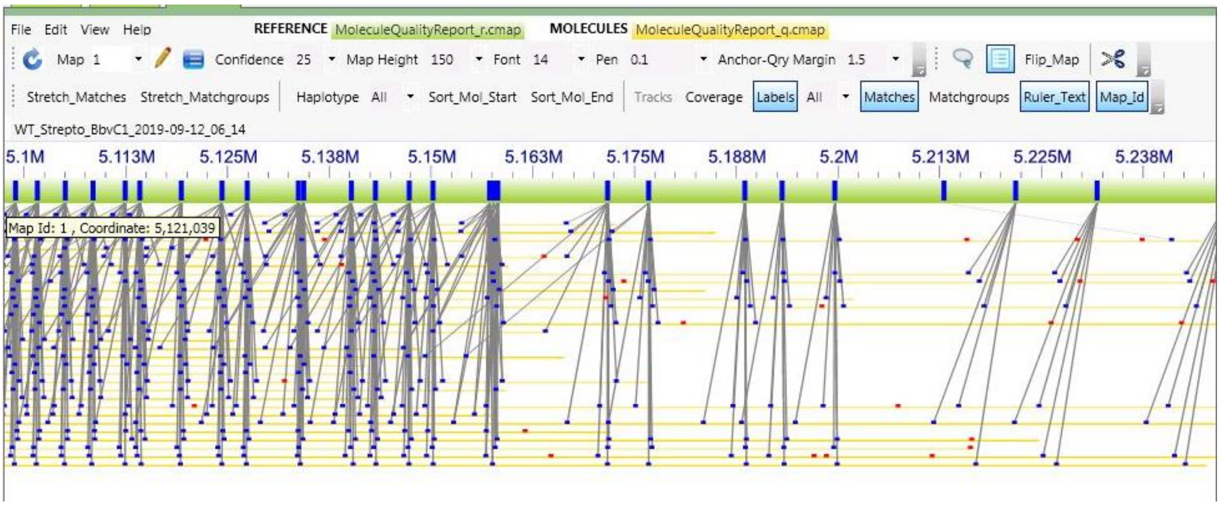
File Edit View Help
REFERENCE MoleculeQualityReport_r.cmap
MOLECULES MoleculeQualityReport_q.cmap
Map 1 Confidence 25 Map Height 150 Font 14 Pen 0.1 Anchor-Qry Margin 1.5
Flip_Map
Stretch_Matches Stretch_Matchgroups Haplotype All Sort_Mol_Start Sort_Mol_End Tracks Coverage Labels All Matches Matchgroups Ruler_Text Map_Id
WT_Strepto_BbvC1_2019-09-12_06_14
5.1M 5.113M 5.125M 5.138M 5.15M 5.163M 5.175M 5.188M 5.2M 5.213M 5.225M 5.238M
Map Id: 1 , Coordinate: 5,121,039

Figure S4. Molecules view of the Solve assembly of Nt.BspQI data. The top green segment represents the DNA assembly provided as reference. The two blue segments represent Bionano Solve contigs obtained after de novo assembly of Bionano molecules. The orange horizontal lines under the blue segments represent the Bionano molecules. The vertical bars represent the expected labelling sites (in the reference segment) and the consensus detected labelling sites (in the Bionano contigs); the actual labels detected in the molecules are depicted by spikes on top of the molecules (orange horizontal lines). The red boxes highlight the segment with the predicted insertion in the DNA sequence (deletion in the Bionano assembly); the orange arrows indicate the predicted labelling sites in the DNA reference (PacBio assembly) that are also detected in the top molecule (the orange horizontal line) but that the software has failed to annotate as a match and, probably due to the low representability of molecules with the full set of labels, the software has created two contigs missing that consensus label.

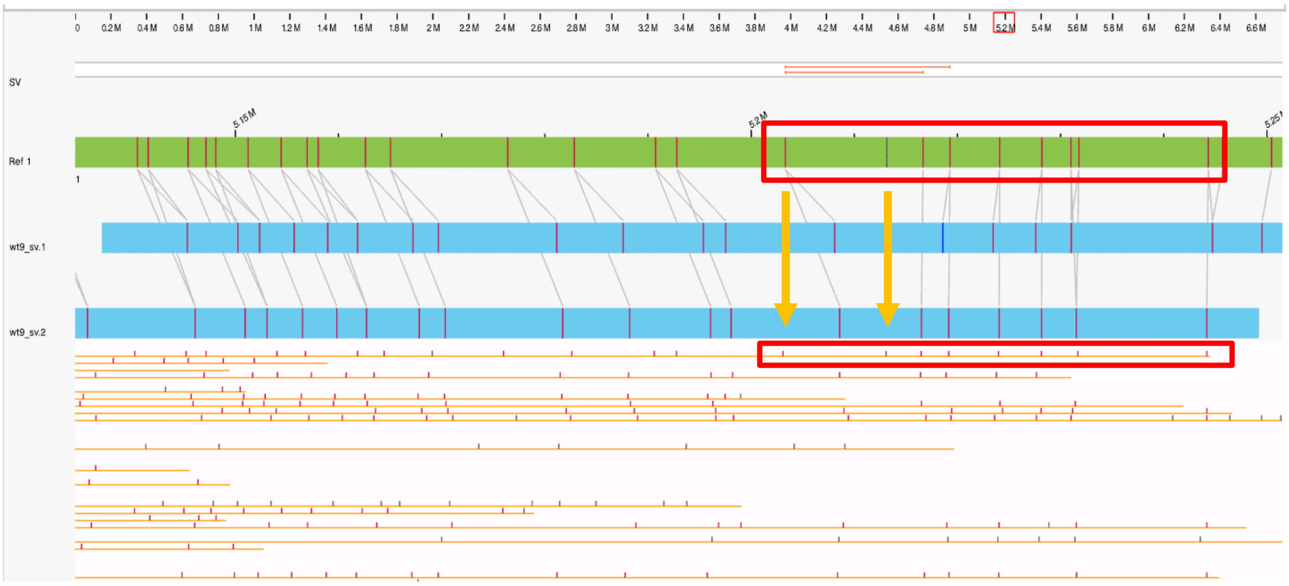

Figure S5. Screenshot of Artemis showing the genetic structure of the coding sequence containing the expected labelling sites for Nt.BspQI (GCTCTTC) and Nt.BbvCI (CCTCAGC) which are missing in the Bionano molecules.

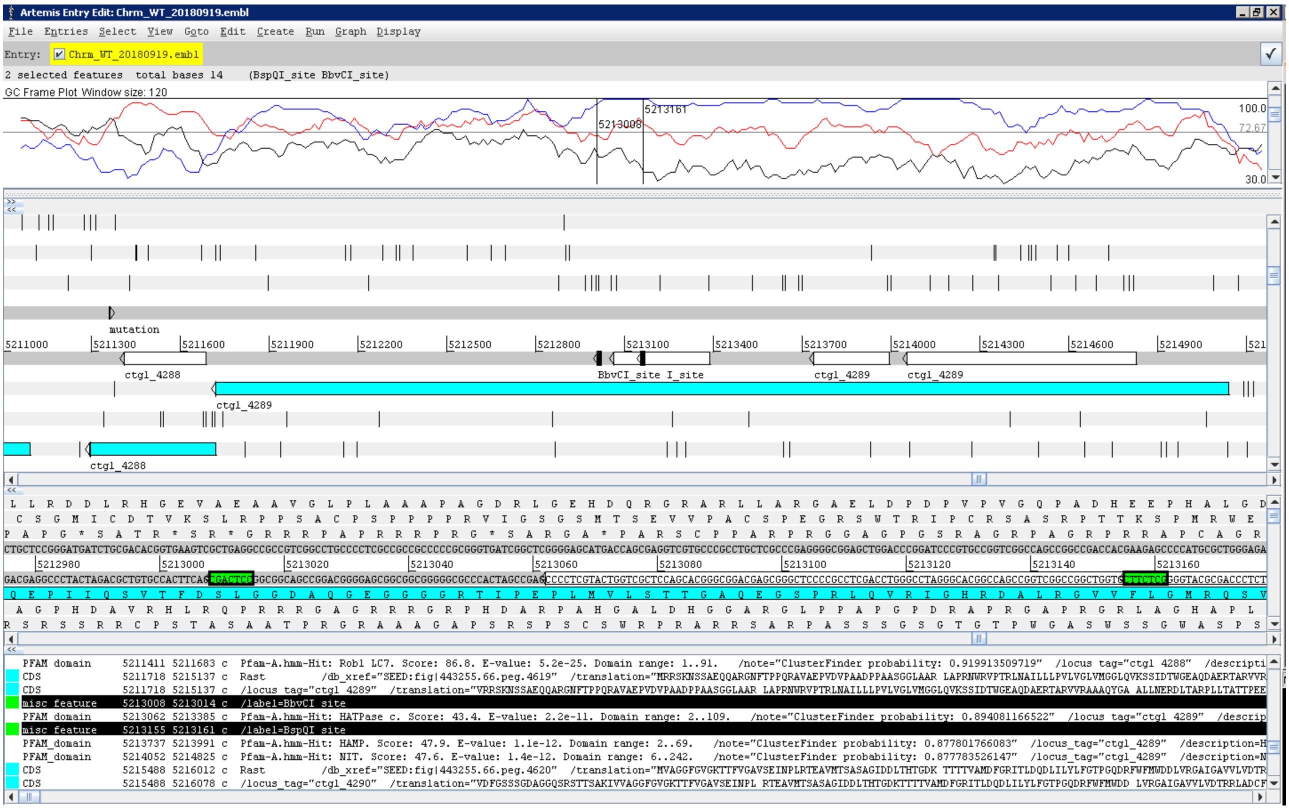

ASSESSMENT OF PRESENCE OF pSCL3 IN TYPE-STRAIN DEPOSITS

What follows is an analysis of the Illumina data obtained for the ATCC 27064 type strain-Leon isolate and the type strain deposits obtained directly from the culture collections. The trimmed Illumina reads were mapped to the reference assembly (using BWA as previously reported), including the known sequence of pSCL3, and the resulting BAM files were analysed with Qualimap software (note that pSCL1 is too small to be distinguishable in these images). It is quickly noticeable that while in the mapping of data from the Leon-isolate there are only a few reads mapped to pSCL3, probably due to high similarity with some other sequence in the genome, this replicon is fully covered by mapped reads for the data from all other five type deposits analysed.

ATCC 27064 Leon-isolate

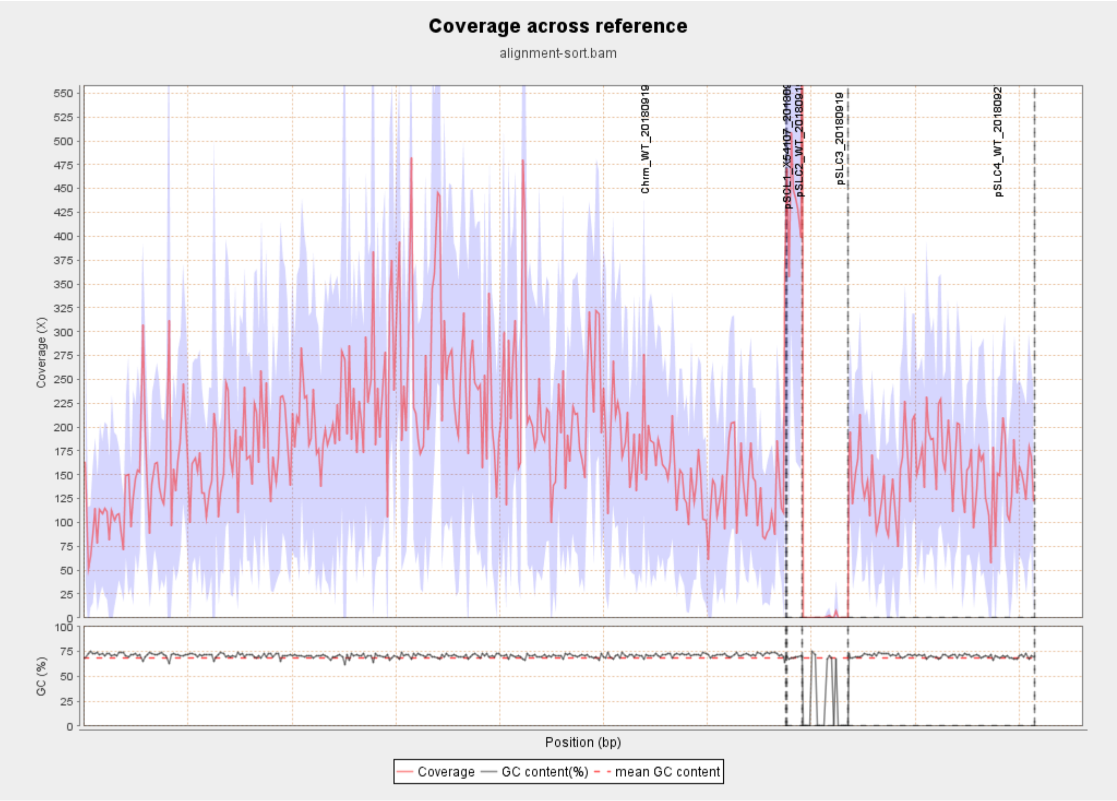

ATCC 27064 culture collection deposit

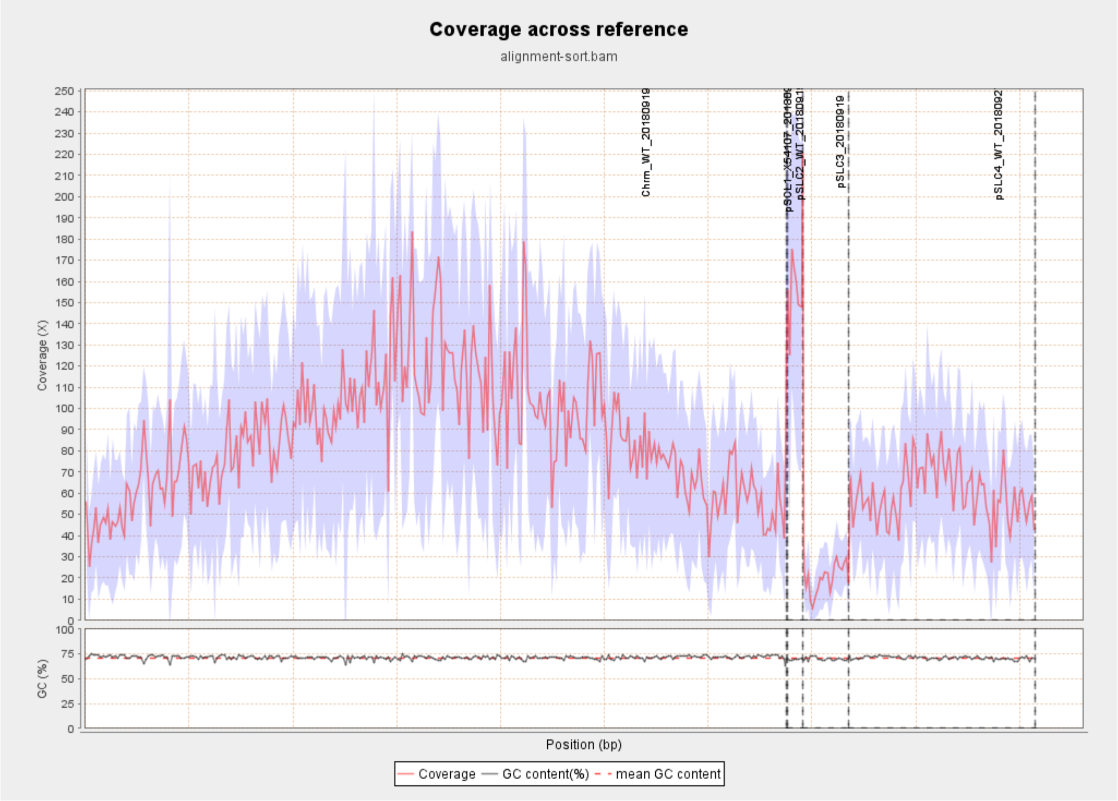

DSM 738 culture collection deposit

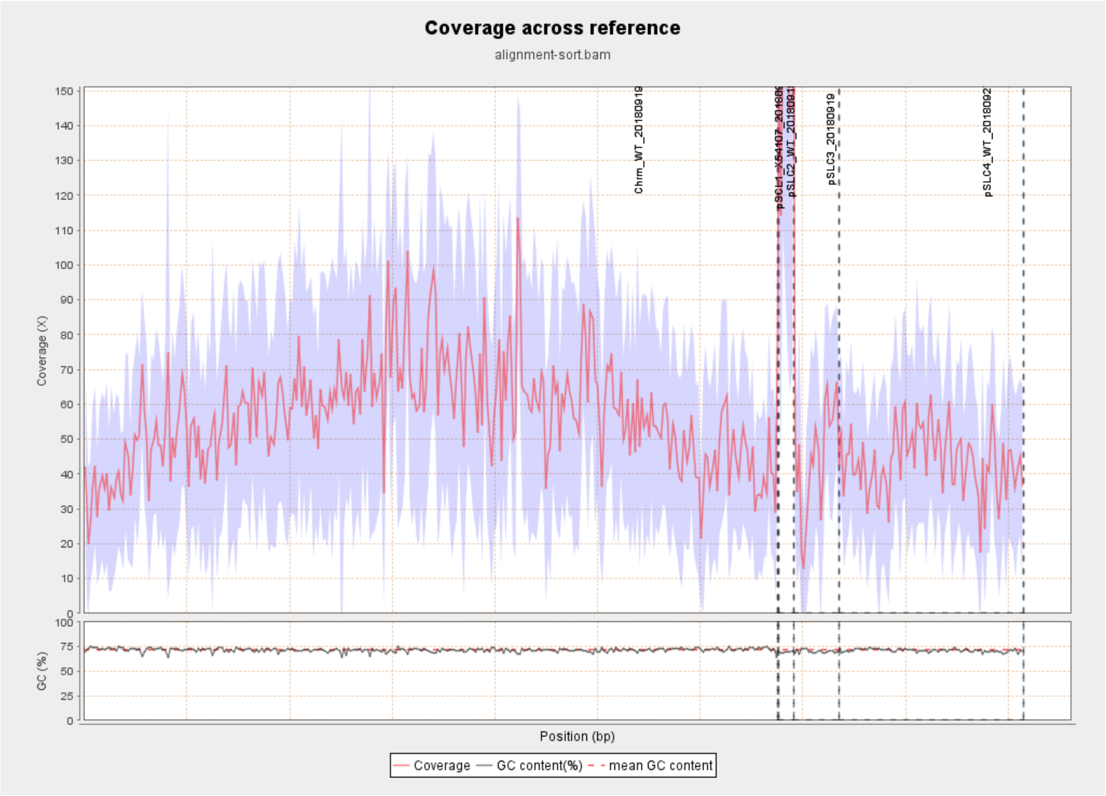

NRRL 3585 culture collection deposit

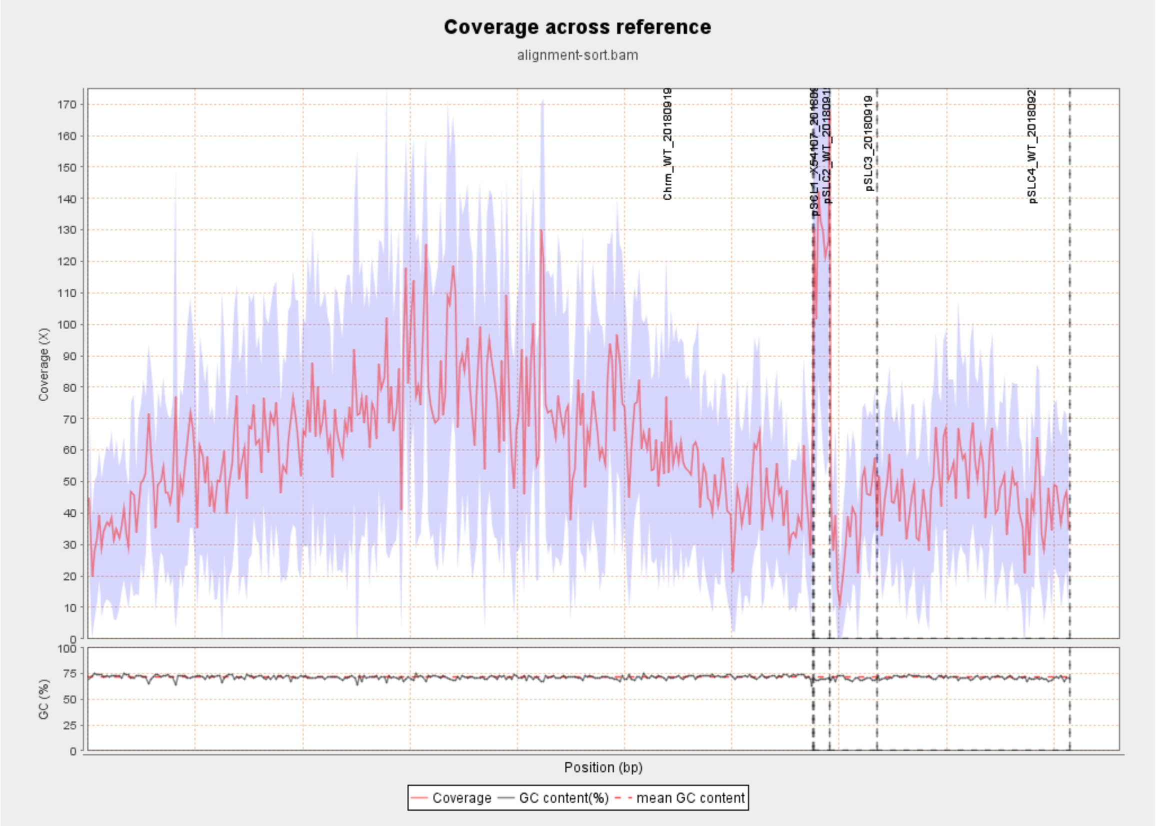

NCIMB 12785 culture collection deposit

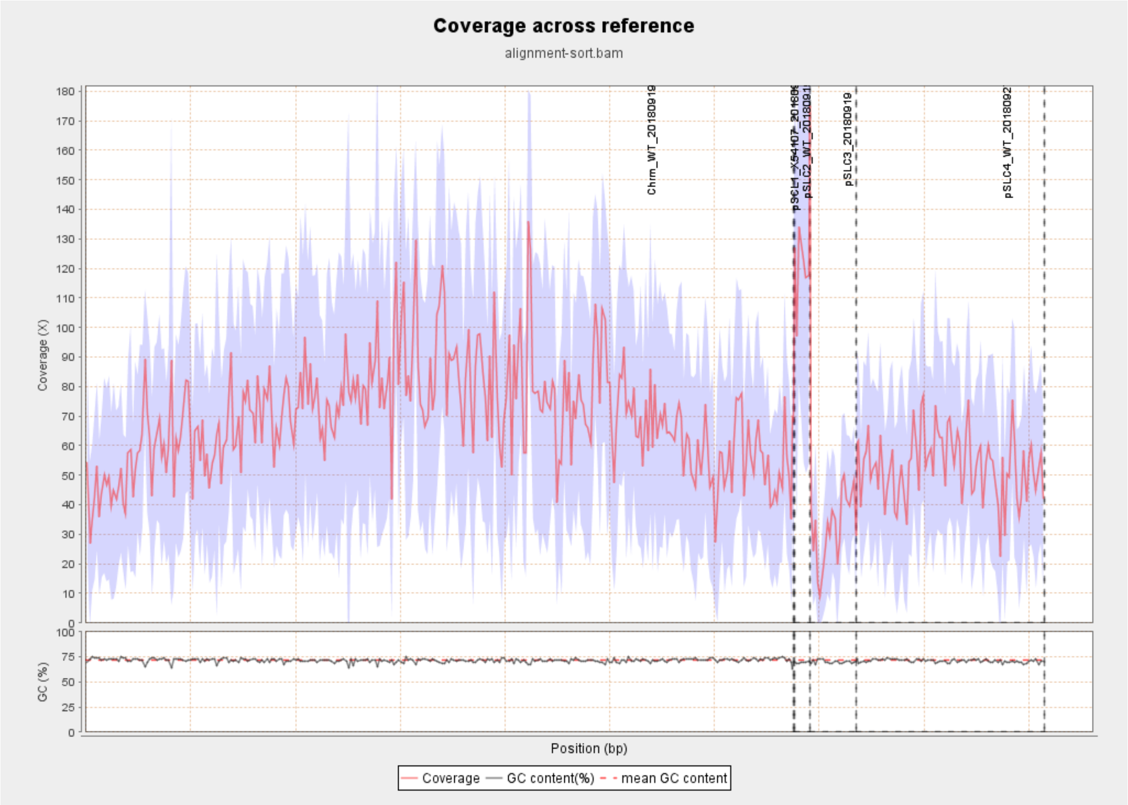

NCIMB 14335 culture collection deposit

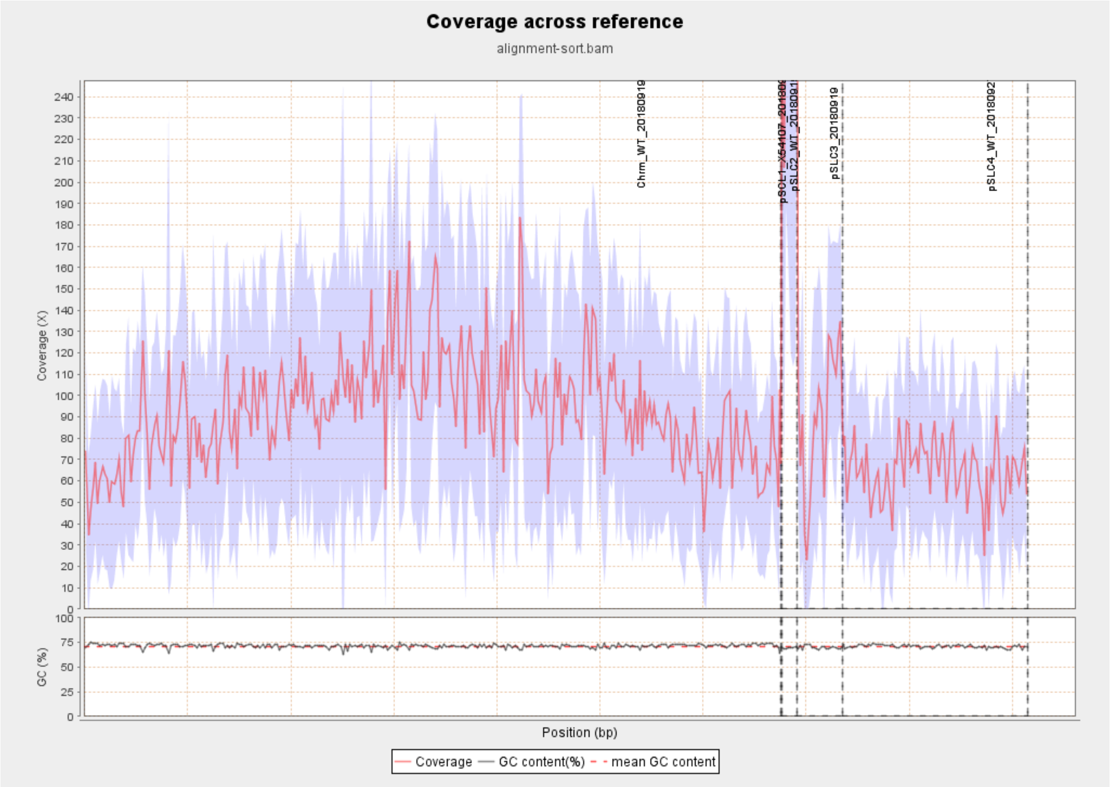

Table S6. Coverage of each replicon by Illumina reads and how it compares with chromosome (figures from Qualimap report).

**Isolate**	**Leon**	**ATCC27064**	**NRRL3585**	**DSM738**	**NCIMB12785**	**NCIMB14335**
Mean Coverage	176.9391	79.6936	59.3020	55.5214	65.5365	90.2125
Standard Deviation Coverage	155.3577	56.4119	41.0598	37.5891	43.3969	64.4059
Mean Mapping Quality	207.5600	212.8900	211.8100	214.1300	212.7600	213.6500
**Mean coverage per replicon**						
Chrm_WT_20180919	189.1144	87.0014	62.5583	57.1428	69.7308	93.1328
pSCL1_X54107_20180919	409.4844	127.2391	69.6293	83.1081	68.8806	68.1374
pSCL2_WT_20180919	438.3335	154.7871	126.5857	151.9817	120.8499	262.2835
pSCL4_WT_20180927	151.6521	60.4297	46.9289	43.8545	53.2879	65.2323
pSCL3_NZ_CM001018.1	0.0689	19.9892	36.9153	44.8489	32.6065	89.4706
**Coverage times chromosome**						
Chrm_WT_20180919	1.0000	1.0000	1.0000	1.0000	1.0000	1.0000
pSCL1_X54107_20180919	2.1653	1.4625	1.1130	1.4544	0.9878	0.7316
pSCL2_WT_20180919	2.3178	1.7791	2.0235	2.6597	1.7331	2.8162
pSCL4_WT_20180927	0.8019	0.6946	0.7502	0.7675	0.7642	0.7004
pSCL3_NZ_CM001018.1	0.0004	0.2298	0.5901	0.7849	0.4676	0.9607
**Standard deviation coverage per replicon**						
Chrm_WT_20180919	153.7859	57.3747	41.6595	35.8030	44.2101	60.8462
pSCL1_X54107_20180919	492.7671	115.8106	63.5678	79.4873	62.8241	67.0443
pSCL2_WT_20180919	255.9858	76.4477	65.2766	76.1768	61.9622	133.4119
pSCL4_WT_20180927	109.9354	35.8577	28.8932	26.1634	32.2816	40.7680
pSCL3_NZ_CM001018.1	1.5807	13.6738	25.2423	29.8084	22.3847	61.1916

CURING OF pSCL4

PCR test of pSCL4-cured candidate strains

The following oligonucleotides were used for PCR amplification (from 5’ to 3’): JP284 TGGACTCGCTACTGCATCTG; JP291 GTAGCCGTCCTTGGTGATGT; JP293 GCCGTCCTTGGTGATGTAGT; JP294 GCACCTGGGATGTACAGGAC; JP295 GTATCCAGCCAGTCCAGGAG; JP297 ATGAGCGTATCCAGCCAGTC; JP299 ATCACCTCCTTCCGTTGTTG; JP401 GACCCCGAAGAAGTTGACCT. Primer pairs used were JP401/JP284, JP297/JP299, JP291/JP294, JP293/JP294, JP295/JP299.

Analysis of Illumina whole-genome sequencing of pSCL4-cured clones

Table S7. Coverage of each replicon by Illumina reads and how it compares with chromosome (figures from Qualimap report).

**Isolate**	**Leon (parental)**	**BW0216**	**BW0217**	**BW0218**	**BW0219**	**BW0220**
Mean Coverage	176.9391	69.3759	30.1701	85.5109	76.7832	66.5763
Standard Deviation Coverage	155.3577	63.2305	30.0936	195.3462	71.2394	115.7948
Mean Mapping Quality	207.5600	158.08	159.17	160.85	161.47	158.31
**Mean coverage per replicon**						
Chrm_WT_20180919	189.1144	90.6385	39.0099	111.5352	99.3397	87.4949
pSCL1_X54107_20180919	409.4844	168.1029	88.8633	196.758	302.2681	166.2561
pSCL2_WT_20180919	438.3335	139.6732	77.8032	181.2468	189.4599	110.5904
pSCL3_NZ_CM001018.1	151.6521	0.3076	0.1171	0.352	0.3217	0.2435
pSCL4_WT_20180927	0.0689	0.0519	0.033	0.0714	0.072	0.0498
**Coverage times chromosome**						
Chrm_WT_20180919	1.0000	1.0000	1.0000	1.0000	1.0000	1.0000
pSCL1_X54107_20180919	2.1653	1.8547	2.2780	1.7641	3.0428	1.9002
pSCL2_WT_20180919	2.3178	1.5410	1.9944	1.6250	1.9072	1.2640
pSCL3_NZ_CM001018.1	0.8019	0.0034	0.0030	0.0036	0.0032	0.0028
pSCL4_WT_20180927	0.0004	0.0006	0.0008	0.0006	0.0007	0.0006
**Standard deviation coverage per replicon**						
Chrm_WT_20180919	153.7859	55.7273	27.2629	219.5146	61.0974	126.8952
pSCL1_X54107_20180919	492.7671	154.5085	85.9898	181.4763	271.159	163.1953
pSCL2_WT_20180919	255.9858	67.5534	40.2278	87.2812	92.4584	53.8525
pSCL3_NZ_CM001018.1	109.9354	3.6557	1.4341	4.0699	3.8864	2.7477
pSCL4_WT_20180927	1.5807	2.0029	1.2404	2.7381	2.7583	1.8965

Analysis of BW0216

The coverage map produced by Qualimap already shows a lack of a significant number of reads mapping to pSCL4 (as well as to pSCL3, as expected for the parental strain) and also a lack of mapping to the ends of the chromosome. Further analysis has revealed that the chromosome has circularised losing ~100kb of each end while psCL1 and pSCL2 are intact.

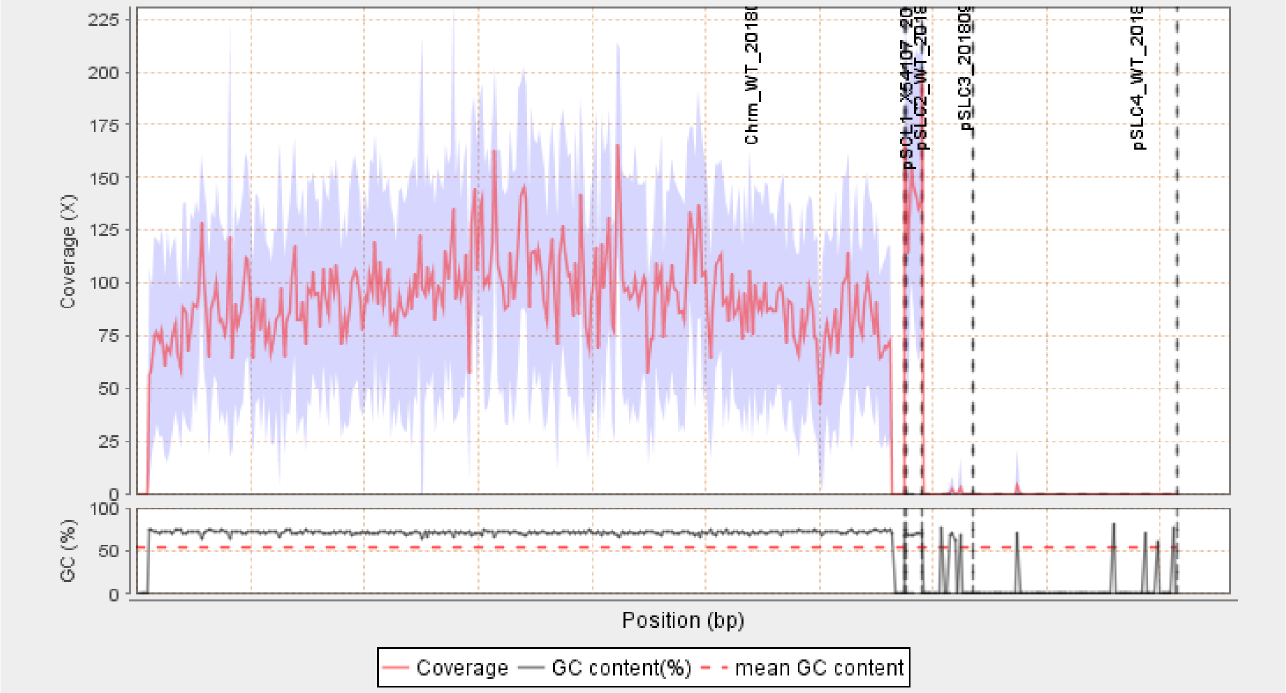

Analysis in GAP5 shows that the high-quality mapping starts at around position 98990 of the chromosomal left end (these are alignment coordinates, not precise chromosome positions):

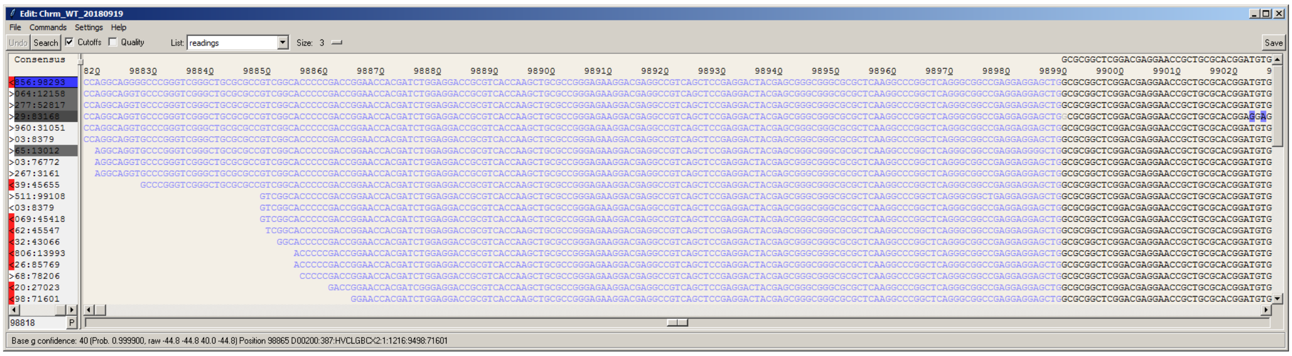

and ends ataround position 6632681 of the chromosome right end (6632948 of the alignment):

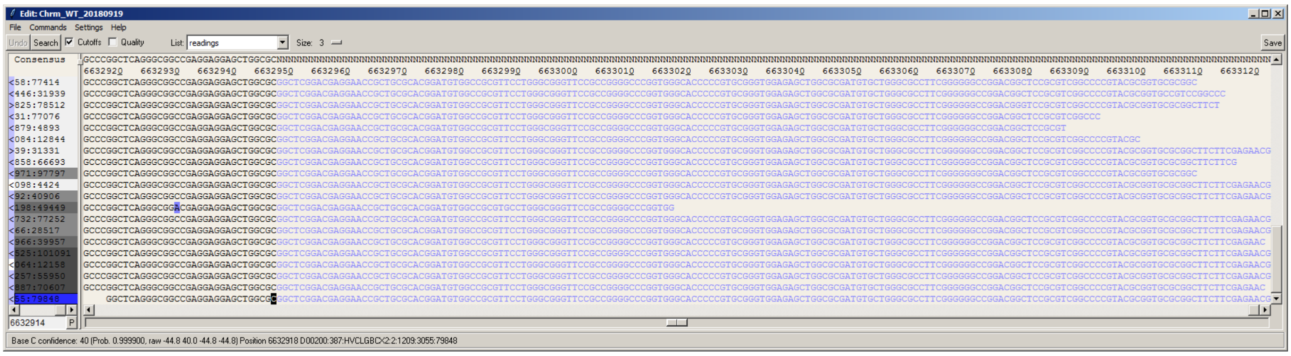

Another clear observation is the perfect alignment of the remaining of the reads that stops matching the reference sequence. This clearly indicates that the chromosome sequence of this pSCL4-mutant ends at those points and continues somewhere else. To identify the new continuation regions, we perfomed a blast with the highlighted reads (blue in the screenshots):

[subject id: “WT_Chrm” in all cases]

query id	% identity	alignment length	mismatches	gap opens	q. start	q. end	s. start	s. end
A1_1_D00200:387:HVCLGBCX2:1:1202:14856:98293	100.00	251	0	0	1	251	6632210	6632460
A1_1_D00200:387:HVCLGBCX2:2:1209:3055:79848	100.00	225	0	0	1	225	99214	98990
A1_1_D00200:387:HVCLGBCX2:2:1209:3055:79848	93.48	46	2	1	206	251	6632681	6632637
A1_2_D00200:387:HVCLGBCX2:1:1202:14856:98293	98.25	228	3	1	22	249	6632681	6632455
A1_2_D00200:387:HVCLGBCX2:1:1202:14856:98293	100.00	41	0	0	1	41	99030	98990
A1_2_D00200:387:HVCLGBCX2:2:1209:3055:79848	100.00	247	0	0	1	247	6632403	6632649

A1_1 means “clone A”, “gDNA sample 1”, “paired read 1”; A1_2 means “clone A”, “gDNA sample 1”, “paired read 2”

Alignments as example:

```
Query= A1_1_D00200:387:HVCLGBCX2:2:1209:3055:79848

Length=251

>lcl|WT_Chrm
Length=6748580

 Score =  416 bits (225),  Expect = 8e-117
 Identities = 225/225 (100%), Gaps = 0/225 (0%)
 Strand=Plus/Minus

Query  1      GTCCGGCCCGGCCCGGCGACCCGCAGCAGCCACGCCGTGCGCCCGCCGTTCTCGAAGAAG  60
              ||||||||||||||||||||||||||||||||||||||||||||||||||||||||||||
Sbjct  99214  GTCCGGCCCGGCCCGGCGACCCGCAGCAGCCACGCCGTGCGCCCGCCGTTCTCGAAGAAG  99155

Query  61     CCGCGCACCGCGTACGGGGCCGACGCGGAGCCGTCCGGCCCCCCGAAGGCGCCCAGCACA  120
              ||||||||||||||||||||||||||||||||||||||||||||||||||||||||||||
Sbjct  99154  CCGCGCACCGCGTACGGGGCCGACGCGGAGCCGTCCGGCCCCCCGAAGGCGCCCAGCACA  99095

Query  121    TCGCGCCAGCTCTCCACCCGCACGGGGGTGCCCACCGGGCCCCGGCGGAACCCGCCCAGG  180
              ||||||||||||||||||||||||||||||||||||||||||||||||||||||||||||
Sbjct  99094  TCGCGCCAGCTCTCCACCCGCACGGGGGTGCCCACCGGGCCCCGGCGGAACCCGCCCAGG  99035

Query  181    AACGCGGCCACATCCGTGCGCAGCGGTTCCTCGTCCGAGCCGCGC  225
              |||||||||||||||||||||||||||||||||||||||||||||
Sbjct  99034  AACGCGGCCACATCCGTGCGCAGCGGTTCCTCGTCCGAGCCGCGC  98990
```

```
 Score = 67.6 bits (36),  Expect = 9e-012
 Identities = 43/46 (93%), Gaps = 1/46 (2%)
 Strand=Plus/Minus

Query  206      GTTCCTCGTCCGAGCCGCGCCAGCTCCTCCTCGGCCGCCCTGAGCC  251
                |||||||| |||| | ||||||||||||||||||||||||||||||
Sbjct  6632681  GTTCCTCG-CCGACCTGCGCCAGCTCCTCCTCGGCCGCCCTGAGCC  6632637


=========================================================================================
Query= A1_2_D00200:387:HVCLGBCX2:1:1202:14856:98293

Length=249
                                                                      Score     E

>lcl|WT_Chrm
Length=6748580

 Score =  398 bits (215),  Expect = 3e-111
 Identities = 224/228 (98%), Gaps = 1/228 (0%)
 Strand=Plus/Minus

Query  22       GTTCCTCGTCCGAGCCGCGCCAGCTCCTCCTCGGCCGCCCTGAGCCGGGCCTTGAGCGCG  81
                |||||||| |||| | ||||||||||||||||||||||||||||||||||||||||||||
Sbjct  6632681  GTTCCTCG-CCGACCTGCGCCAGCTCCTCCTCGGCCGCCCTGAGCCGGGCCTTGAGCGCG  6632623

Query  82       CCCGCCCGCTCGTAGTCCTCGGAGCTGACGGCCTCGTCCTTCTCCCGGCGCAGCTTGGTG  141
                ||||||||||||||||||||||||||||||||||||||||||||||||||||||||||||
Sbjct  6632622  CCCGCCCGCTCGTAGTCCTCGGAGCTGACGGCCTCGTCCTTCTCCCGGCGCAGCTTGGTG  6632563

Query  142      ACGCGGTCCTCCAGATCGTGGTTCCGGTCGGGGGTGCCGACGGCGCGCAGCCCGACCCGG  201
                ||||||||||||||||||||||||||||||||||||||||||||||||||||||||||||
Sbjct  6632562  ACGCGGTCCTCCAGATCGTGGTTCCGGTCGGGGGTGCCGACGGCGCGCAGCCCGACCCGG  6632503

Query  202      GCCCCTGCCTGGTCCATCAGGTCGATGGCCTTGTCCGGGAGGAAGCGG  249
                || |||||||||||||||||||||||||||||||||||||||||||||
Sbjct  6632502  GCACCTGCCTGGTCCATCAGGTCGATGGCCTTGTCCGGGAGGAAGCGG  6632455


 Score = 76.8 bits (41),  Expect = 2e-014
 Identities = 41/41 (100%), Gaps = 0/41 (0%)
 Strand=Plus/Minus

Query  1      CGGCCACATCCGTGCGCAGCGGTTCCTCGTCCGAGCCGCGC  41
              |||||||||||||||||||||||||||||||||||||||||
Sbjct  99030  CGGCCACATCCGTGCGCAGCGGTTCCTCGTCCGAGCCGCGC  98990
```

This indicates that there is contiguity between position 98990 and 6632681.

Taking this into account, we took the chromosomal sequence 98990..100000 and used it to Blast all contigs, obtaining a single hit for contig NODE_89_length_14998_cov_34.2776. We then used the full sequence of this contig to blast the full genome assembly used as reference and obtain these only two HSPs (query is NODE_89_length_14998_cov_34.2776, subject is Chrm_WT_20180919):

% identity	alignment length	mismatches	gap opens	q. start	q. end	s. start	s. end
100.00	10257	0	0	4742	14998	98990	109246
99.94	4761	2	1	1	4761	6627922	6632681

This matches with the alignment of contigs in GAP5:

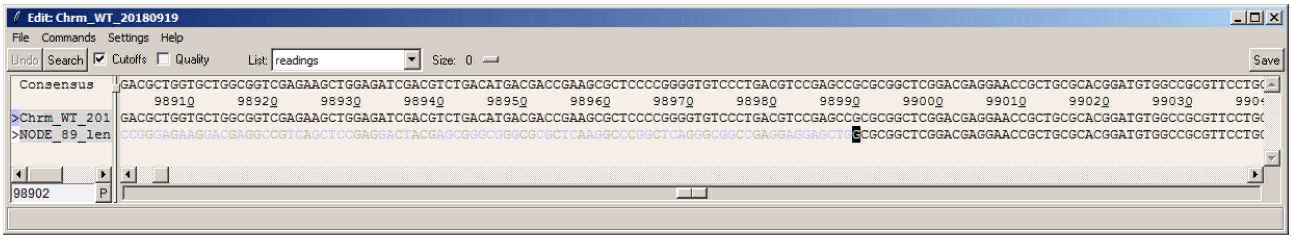

A view of the contig HSPs arrangement in Artemis illustrates clearer the chromosome circularisation:

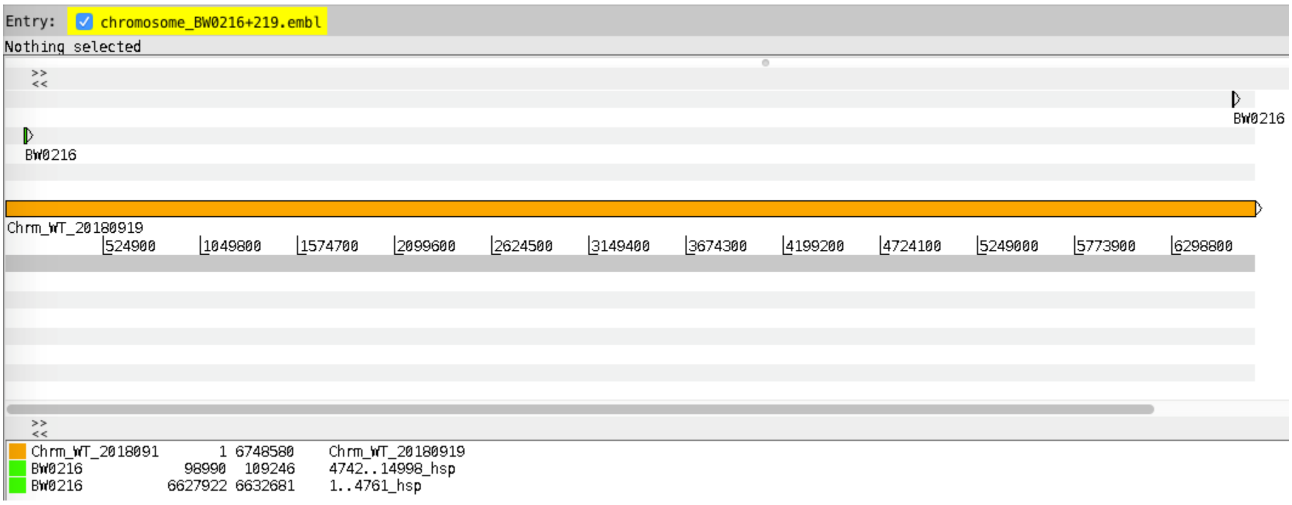

Regarding pSCL1, we haven't found any evidence of circularisation, neither in the reads or contigs alignments.

Regarding pSCL2, the analysis clearly indicates that the plasmid has been maintained in its complete original linear form.

Analysis of BW0217

The coverage map produced by Qualimap already shows a lack of a significant number of reads mapping to pSCL4 (as well as to pSCL3, as expected for the parental strain) and also a lack of mapping to the ends of the chromosome. pSCL1 and pSCL2 are intact.

The chromosome shows a very complicated to explain mapping. The Qualimap image shows a higher coverage at the left end, followed by a drastic reduction and then a high coverage again for the rest of the chromosome until being almost nil at the right end, indicating the loss of part of this terminus.

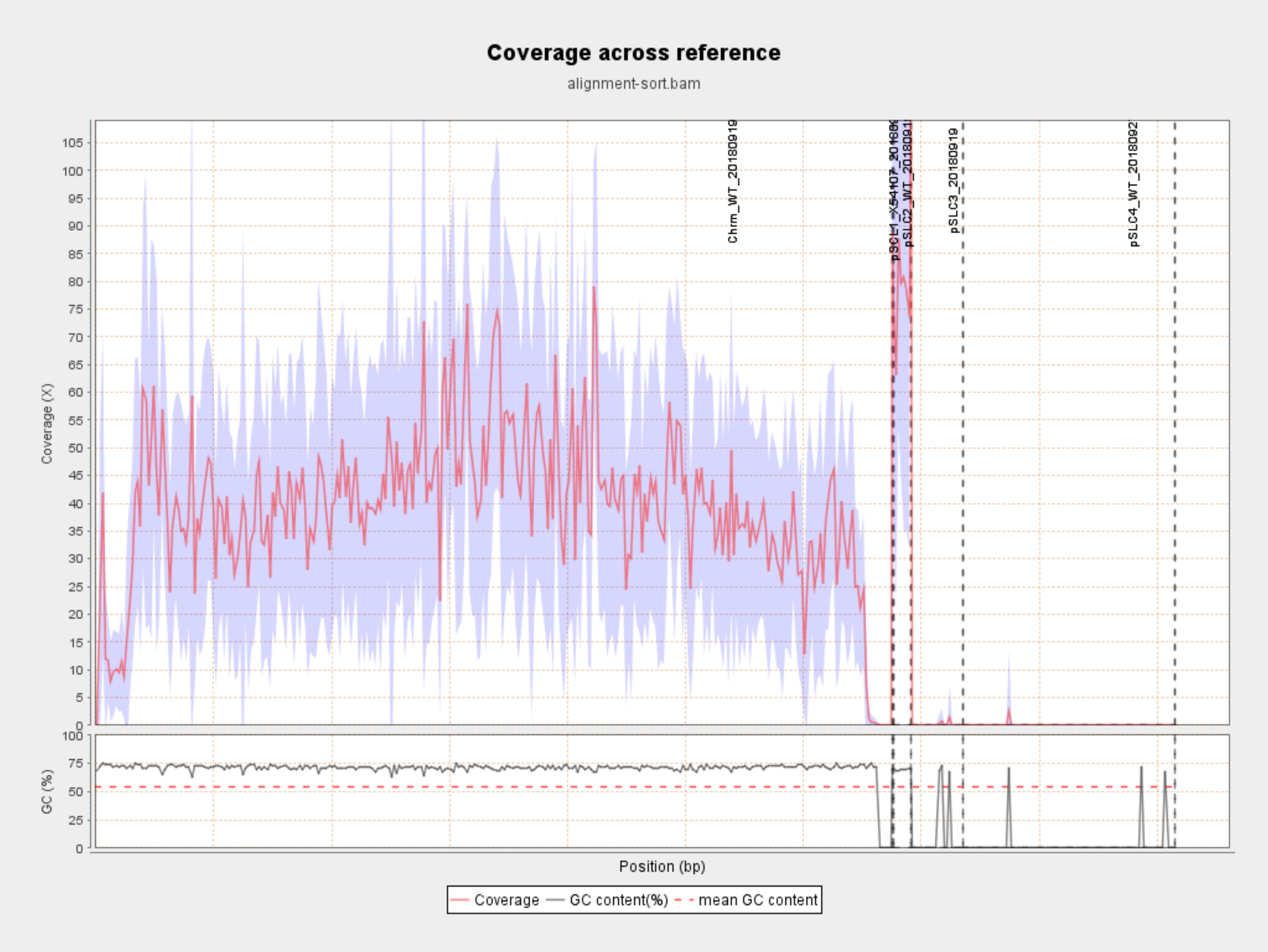

A detail analysis in GAP5 reveals the precise locations. Unfortunately, neither the reads nor contigs mapping reveal any clear arrangement of the chromosome. Curiously, this is the region that carries a large type I polyketide synthase (PKS) biosynthetic gene cluster, with the PKS genes spanning from position 13614 to 74762.

From about 70 kb to 270 kb there is a sudden change and reduction in coverage:

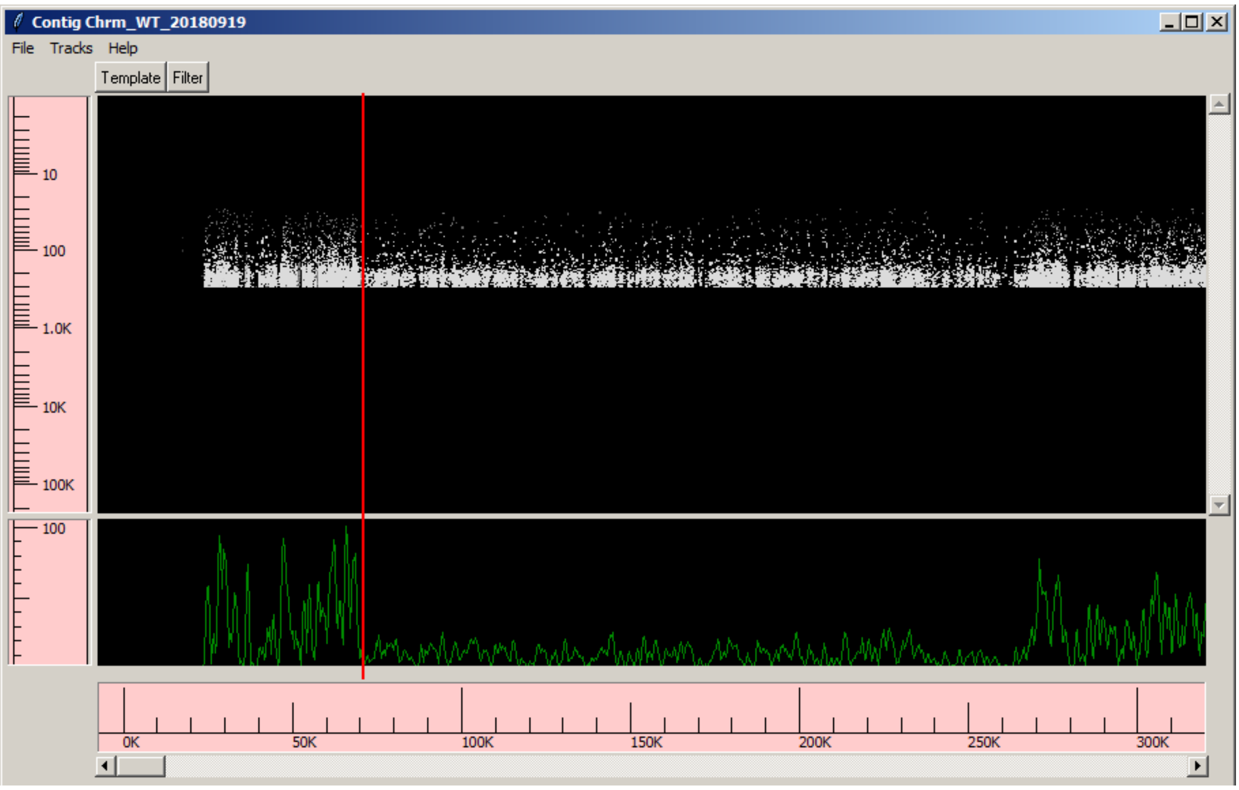

At the right end the good and even coverage ends at about 6530 kb:

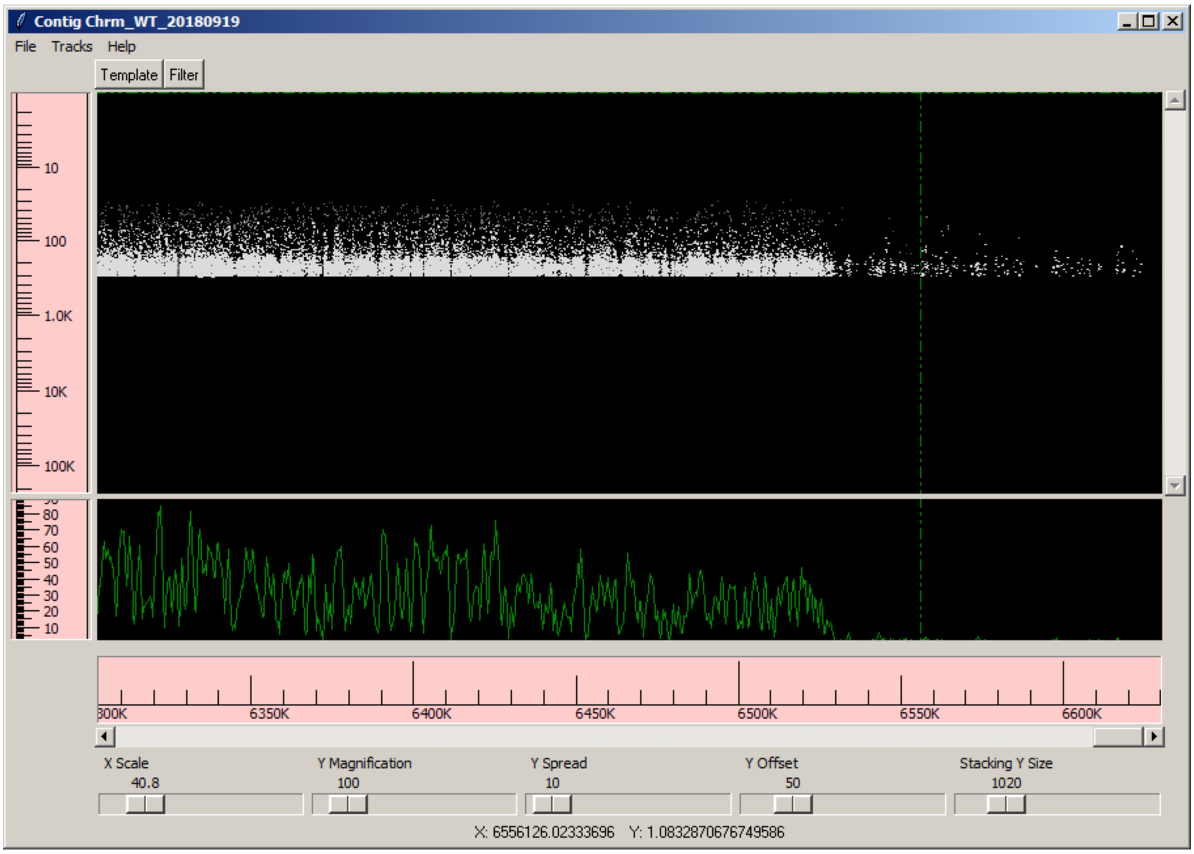

Regarding pSCL2, the same observations as in clone BW0216 apply.

Analysis of BW0218

The coverage map produced by Qualimap already shows a lack of a significant number of reads mapping to pSCL4 (as well as to pSCL3, as expected for the parental strain). In this mutant, however, there is a peak of coverage at the left end and significant coverage at the right.

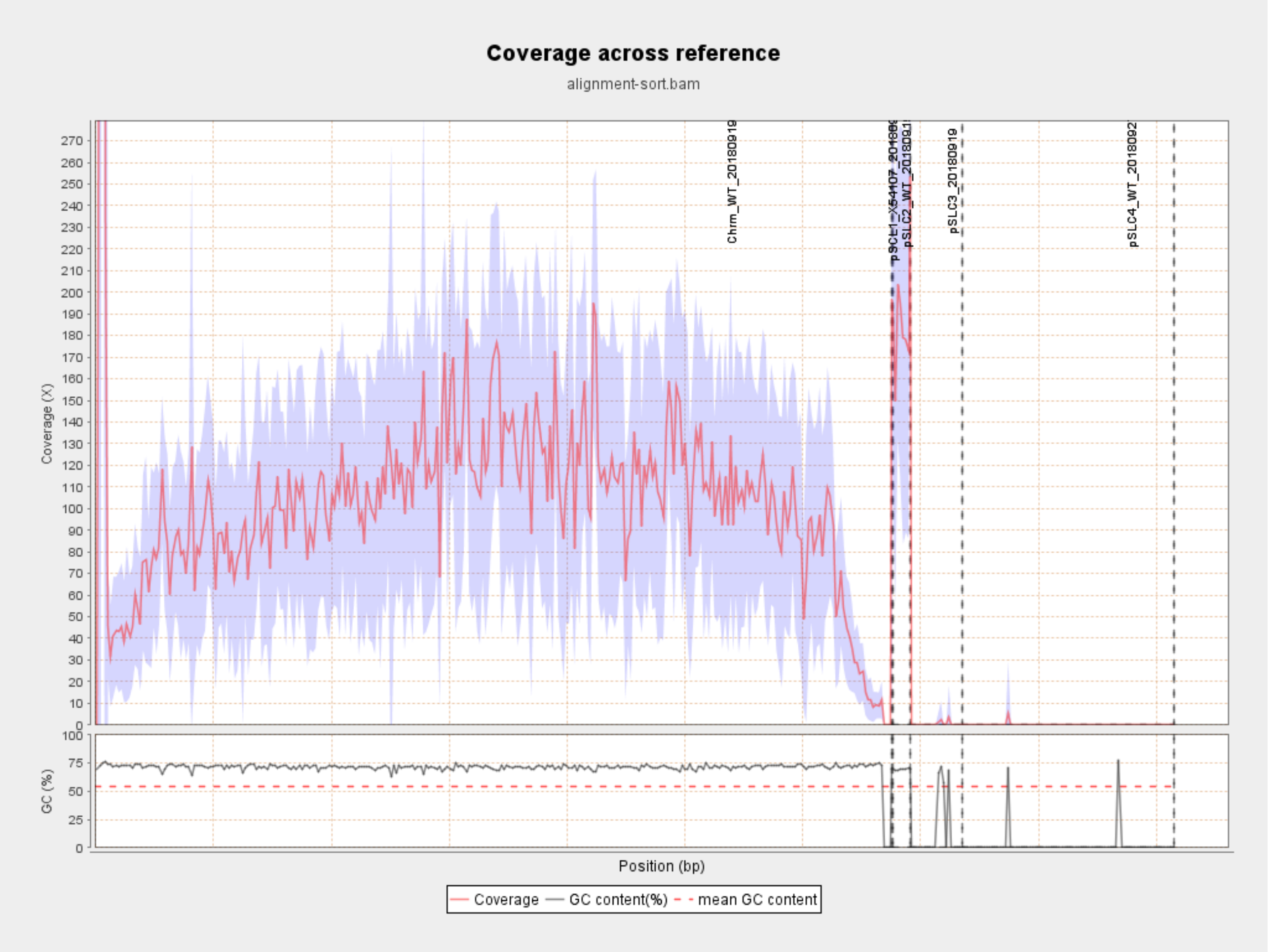

A detailed analysis of the alignment in GAP reveals the actual arrangement.

The actual peak in coverage starts at about 35 kb:

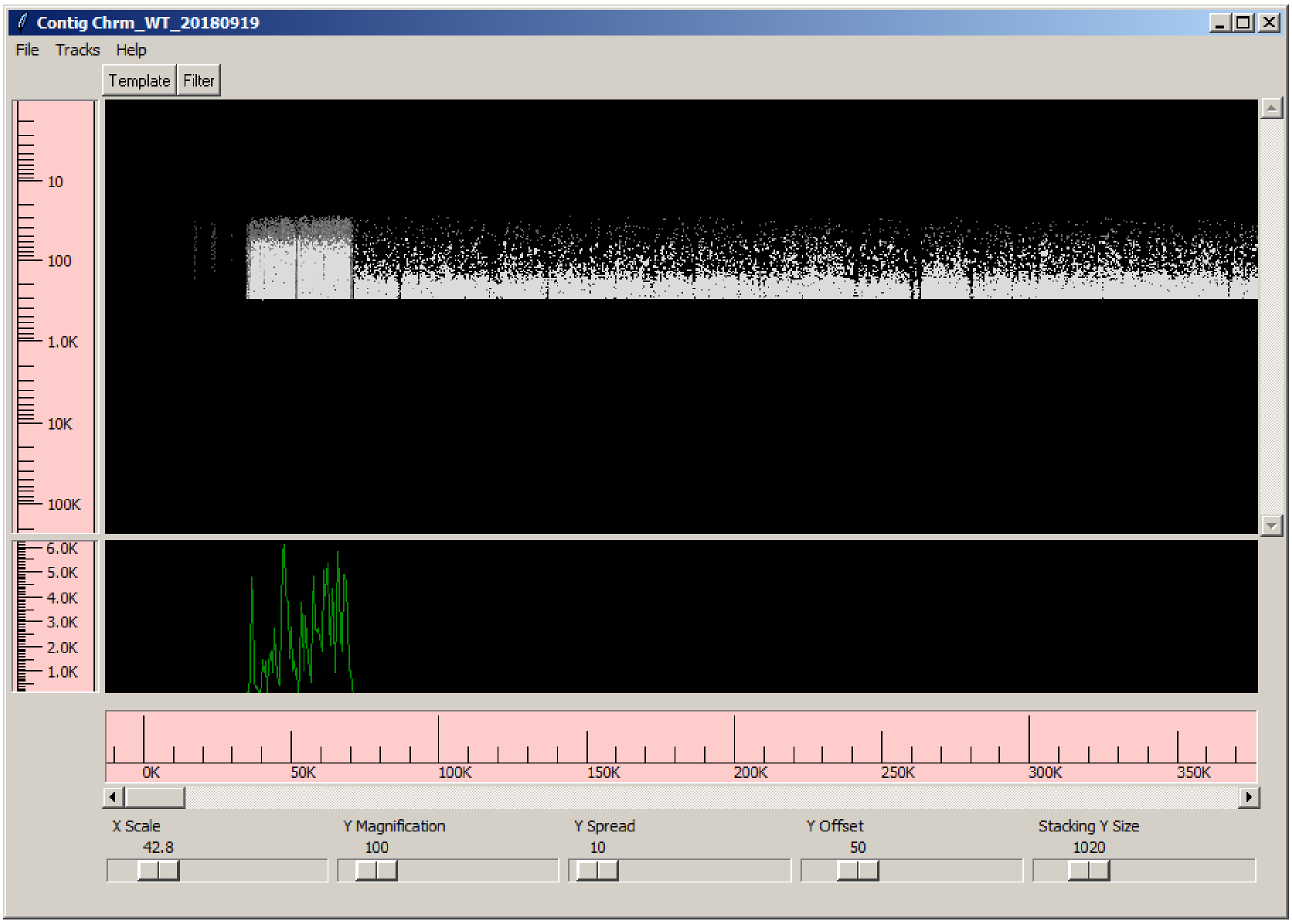

What agrees with the start of mapping of contigs:

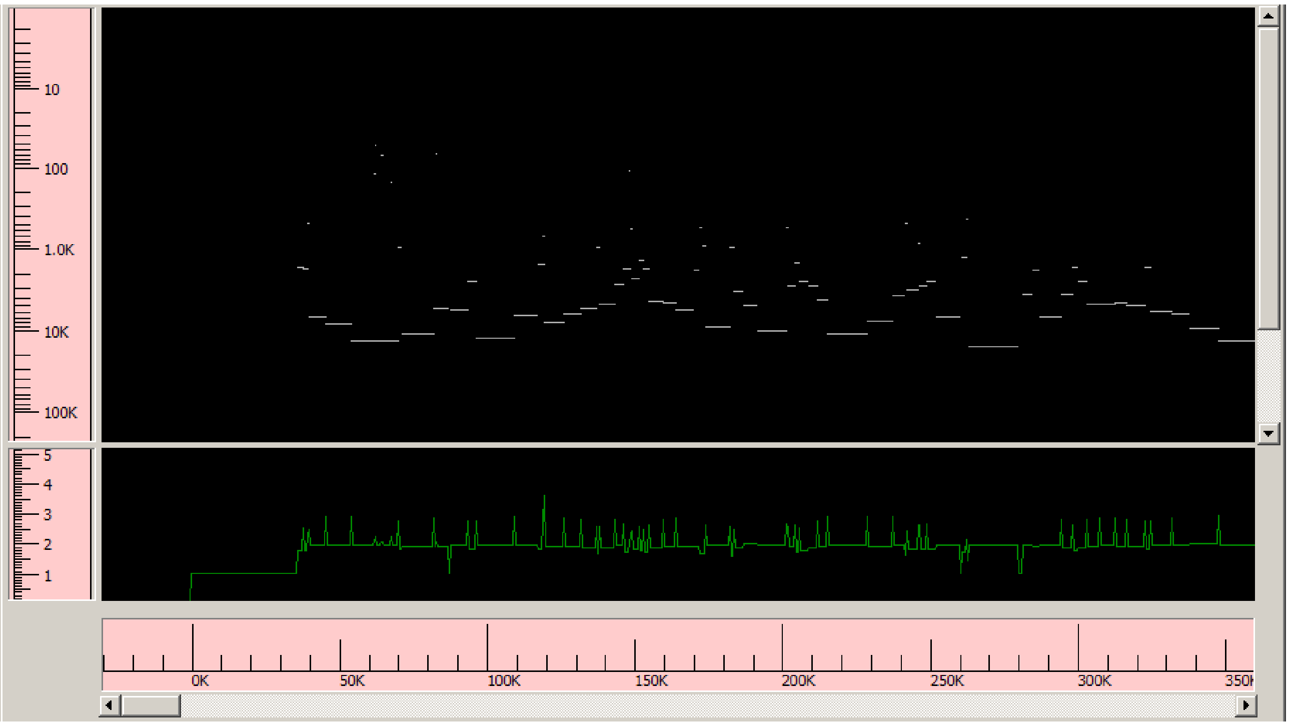

The peak finishes at about 70 kb, similarly to clone BW0217.

As for clone BW0217, there is no information at this left end that provides clues about any chromosomal modification. At the right end however, there is a sharp end of mapping:

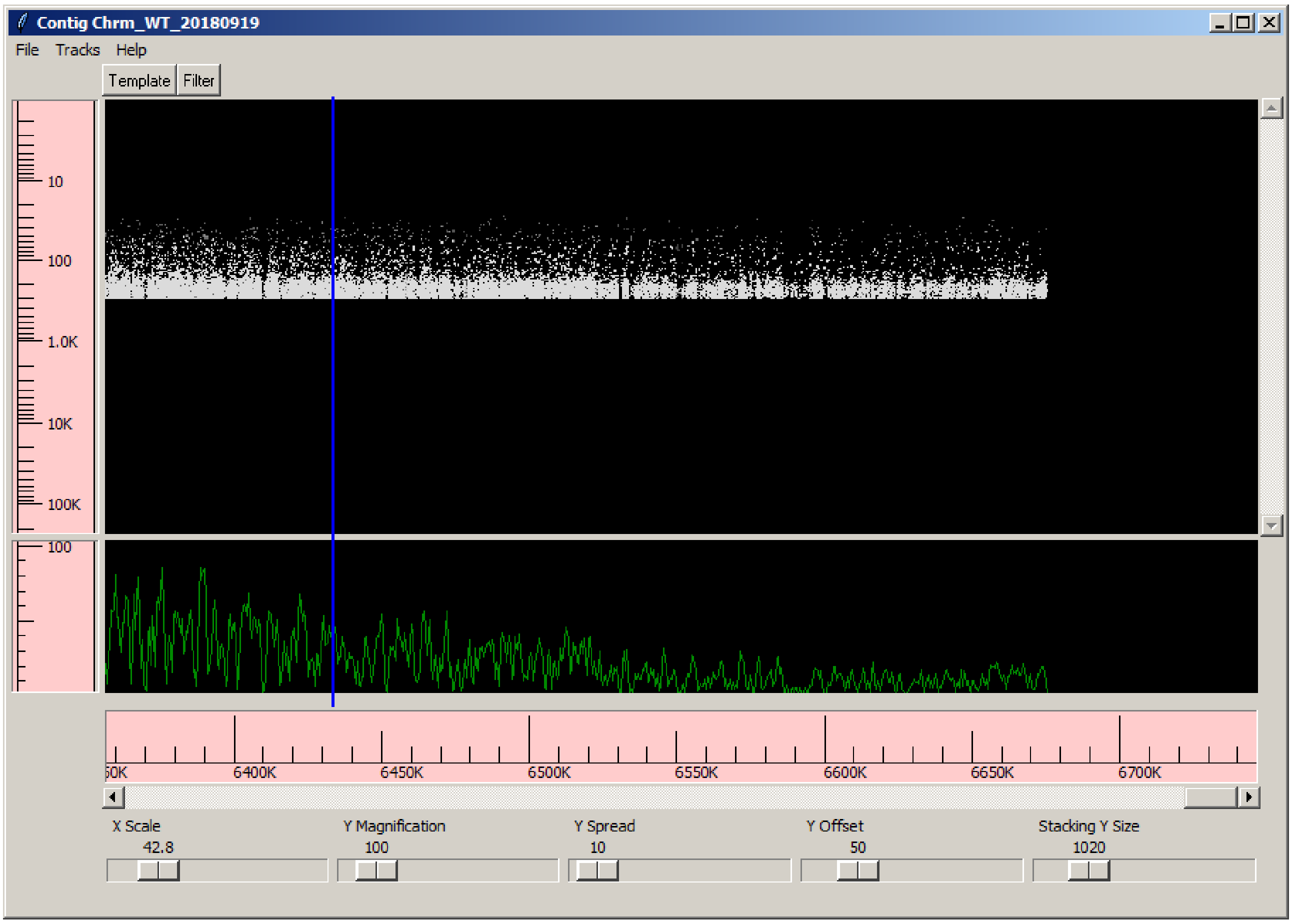

And it coincides with four reads that show extended non-matching sequence at 6675603:

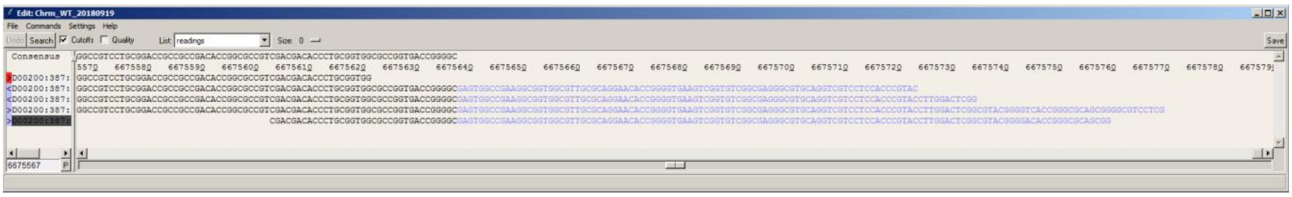

However, analysis revealed that there is an inverted repeat, for whatever reason, that doesn't give any further hint about specific rearrangements:

```
>lcl|Chrm_WT_20180919
Length=6748580

 Score =   305 bits (165),   Expect = 2e-083
 Identities = 165/165 (100%), Gaps = 0/165 (0%)
 Strand=Plus/Minus

Query  87       GCCCCGGTCACCGGCGCCACCGCAGGGTGTCGTCGACGGCGCCGGTGTCGGCGGCGGTCC  146
                ||||||||||||||||||||||||||||||||||||||||||||||||||||||||||||
Sbjct  6675431  GCCCCGGTCACCGGCGCCACCGCAGGGTGTCGTCGACGGCGCCGGTGTCGGCGGCGGTCC  6675372

Query  147      GCAGGACGGCCAGCCGGGTGAAGCGGCGTTCGAAGTCCTCGCGGGTCAGGCCGTGGGCCC  206
                ||||||||||||||||||||||||||||||||||||||||||||||||||||||||||||
Sbjct  6675371  GCAGGACGGCCAGCCGGGTGAAGCGGCGTTCGAAGTCCTCGCGGGTCAGGCCGTGGGCCC  6675312
```

```
Query  207      GGTAGGCGTCGGCGAGTTCGAGGGCGCCCCGTTTCACCGTCCACG  251
                |||||||||||||||||||||||||||||||||||||||||||||
Sbjct  6675311  GGTAGGCGTCGGCGAGTTCGAGGGCGCCCCGTTTCACCGTCCACG  6675267


 Score =  174 bits (94),  Expect = 5e-044
 Identities = 101/104 (97%), Gaps = 2/104 (2%)
 Strand=Plus/Plus

Query  1        GTACGGGTGGAGGACGACCTGCACGCCCTCGCCGACACCGACTTCACCCCGGTGTTCCTG  60
                ||||||||||||||||||||||||||||||||||||||||||||||||||||||||||||
Sbjct  6674831  GTACGGGTGGAGGACGACCTGCACGCCCTCGCCGACACCGACTTCACCCCGGTGTTCCTG  6674890

Query  61       CGCAACGCCACCGCCTTCGGCCACTCGCCCCGGTCACCGGCGCC  104
                |||||||||||||||||||||||||||||||| | ||| ||||
Sbjct  6674891  CGCAACGCCACCGCCTTCGGCCACTCGCCCCGG-CTCCG-CGCC  6674932
```

So, while it is clear that the ends have again been lost, there is insufficient evidence to support any conclusion regarding the topology of the replicon.

pSCL2 and pSCL1 are also completely covered by mapped reads and therefore we conclude they are still present in their original form.

Analysis of BW0219

The coverage map produced by Qualimap already shows a lack of a significant number of reads mapping to pSCL4 (as well as to pSCL3, as expected for the parental strain). This mutant has a more similar profile to clone A (BW0216), with ends clearly missing from any coverage:

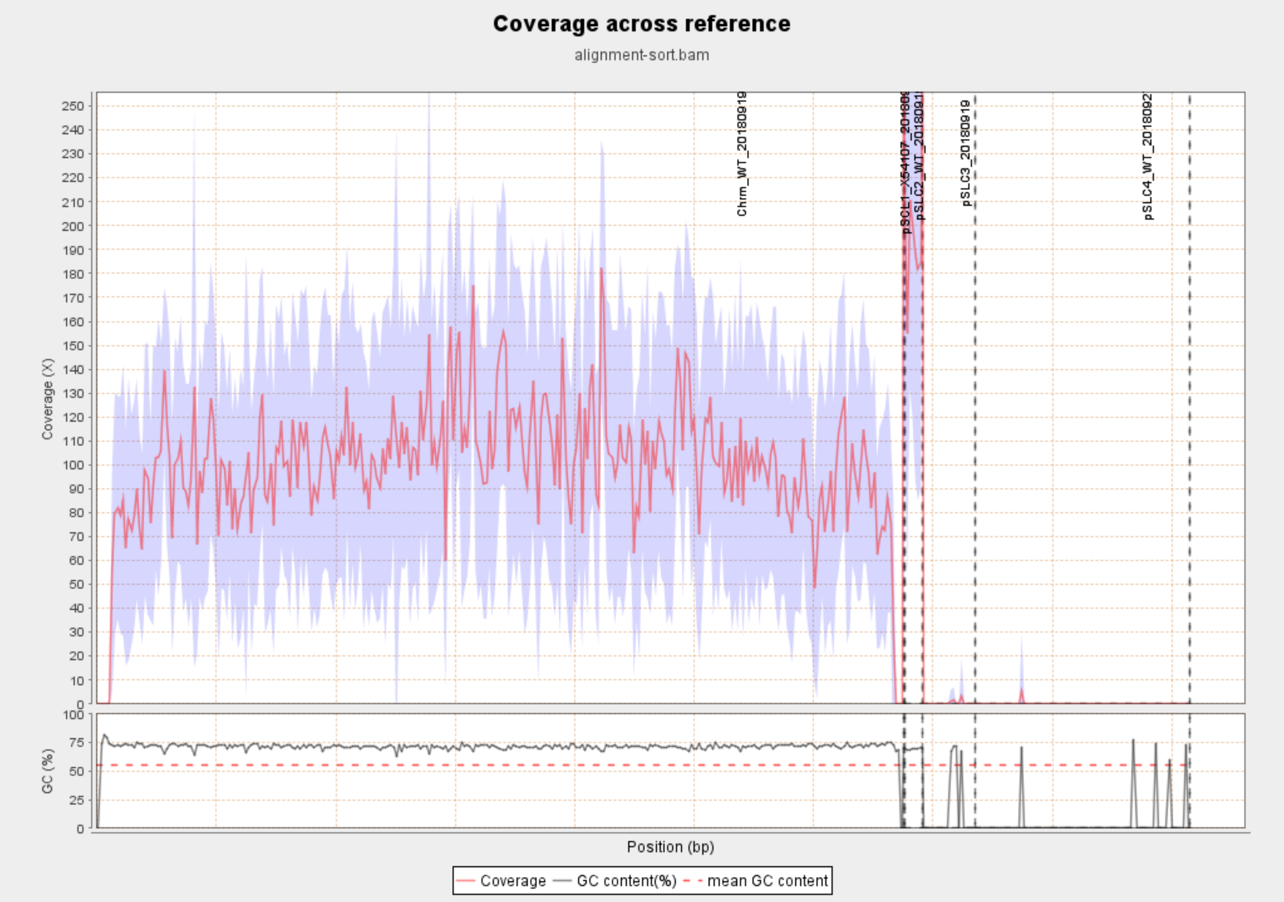

Detailed analysis in GAP5 reveals that there is negligible coverage until almost 120 kb and continues with quite homogeneous coverage:

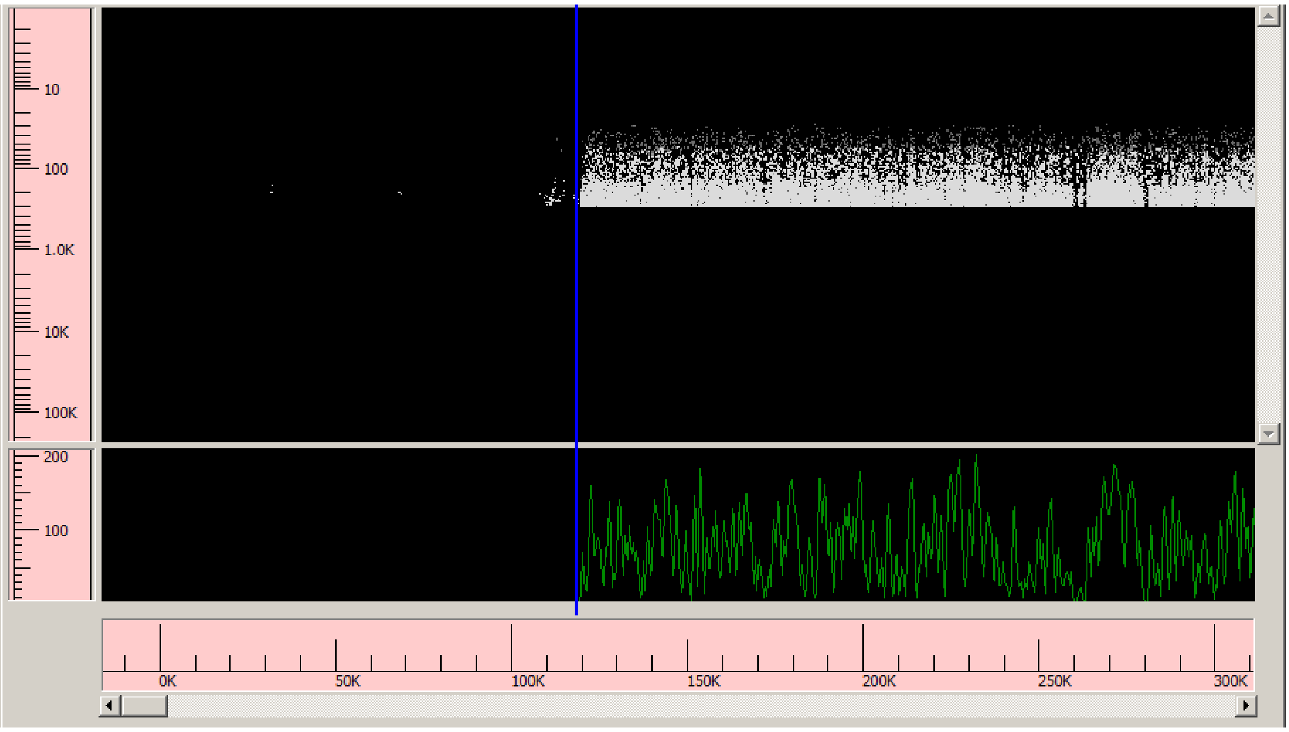

At this point, a lot of reads show a non-matching overhang, just as in clone BW0216:

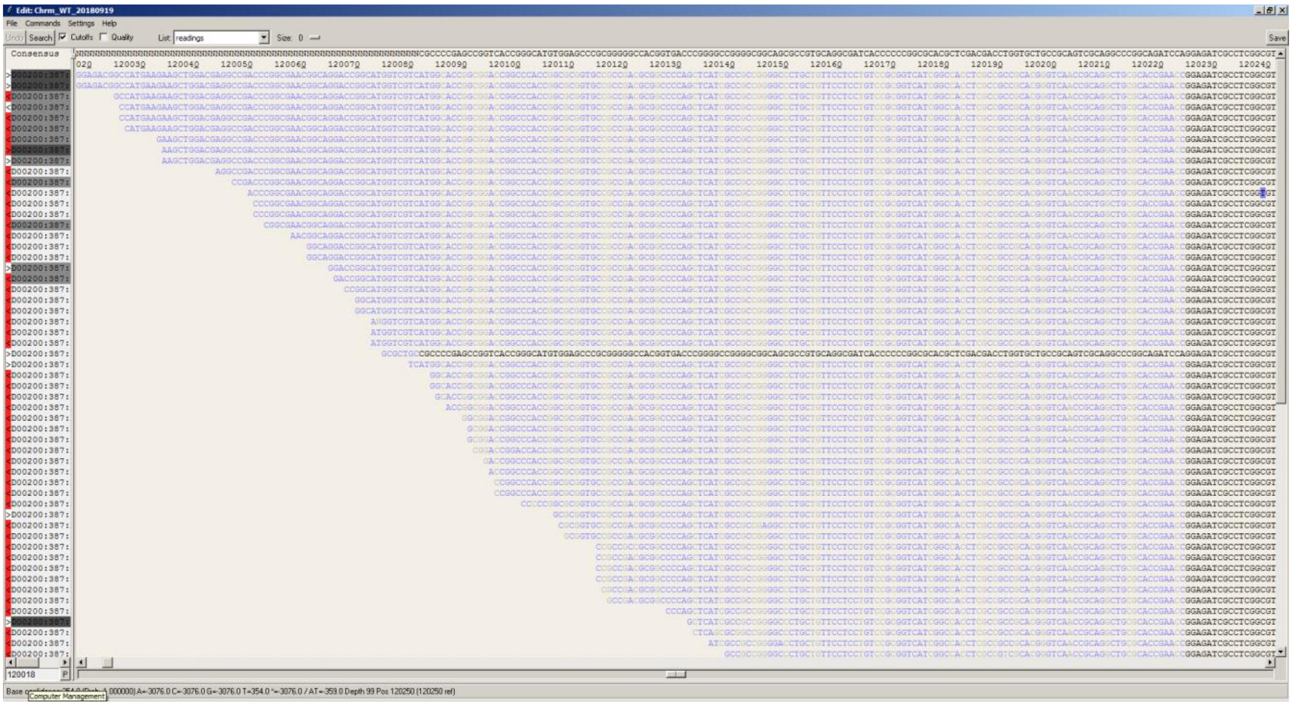

The same profile is shown by the contigs:

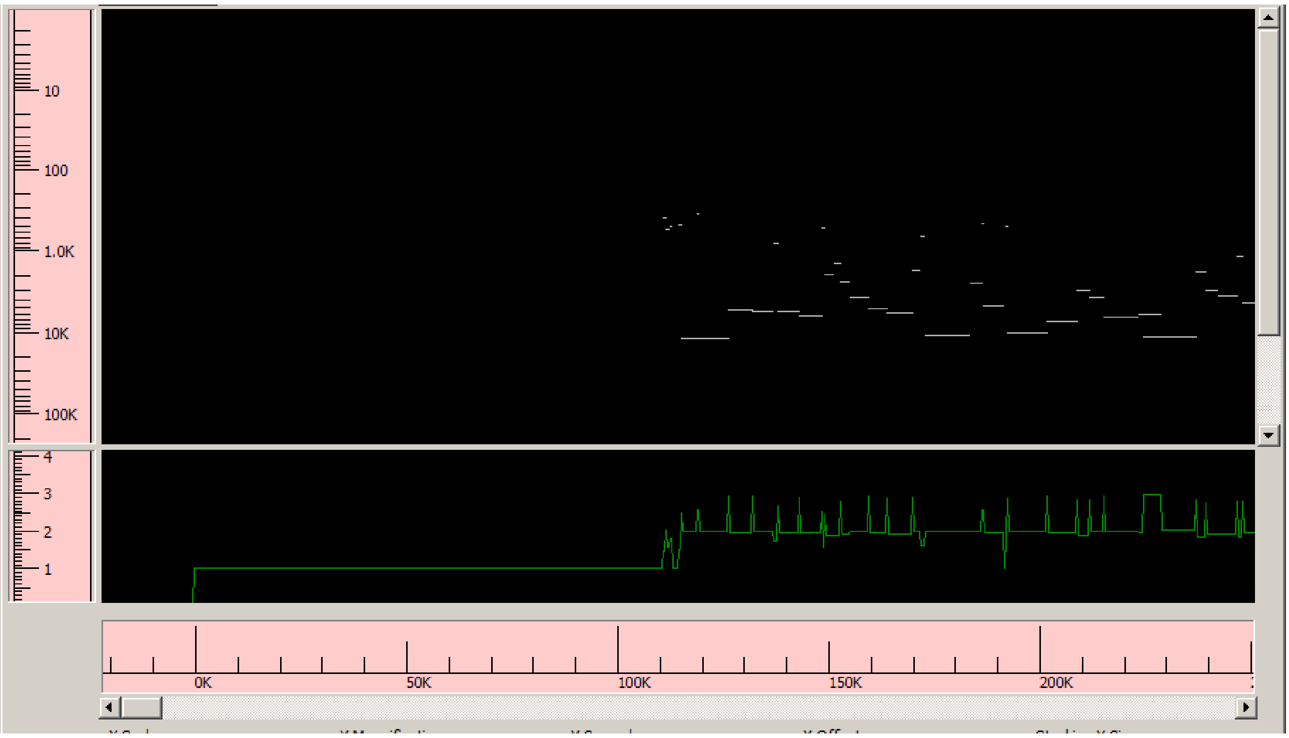

With one large contig that shows the same overhang at the same position:

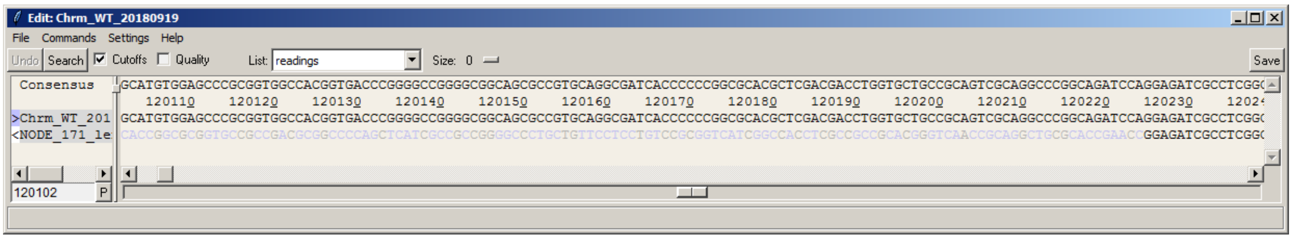

Same at the other end:

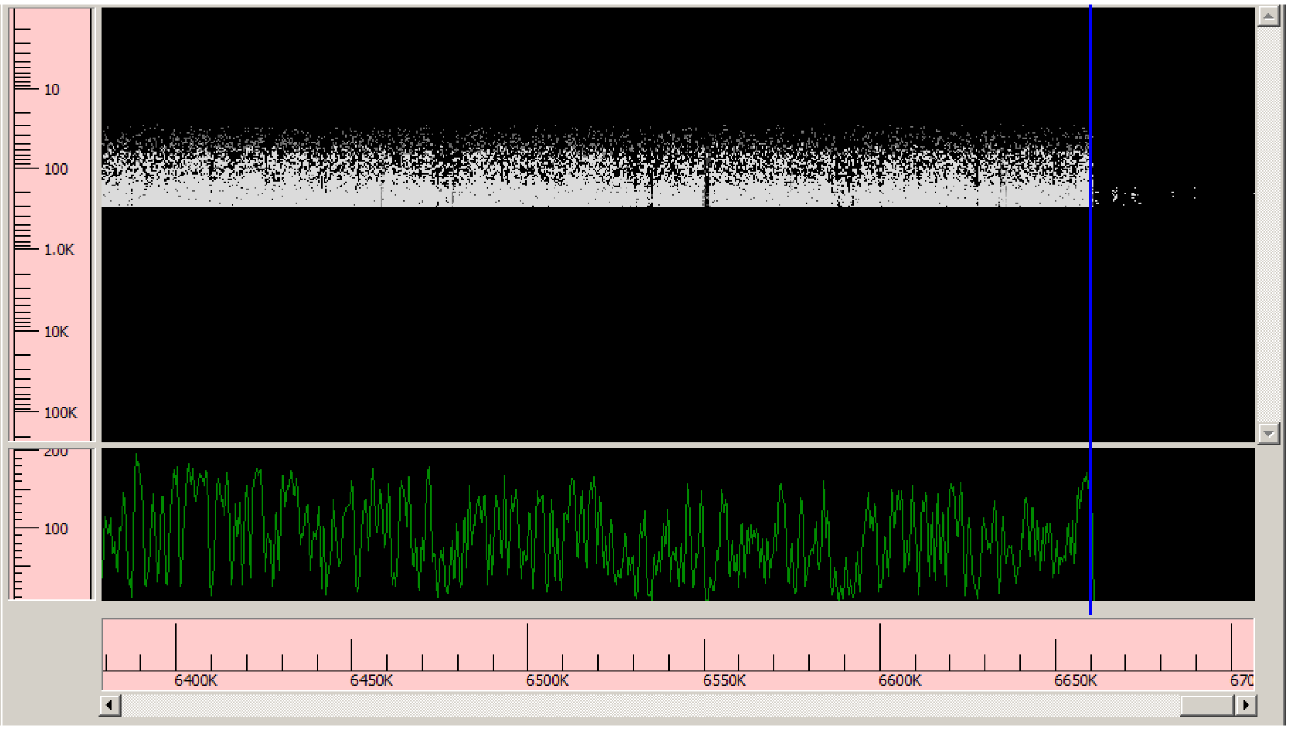

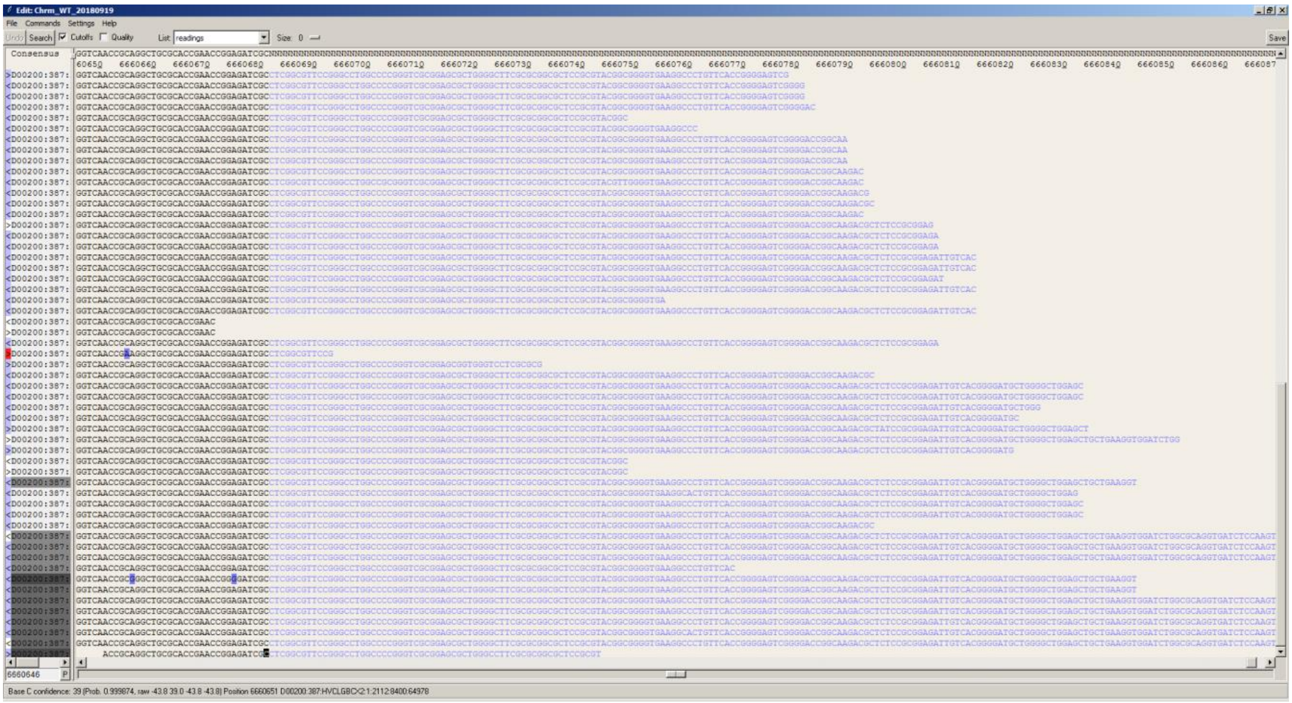

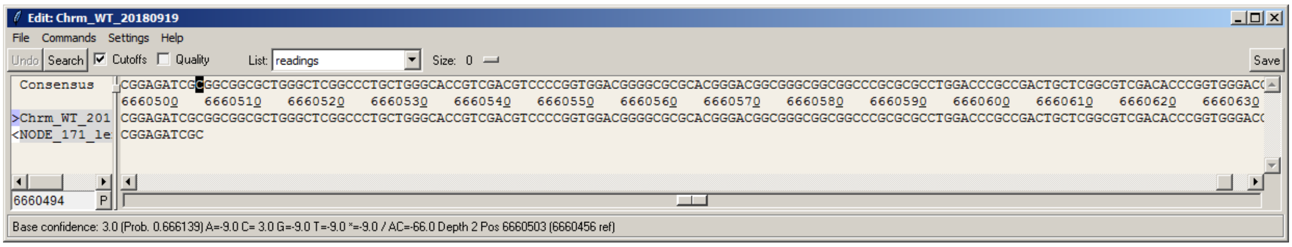

Using the contig for a blast against all replicons as before:

query id is: NODE_171_length_10901_cov_46.975

subject id is: Chrm_WT_20180919 Length=6748580

% identity	alignment length	mismatches	gap opens	q. start	q. end	s. start	s. end
99.96	4997	2	0	5883	10879	6660463	6655467
100.00	4499	0	0	1	4499	126206	121708
100.00	1412	0	0	4487	5898	121636	120225
100.00	30	0	0	10872	10901	6655479	6655508

The Blast output is not as easily interpreted as for BW0216. The last 30 bp HSP is unlikely to be significant and in any case falls within the first HSP on the sequence involved in circularisation. The other HSPs clearly indicate the circularisation of the chromosome with loss of the chromosome sequence from 1 to 120224 and from 6660464 to 6748580 (end of the known chromosome), but taken together the contig and reads ouput it seems clear that, as in mutant BW0216, mutant BW0219 seems to have a circular chromosome.

A view of the contig HSPs arrangement in Artemis illustrates clearer the chromosome circularisation:

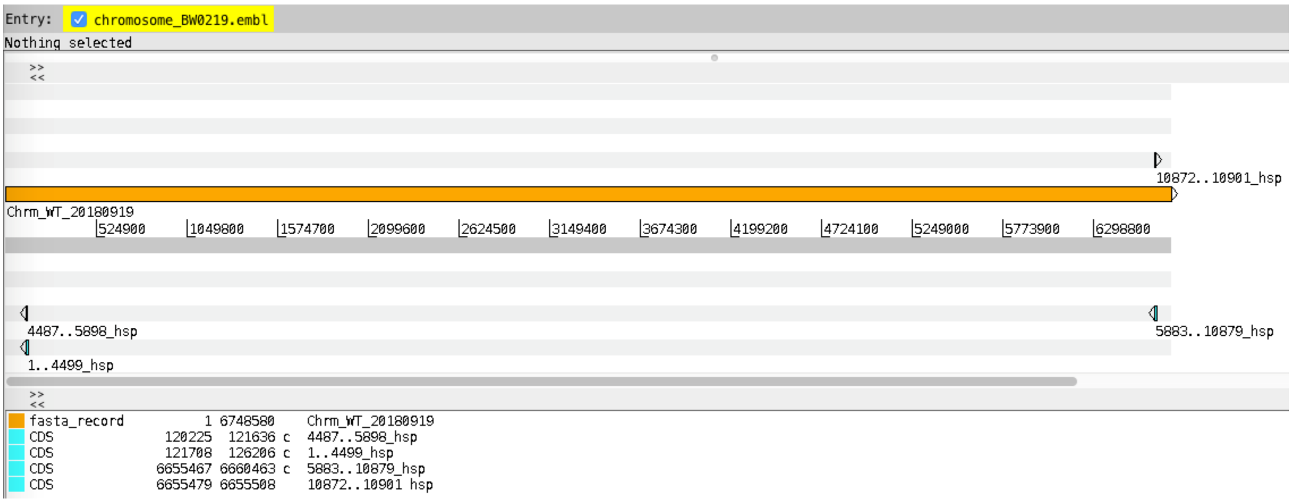

Regarding pSCL1 and pSCL2 they are both fully covered by reads.

Analysis of BW0220

The coverage map produced by Qualimap already shows a lack of a significant number of reads mapping to pSCL4 (as well as to pSCL3, as expected for the parental strain). Regarding the chromosome, it shows a pattern different to any other mutant, with a lack of coverage at the right end as others, but a different left end:

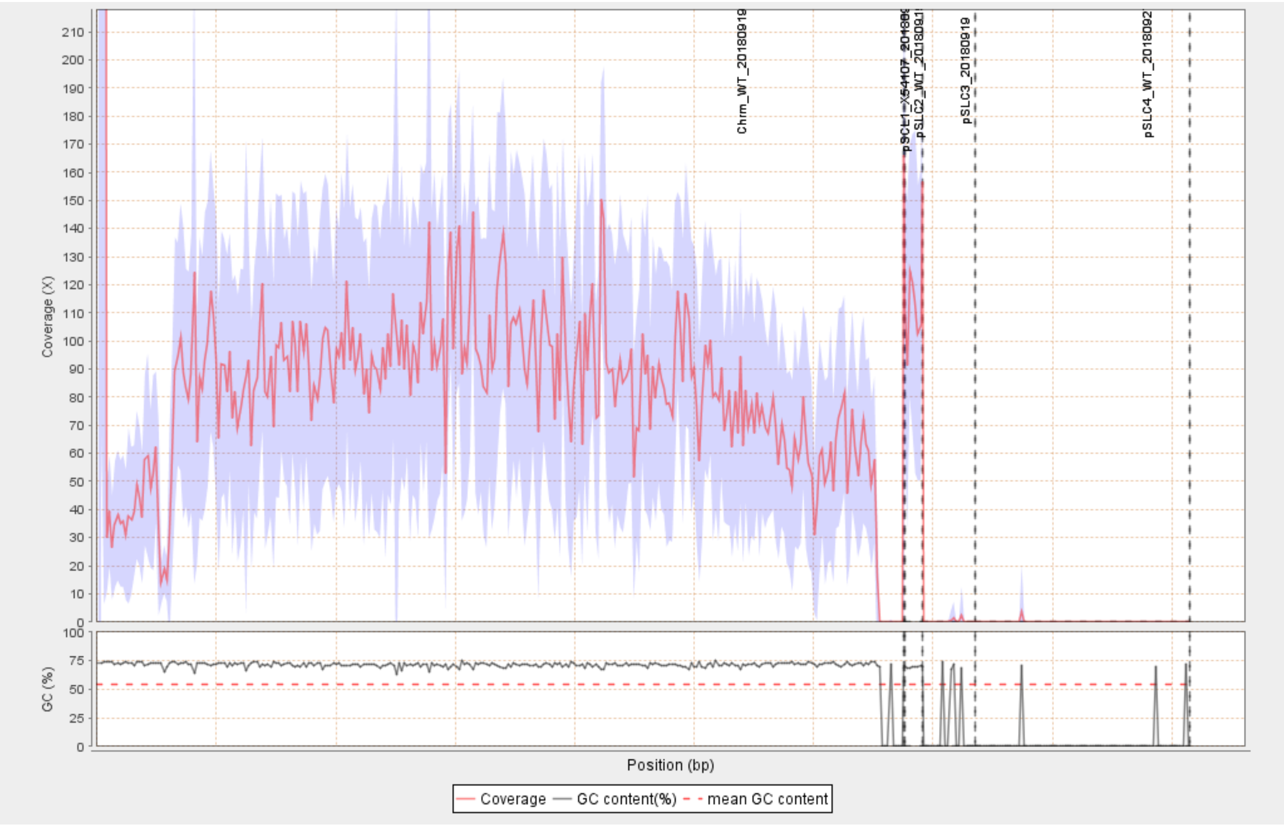

A detailed analysis in GAP5 shows a peak of coverage starting at about 19 kb:

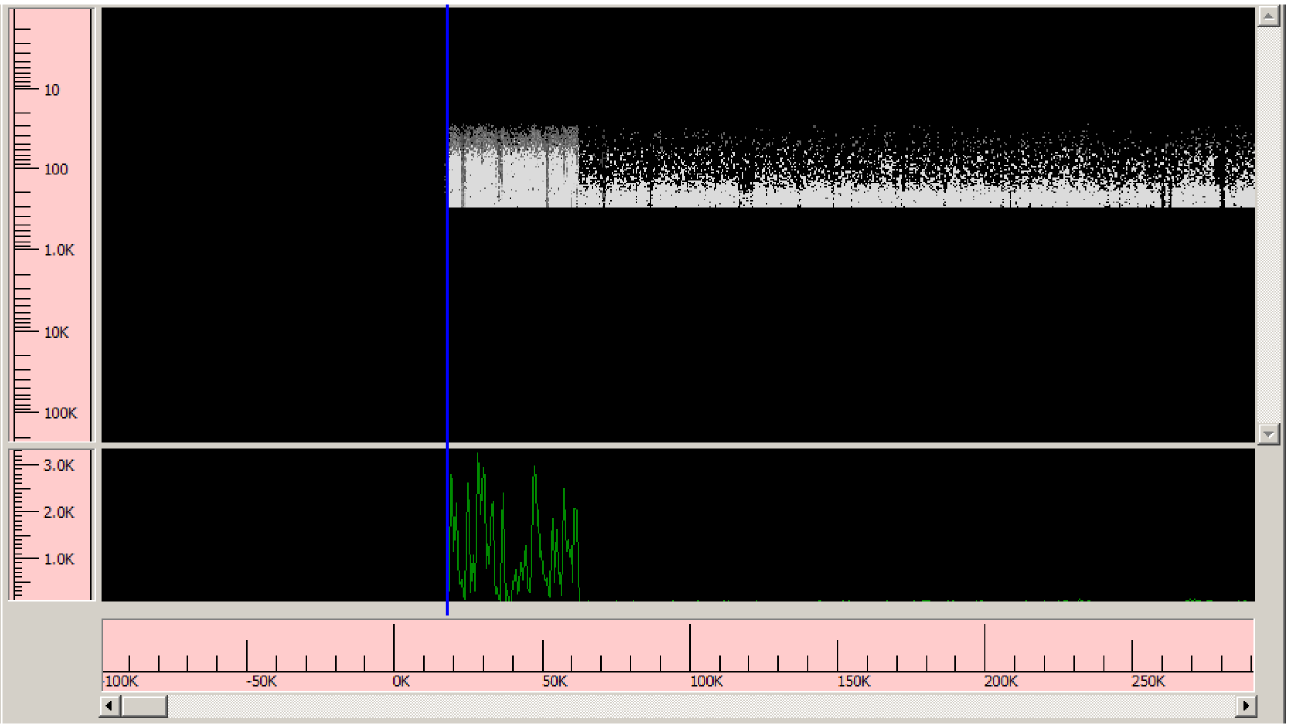

But without significant overhanging of reads:

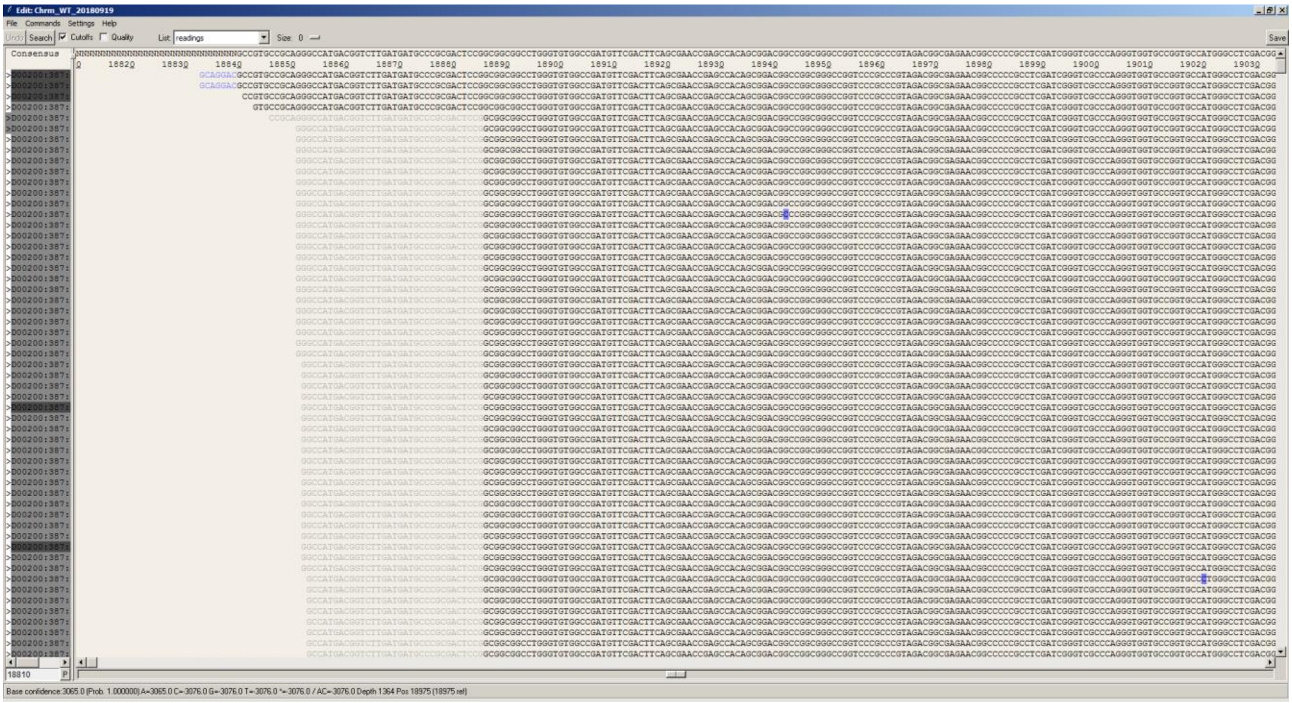

The high coverage ends abruptly at about 62.5 kb and the contiguity is provided by many reads that show a very conserved mismatch indicating that there might not exist such contiguity:

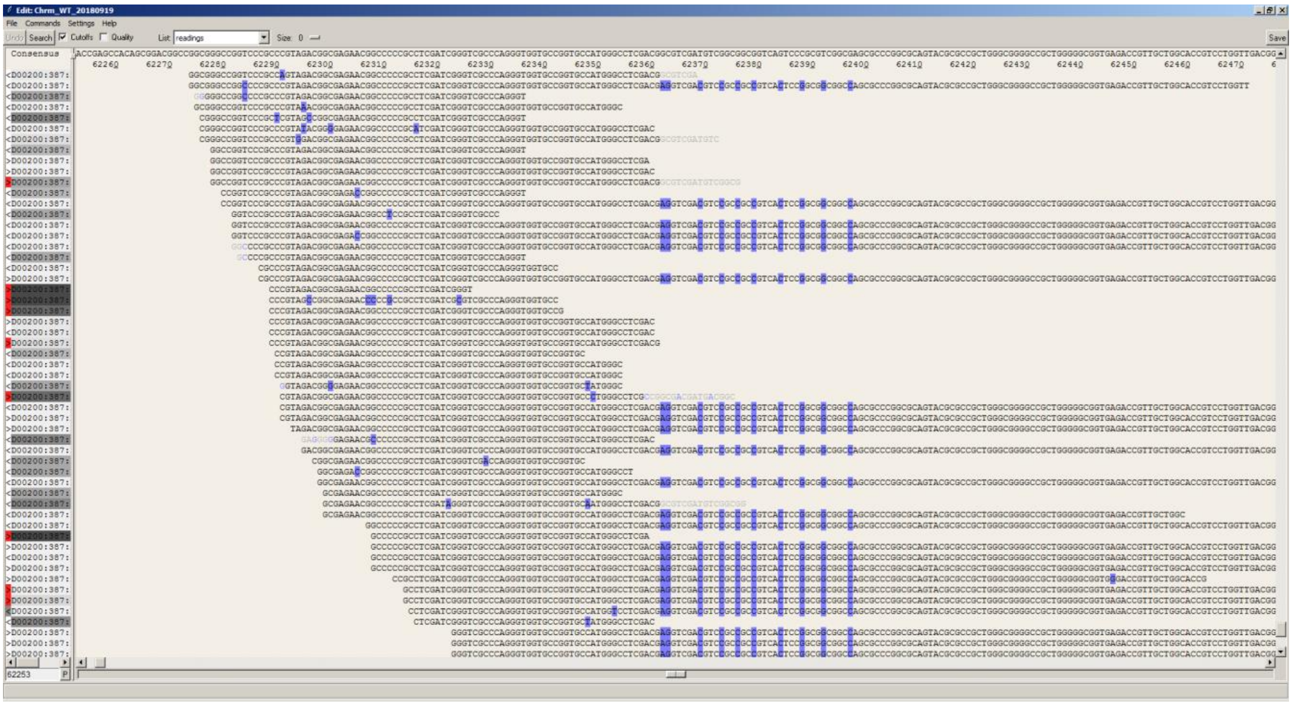

It is important to notice that this region contains the large type I PKS gene cluster.

The contigs do not provide any further information, so we cannot confirm the topology of the chromosome in this mutant.

Regarding pSCL1 and pSCL2 they are both fully covered by reads.

ANALYSIS OF ADDITIONAL *tpg-tap* GENES IN pSCL2

Several previous reports had indicated that the correct replication and maintenance of the ends of all linear replicons in this strain depended on the *tpg* and *tap* genes present in pSCL4, as these were the only ones present in the genome [31]. There is, nevertheless at least one paper, that identified the sequence of pSCL2-*tpg* [32]. Deletion of pSCL4 would therefore force either the circularisation of the replicons or even loss of the other plasmids. The fact that both pSCL1 and pSCL2 been maintained in their complete original form indicates that there must be another mechanism for maintenance of these linear replicons.

The analysis described below identified an additional tap-tpg *set in pSCL2. If there are two sets of tpg/tap in the genome, and losing the set in pSCL4 forces the circularisation of the chromosome but not of the remaining replicons, this implies that the set encoded in pSCL2 can provide functionality for both pSCL2 and pSCL1, but not the chromosome, and that the set encoded by pSCL4 would be responsible for maintenance of both pSCL4 and the chromosome.*

We thus performed a Blast search of our assembly and the known sequence of pSCL3 using the amino-acid sequence of TpgC and Tap from *Streptomyces coelicolor*:

```
>SCO7734_TpgC
MSLFGNGLDAAVQKAFTRPAPKSAGAQMRYLVKQLKGTKAVAQMLRVSQRTVERYVKNEI
KRPRPDLAARLEREVKARWQPQVRARARQKAATTDGIVIDTRARLGYTAPIGSTDQDRIR
HLTVALPPQYAGRLFDAHQAGATDQQLQGIAAEALKEVYFQDGGRRAGSLEEVRFTDIEH
LEFDL

>SCO7733_Tap
MTDQPQPLFAAVDALLAAVDDGGVLPVPDERVRLREAAGLTQAAVAQALGVRVASIQAWE
TGRAEPKAERIEAYRRLLEGLSRRFPASAVPPGSSEAAPRPDPGAPSPSAPAAPPSVLAA
VPSASAAAPTAPPRPAAPGRQPAARRADGKQSAACLRTAAAGGTDPRFENGPLAVVDVED
GQVRAYCTGGLVLDVPAKSLAALVDWTLAEAKLGQPKLSGPGKDADPLLVLTEAALDRYG
LPTTLTDEERLAGRIPEGHKAVKQLARAHWKLTKRGFGPWARIYRPATGSERACVQLCIP
SWHALDTRHWGHAGQLPPAELARVLGVYASRVMTPRGSTAVTGLELMTALHPPTRASQPD
AAGRRHSERNPGSLGQDPVDCAPCEAPDGHPLLAALPRFHVRGPAEKLFEEAYDWARPMT
DDECMLRYLVGIDVNMAFAAGANGLTVGLGAPTRVERPAFDAKLPGSWLVDLSHVDLSRV
KVGKEWAELDASLLPSPFTPKGERPEGPAWYATPTVAYAKELGYEVRPVEAWVRYENGRY
LDGWYQRLRDAYLATMSDLGVDADRTPDDFLAAMDGYRSRDPELAIVVSAMKATVKGGLG
KLRERPRGEGWKPGQPWRALSRPTWRPDIRAAVISRTRINLHRKIVKHAAFTGQYPIAIL
SDCVVYAAAGPSPLDFLPYRDGKPLPGGFKLGVNPGLVKHEGTQSVLWGEEVRERFDAPE
LNLARYIKDGTVTDADSGE
```

The results were quite clear: the best match for both proteins lies on pSCL2, while the match on pSCL4 shows less identity, providing very strong evidence that pSCL2 carries a full set of genes for Tap-Tpg:

```
Database: all_reps_20190129
           5 sequences; 9,149,446 total letters

Query= SCO7734_TpgC Length=185
                                                                  Score     E
Sequences producing significant alignments:                      (Bits)  Value

lcl|pSLC2_WT_20180919                                              265    2e-081
lcl|pSLC4_WT_20180927                                              164    4e-046
lcl|pSLC3_20180919                                                 120    3e-031
lcl|Chrm_WT_20180919                                              27.7    1.5
```

```
>lcl|pSLC2_WT_20180919
Length=149446

 Score =  265 bits (678),  Expect = 2e-081, Method: Compositional matrix adjust.
 Identities = 133/184 (72%), Positives = 159/184 (86%), Gaps = 0/184 (0%)
 Frame = -1

Query  2      SLFGNGLDAAVQKAFTRPAPKSAGAQMRYLVKQLKGTKAVAQMLRVSQRTVERYVKNEIK  61
              S FG GLD AV+KAFTRPAPK+AG +MRYLVK LKGTKAVA++L VSQRTVERYVK +IK
Sbjct  10750  SEFGGGLDTAVEKAFTRPAPKAAGTRMRYLVKHLKGTKAVAELLGVSQRTVERYVKGQIK  10571

Query  62     RPRPDLAARLEREVKARWQPQVRARARQKAATTDGIVIDTRARLGYTAPIGSTDQDRIRH  121
              +PRPDLAARLEREVKARWQPQ++A+AR+KAATT GI++D  AR+GYTAPIG+TDQDRIRH
Sbjct  10570  KPRPDLAARLEREVKARWQPQIKAKARKKAATTGGIILDIHARMGYTAPIGTTDQDRIRH  10391

Query  122    LTVALPPQYAGRLFDAHQAGATDQQLQGIAAEALKEVYFQDGGRRAGSLEEVRFTDIEHL  181
              +TVALPP++A RL  A   GA + +L+ + AEALKE YFQD GRRAGSLEEV+  D+ HL
Sbjct  10390  ITVALPPRHAARLLTAQDQGAGEDRLRELTAEALKETYFQDNGRRAGSLEEVKINDVVHL  10211

Query  182    EFDL  185
              +F+L
Sbjct  10210  DFEL  10199
 Score =  122 bits (305),  Expect = 9e-032, Method: Compositional matrix adjust.
 Identities = 71/174 (41%), Positives = 110/174 (63%), Gaps = 3/174 (2%)
 Frame = -3

Query  11    AVQKAFTRPAPKSAGAQMRYLVKQLKGT-KAVAQMLRVSQRTVERYVKNEIKRPRPDLAA  69
             A +K FTRPAPKSA +Q+++L+ + KG+ + +A+ +  S RT+ERY   ++K+P+  L A
Sbjct  5420  AERKVFTRPAPKSANSQVKFLLTRAKGSVRNLAERVGTSTRTIERYRAGKLKKPQKKLQA  5241

Query  70    RLEREVKARWQPQVRARARQKAATTDGIVIDTRARLGYTAPIGSTDQDRIRHLTVALPPQ  129
              L    ++ WQPQVRA+AR++A+TT G+++D  A  G+    G++D  R R +T A+ P
Sbjct  5240  ALLEATESEWQPQVRAQAREQASTTSGMMVDVTAYFGFVGK-GNSDDGRERSITTAISPT  5064

Query  130   YAGRLFDAHQAGATDQQLQGIAAEALKEVYFQDGGRRAGSLEEVRFTDIEHLEF  183
             YA ++     +GATD  L  + AEA+ E YF + G RA  L    FT +  +EF
Sbjct  5063  YAQQILQLQASGATDDDLYPVVAEAITESYFTEWGTRAKGL-RADFTHVVSIEF  4905


>lcl|pSLC4_WT_20180927
Length=1794824

 Score =  164 bits (414),  Expect = 4e-046, Method: Compositional matrix adjust.
 Identities = 93/183 (51%), Positives = 128/183 (70%), Gaps = 1/183 (1%)
 Frame = +2

Query  1       MSLFGNGLDAAVQKAFTRPAPKSAGAQMRYLVKQLKGTKAVAQMLRVSQRTVERYVKNEI  60
               M    + L  A Q+ FTR  PKS  A++R+L+ +LK TKAVA+ + V++R+VERY+  E
Sbjct  894599  MGDIDDALIRADQELFTREPPKSTQARVRFLLGRLKTTKAVAEAIGVTRRSVERYLSGER  894778

Query  61      KRPRPDLAARLEREVKARWQPQVRARARQKAATTDGIVIDTRARLGYTAPIGSTDQDRIR  120
               + P   +A R+E+EV+ RWQP+VR R R++AA   GI ++TRAR GYTAPIG+TD  R+R
Sbjct  894779  RTPPRPIADRIEKEVRLRWQPRVRERRRREAAARTGITVETRARFGYTAPIGTTDDPRMR  894958

Query  121     HLTVALPPQYAGRLFDAHQAGATDQQLQGIAAEALKEVYFQDGGRRAGSLEEVRFTDIEH  180
                LTV LPPQYA RLF A   GA +++L+ I AE L+ VYF+D G RA  L +V  TDI++
Sbjct  894959  RLTVHLPPQYADRLFTAQHEGAAERRLREIVAEGLQRVYFRDEGARAAGL-DVEITDIDY  895135

Query  181     LEF  183
               L+
Sbjct  895136  LDL  895144
```

```
>lcl|pSLC3_20180919
Length=444900

 Score =  120 bits (301),  Expect = 3e-031, Method: Compositional matrix adjust.
 Identities = 78/191 (41%), Positives = 111/191 (58%), Gaps = 6/191 (3%)
 Frame = -3

Query  1      MSLFGNGLDAAVQKAFTRPAPKSAGAQMRYLVKQLKGTKAVAQM------LRVSQRTVER  54
              + L  + L+ A + A+++P PK+ G ++R+L+KQ KG             +  SQRTVER
Sbjct  35695  VGLIEDRLEEAAESAYSQPIPKTLGGRIRFLLKQEKGMSRAEAARAVAEKIGKSQRTVER  35516

Query  55     YVKNEIKRPRPDLAARLEREVKARWQPQVRARARQKAATTDGIVIDTRARLGYTAPIGST  114
              Y  ++IK   P  AA +E EVK  WQP V+A+  Q A TT GI +D R + G+ +P G+T
Sbjct  35515  YRDDKIKSTSPATAALVEAEVKQLWQPIVKAKKFQAAITTGGIKVDVRGKFGFKSPKGTT  35336

Query  115    DQDRIRHLTVALPPQYAGRLFDAHQAGATDQQLQGIAAEALKEVYFQDGGRRAGSLEEVR  174
              +  R R LT  L    A  LF A  AGA++  L  I A+ L++ YF DGG RA SL+ V
Sbjct  35335  NDPRYRRLTRRLDATTAAALFAAKAAGASEDDLAQIVADGLRDDYFTDGGSRAHSLQTVE  35156

Query  175    FTDIEHLEFDL  185
               T I+H+ F +
Sbjct  35155  LTGIDHIRFSI  35123
```

```
Database: all_reps_20190129
           5 sequences; 9,149,446 total letters
```

Query= SCO7733_Tap Length=739

```
                                                                      Score     E
Sequences producing significant alignments:                          (Bits)  Value

lcl|pSLC2_WT_20180919                                                 999    0.0
lcl|pSLC4_WT_20180927                                                 576    2e-180
lcl|pSLC3_20180919                                                    481    9e-148
lcl|Chrm_WT_20180919                                                  33.5    0.17


>lcl|pSLC2_WT_20180919
Length=149446

 Score =  999 bits (2584),  Expect = 0.0, Method: Compositional matrix adjust.
 Identities = 533/719 (74%), Positives = 590/719 (82%), Gaps = 12/719 (2%)
 Frame = -1

Query  26     PVPDERVRLREaagltqaavaqalgvRVASIQAWETGRAEPKAERIEAYRRLLEGLSRRF  85
              PVP ER RLREAAGL+QA +A AL  R  ++  WETG+ EP+  +  AY RLLEGL++RF
Sbjct  12907  PVPAERRRLREAAGLSQAQIAAALEARREAVGNWETGKTEPRPPKRAAYARLLEGLAQRF  12728

Query  86     PASAVPPGSSEaaprpdpgapspsapaappsvlaavpsasaaaptapprpaapgrqpaar  145
                    P  +  AP   P  P        P+ ++A  + +  AP A  RP    +  A+
Sbjct  12727  ------PAPASDAPAVAPTPPVVPEAFTGPASVSAPAAEAPTAPAAAVRPVPAAKPAASS  12566

Query  146    raDGKQSAACLRTAAAGGTDPRFENGPLAVVDVE-DGQVRAYCTGGLVLDVPAKSLAALV  204
              R  G + AA   T A GG DPRFENGPL VVD + +G V AYCTGGL+LDVPAKSL +LV
Sbjct  12565  RRPGAKKAAPANTPA-GGADPRFENGPLVVVDADTNGTVVAYCTGGLILDVPAKSLPSLV  12389

Query  205    DWTLAEAKLGQPKLSGPGKDADPLLVLTEAALDRYGLPTTLTDEERLAGRIPEGHKAVKQ  264
              DWTL EAKLGQPKLSGPG+ ADPLLVLTEAA +RYGLP  L++EER+AGRIPEGHK +KQ
Sbjct  12388  DWTLREAKLGQPKLSGPGRPADPLLVLTEAACERYGLPVRLSEEERIAGRIPEGHKVIKQ  12209

Query  265    LARAHWKLTKRGFGPWARIYRPATGSERACVQLCIPSWHALDTRHWGHAGQLPPAELARV  324
              L RA WKLTKRGFGPWARIYRPA GS+R CVQLCIPSW ALD R W  A QLPPAELARV
Sbjct  12208  LVRADWKLTKRGFGPWARIYRPAKGSDRQCVQLCIPSWTALDARFWDGAAQLPPAELARV  12029

Query  325    LGVYASRVMTPRGSTAVTGLELMTALHPPTRASQPDAAG--RRHSERNPGSLGQDPVDCA  382
              LGVYA+RVMTPRGSTAVT LELMTALHPPT A + +  G  R+   + PGSLG DPVDCA
Sbjct  12028  LGVYATRVMTPRGSTAVTALELMTALHPPTHAVRDEETGVLRQAGTKTPGSLGSDPVDCA  11849

Query  383    PCEAPDGHPLLAAL--PRFHVRGPAEKLFEEAYDWARPMTDDECMLRYLVGIDVNMAFAA  440
              PCEAPDGHP+L AL  PRFHVRGPAEKLFEEAYDWARPMTD EC LRYLVG+DVNMAFAA
Sbjct  11848  PCEAPDGHPVLTALDLPRFHVRGPAEKLFEEAYDWARPMTDAECTLRYLVGLDVNMAFAA  11669

Query  441    GANGLTVGLGAPTRVERPAFDAKLPGSWLVDLSHVDLSRVKVGKEWAELDASLLPSPFTP  500
              GANGL VGLG PT V+  AFD KLPGSWLVDLSHV LSRVK GKEW  LD  LLPSPFTP
Sbjct  11668  GANGLNVGLGEPTHVKGLAFDPKLPGSWLVDLSHVSLSRVKAGKEWVALDGGLLPSPFTP  11489

Query  501    KGERPEGPAWYATPTVAYAKELGYEVRPVEAWVRYENGRYLDGWYQRLRDAYLATMSDLG  560
              KG+RP GPAWYATPTVAYA ELGYEVRP+EAWVRY+NGRYLDGWYQRLRDAYLATM+DLG
Sbjct  11488  KGDRPTGPAWYATPTVAYAVELGYEVRPLEAWVRYDNGRYLDGWYQRLRDAYLATMADLG  11309

Query  561    VDADRTPDDFLAAMDGYRSRDPELAIVVSAMKATVKGGLGKLRERPRGEGWKPGQPWRAL  620
              VDAD  P DFL AMDGY+ RDPEL IV++A+KATVKGG+GKLRERPRGEGW+PG+PWRAL
Sbjct  11308  VDADLAPADFLTAMDGYKERDPELGIVITAIKATVKGGIGKLRERPRGEGWRPGKPWRAL  11129

Query  621    SRPTWRPDIRAAVISRTRINLHRKIVKHAAFTGQYPIAILSDCVVYAAAGPSPLDFLPYR  680
              +RPTWRPDIRAAVISRTRINLHRKI+KHAAFTGQYP+AI+SDCVVYAA GPSPLDFLPYR
Sbjct  11128  ARPTWRPDIRAAVISRTRINLHRKIIKHAAFTGQYPVAIMSDCVVYAANGPSPLDFLPYR  10949

Query  681    DGKPLPGGFKLGVNPGLVKHEGTQSVLWGEEVRERFDAPELNLARYIKDGTVTDADSGE  739
              +GKPLPGGFKLG+NPGLVKHEGTQ +LWGEEVRE+FDAPELNLARYIKDGTVT  D+GE
Sbjct  10948  EGKPLPGGFKLGINPGLVKHEGTQPLLWGEEVREKFDAPELNLARYIKDGTVTGTDNGE  10772
```

```
>lcl|pSLC4_WT_20180927
Length=1794824

 Score =  576 bits (1485),  Expect = 2e-180, Method: Compositional matrix adjust.
 Identities = 319/583 (55%), Positives = 391/583 (67%), Gaps = 39/583 (7%)
 Frame = +1

Query  165     DPRFENGPLAVVDVEDGQVRAYCTGGLVLDVPAKSLAALVDWTLAEAKLGQPKLSGPGKD  224
               DPRF +GPLAV+D   G   AY  GG++L+ PA ++  LV W L E+ +G  +L   GKD
Sbjct  892933  DPRFPSGPLAVLD---GDGTAYAAGGVLLECPAGTVPELVAWALTESGIGAARLHRHGKD  893103

Query  225     ADPLLVLTEAALDRYGLPTTLTDEERLAGRIPEGHKAVKQLARAHWKLTKRGFGPWARIY  284
               ADPLLVLT  A  R GLP  L D   L  R+P  H  VK++ RA WKLT+RGFGPW RIY
Sbjct  893104  ADPLLVLTAPAAARLGLPLVLEDRRGL--RLPADHPVVKEIGRAGWKLTQRGFGPWPRIY  893277

Query  285     RPATGSERACVQLCIPSWHALDTRHWGHAGQLPPAELARVLGVYASRVMTPRGSTAVTGL  344
               RPA   +R CVQL +   W ALD R WG A  L  AELAR L  YA+RV+TPRGSTAV+GL
Sbjct  893278  RPAKAGQRQCVQLAVLPWGALDPRAWGEAASLDAAELARTLTTYATRVLTPRGSTAVSGL  893457

Query  345     ELMTALHPPTRASQPDAAGRRHSERNPGSLGQDPVDCAPCEAPDGHPLLAALP--RFHVR  402
               ELMTAL PPTRA + +  G   S  NPG+L + PVD AP EAP  HP+    P  RF
Sbjct  893458  ELMTALRPPTRAVKDETTGTWVSAPNPGALTR-PVDPAPPEAPAEHPVAQGWPADRF---  893625

Query  403     GPAEKLFEEAYDWARP---MTDDECMLRYLVGIDVNMAFAAGANGLTVGLGAPTRVERPA  459
                    L EEAY W R    + D+EC+L Y VGID+N AF A A  + +GLG P  V  PA
Sbjct  893626  -----LDEEAYQWVRDPELLADEECLLPYAVGIDINTAFLAAAARMPIGLGDPVHVAFPA  893790

Query  460     FDAKLPGSWLVDLSHVDLSRVKVGKEWAELDASLLPSPFTPKGERPEGPAWYATPTVAYA  519
               FD K+PGSWLVDLS V +                LPSPFTP G RP GPAWYATPTVAYA
Sbjct  893791  FDQKIPGSWLVDLSAVAID-------------PRLPSPFTPSGARPTGPAWYATPTVAYA  893931

Query  520     KELGYEVRPVEAWVRYENGRYLDGWYQRLRDAYLATMSDLGVDADRTPDD--FLAAMDGY  577
                +LG ++RP+E ++RY      LD W++RLRDAY+ TM+DLGV   +  D+  FLAAMDG+
Sbjct  893932  AQLGADIRPLEGYLRYGTAPCLDPWHERLRDAYVQTMADLGVPVAKDADEHTFLAAMDGH  894111

Query  578     RSRDPELAIVVSAMKATVKGGLGKLRERPRGEGWKPGQPWRALSRPTWRPDIRAAVISRT  637
               + RDP LA V+SA+K+TVKGG+GKLRER +G G++ G+ W AL RPTWRPD+RAAVI++
Sbjct  894112  KQRDPGLAAVLSAIKSTVKGGIGKLRERSQGRGYRDGERWPALERPTWRPDVRAAVIAKA  894291

Query  638     RINLHRKIVKHAAFTGQYPIAILSDCVVYAAAGPSPLDFLPYR-DGKPLPGGFKLGVNPG  696
               R+N+HRK+VK A  TG++P+A LSDCVVY +AGPSPLDFLP   +G+ LPG F+LG  PG
Sbjct  894292  RVNMHRKMVKTAETTGRHPLAALSDCVVYPSAGPSPLDFLPLTPEGRVLPGSFRLGATPG  894471

Query  697     LVKHEGTQSVLWGEEVRERFDAPELNLARYIKDGTVTDADSGE  739
               L K EG Q + W  ++ E+     LN AR+IKDG     D GE
Sbjct  894472  LAKLEGVQEMAWAVDLMEK----GLNPARHIKDGVDAVLDEGE  894588


>lcl|pSLC3_20180919
Length=444900

 Score =  481 bits (1238),  Expect = 9e-148, Method: Compositional matrix adjust.
 Identities = 284/576 (49%), Positives = 357/576 (62%), Gaps = 29/576 (5%)
 Frame = -1

Query  166    PRFENGPLAVVDVEDGQVRAYCTGGLVLDVPAKSLAALVDWTLAEAK--LGQPKLSGPGK  223
              P + +GPLAV+D+ DG + A+   G  +  PAK+L AL  W L + K  LG  K+   G
Sbjct  37389  PEYADGPLAVLDITDGALTAHLADGRTVPCPAKTLQALAAWALDKKKIRLGAAKVHENGF  37210

Query  224    DADPLLVLTEAALDRYGLPTTLTDEERLAGRIPEGHKAVKQLARAHWKLTKRGFGPWARI  283
              D DPLL+LT +A+  +GLP  L D+E L  R+P+ HK VK L +A WKLTK GFGPW RI
Sbjct  37209  DQDPLLILTTSAVAHFGLPADLADQEGL--RLPDDHKVVKNLTKAGWKLTKSGFGPWPRI  37036

Query  284    YRPATGSERACVQLCIPSWHALDTRHWGHAGQLPPAELARVLGVYASRVMTPRGSTAVTG  343
              ++   G +R CVQ+ +   W AL+ R W  AG LPP +LAR+LG YA  V+TPRG+ AVTG
Sbjct  37035  FKRLDG-KRHCVQVFLTPWGALENRVWSGAGDLPPGDLARMLGTYARLVITPRGTVAVTG  36859

Query  344    LELMTALHPPTRASQPDAAGRRHSERNPGSLGQDPVDCAPCEAPDGHPLLAA-LPRFHVR  402
```

```
                ELMT+L PPTRA   DA  +  S   P +L    VD APCEAPD HP++ A  P    R
Sbjct  36858  EELMTSLRPPTRAEWSDAEKKYVSATVPHTL-HTVVDPAPCEAPDEHPVVVADYPEEGSR  36682

Query  403    GPAEKLFEEAYDWARP---MTDDECMLRYLVGIDVNMAFAAGANGLTVGLGAPTRVER-P  458
                +E L EEA  W RP   +TD E    ++  +DV +AF A    L VG G      R P
Sbjct  36681  PASEALDEEACIWVRPADLVTDAERACTHVAALDVQVAFLAACRRLHVGTGPAIHYPRNP  36502

Query  459    AFDAKLPGSWLVDLSHVDLSRVKVGKEWAELDASLLPSPFTPKGERPEGPAWYATPTVAY  518
              AFD  LPGSW VDLS ++                    LPSPFT  GERPEGP WYATPTVAY
Sbjct  36501  AFDPTLPGSWYVDLSRIESD-------------PRLPSPFTSSGERPEGPGWYATPTVAY  36361

Query  519    AKELGYEVRPVEAWVRYENGRYLDGWYQRLRDAYLATMSDLGVDADRTPDDFLAAMDGYR  578
              A ELG +VRP+EAWVR  +  YL  WY  LRDAYL  M+DLGV  D   + FLAAM  Y+
Sbjct  36360  AAELGADVRPMEAWVRETHSPYLQPWYDTLRDAYLTVMADLGVTKDLDDEAFLAAMAAYK  36181

Query  579    SRDPELAIVVSAMKATVKGGLGKLRERPR-GEGWKPGQPWRALSRPTWRPDIRAAVISRT  637
                DP L  + +A+KAT K G+GKLR+RP  G      G PW AL R TWRPDIRA ++S+
Sbjct  36180  DGDPLLVTLTNAIKATAKSGIGKLRQRPNMGVDHVFGDPWPALKRLTWRPDIRAMILSKA  36001

Query  638    RINLHRKIVKHAAFTGQYPIAILSDCVVYAAAGPSPLDFLPYRDGKPLPGGFKLGVNPGL  697
              R N+HRK+   A   G +P+A+ +DCVVYA    SPL  L   DG+P+ GGF+LGVNPG
Sbjct  36000  RTNMHRKMHHLATVAGCWPLAVNNDCVVYATDSLSPLPLLLGPDGQPIRGGFRLGVNPGS  35821

Query  698    VKHEGTQSVLWGEEVRERFDAPELNLARYIKDGTVT  733
              VKH+G+Q+V W  ++     A  +N+A  IKD ++
Sbjct  35820  VKHQGSQTVDWALDLL----ADGVNIANEIKDASLV  35725
```

Sequence of relevant vectors useful as genetic tools (in Genbank format) and graphical representation of relevant features.

For using this annotated sequences copy from “LOCUS” upto to “//” including both and paste in a new document, then save as “text only” or “plain text”. Rename the resulting file with a “.gb” extension.

pIJ13103

```
LOCUS           pIJ13103      8161 bp ds-DNA   circular     13-FEB-2018
DEFINITION  pBluescript II KS(+) with cas9-tracrRNA system from pCRISPomyces-2
ACCESSION
VERSION
KEYWORDS    artificial sequence; cloning vector; expression vector; vector.
SOURCE      synthetic construct
  ORGANISM  synthetic construct other sequences; artificial sequences.
COMMENT
COMMENT     Based on original sequence accession X52327.1
FEATURES             Location/Qualifiers
     source          complement(1..2264)
                     /organism="synthetic construct"
                     /mol_type="other DNA"
                     /db_xref="taxon:32630"
                     /label=source:synthetic construct
     misc_feature    complement(1..2264)
                     /note="phagemid pBluescriptII KS(+)"
                     /label=phagemid pBluescriptII KS(+)
     primer_bind     2172..2191
                     /label=T3
     primer_bind     complement(7546..7563)
                     /label=M13-fwd
     primer_bind     2134..2154
                     /label=M13-rev
     primer_bind     complement(7517..7537)
                     /label=T7
     rep_origin      1084..1766
                     /label=ColE1 origin
     rep_origin      7721..8027
                     /label=F1 ori
     CDS             7634..7702
                     /label=LacZ alpha
     misc_binding    2106..2128
                     /label=LacO
     CDS             327..986
                     /label=AmpR
     source          complement(7465..8161)
                     /organism="synthetic construct"
                     /mol_type="other DNA"
                     /db_xref="taxon:32630"
                     /label=source:synthetic construct(1)
     misc_feature    complement(7465..8161)
                     /note="phagemid pBluescriptII KS(+)"
                     /label=phagemid pBluescriptII KS(+)(1)
     source          2265..7464
                     /label=JP237-212_amplicon
     rep_origin      complement(2265..2270)
                     /vntifkey="33"
                     /label=ColE1
```

misc_feature complement(2271..2276)
/vntifkey="21"
/label=unique\XbaI\site
CDS complement(3349..7455)
/vntifkey="4"
/label=codon-optimized\SpCas9
terminator complement(3150..3179)
/vntifkey="43"
/label=fd-ter
promoter complement(2865..3149)
/vntifkey="30"
/label=gapdhp(EL)
misc_feature 2486..2835
/vntifkey="21"
/label=LacZ\cassette
terminator complement(2277..2326)
/vntifkey="43"
/label=oop\ter
primer_bind 2506..2531
/label=pIB_RP
primer_bind complement(2727..2753)
/label=pIB_FP
primer_bind complement(2699..2722)
/label=M13F_24mer
primer_bind 2543..2564
/label=M13R_22mer
primer_bind 2586..2605
/label=T3(1)
primer_bind complement(2679..2696)
/label=M13-fwd(1)
primer_bind 2548..2568
/label=M13-rev(1)
primer_bind complement(2650..2670)
/label=T7(1)
CDS 2767..2835
/label=LacZ alpha(1)
misc_binding 2520..2542
/label=LacO(1)
primer_bind complement(2930..2949)
/label=CRPMY2_F
primer_bind 7052..7071
/label=JP230
primer_bind 2265..2286
/label=JP212
primer_bind 7052..7071
/label=JP232
primer_bind complement(7439..7464)
/label=JP237_NdeI
misc_feature complement(2327..2407)
/vntifkey="21"
/label=gRNA-tracr
primer_bind 2341..2360
/label=CRPMY2_R
misc_feature complement(2410..2415)
/vntifkey="21"
/label=<\BbsI
misc_feature complement(2857..2862)
/vntifkey="21"
/label=BbsI\>

ORIGIN
1 gtggcacttt tcggggaaat gtgcgcggaa cccctatttg tttatttttc taaatacatt
61 caaatatgta tccgctcatg agacaataac cctgataaat gcttcaataa tattgaaaaa
121 ggaagagtat gagtattcaa catttccgtg tcgcccttat tccctttttt gcggcatttt
181 gccttcctgt ttttgctcac ccagaaacgc tggtgaaagt aaaagatgct gaagatcagt
241 tgggtgcacg agtgggttac atcgaactgg atctcaacag cggtaagatc cttgagagtt
301 ttcgccccga agaacgtttt ccaatgatga gcacttttaa agttctgcta tgtggcgcgg
361 tattatcccg tattgacgcc gggcaagagc aactcggtcg ccgcatacac tattctcaga
421 atgacttggt tgagtactca ccagtcacag aaaagcatct tacggatggc atgacagtaa
481 gagaattatg cagtgctgcc ataaccatga gtgataacac tgcggccaac ttacttctga
541 caacgatcgg aggaccgaag gagctaaccg cttttttgca caacatgggg gatcatgtaa
601 ctcgccttga tcgttgggaa ccggagctga atgaagccat accaaacgac gagcgtgaca
661 ccacgatgcc tgtagcaatg gcaacaacgt tgcgcaaact attaactggc gaactactta
721 ctctagcttc ccggcaacaa ttaatagact ggatggaggc ggataaagtt gcaggaccac
781 ttctgcgctc ggcccttccg gctggctggt ttattgctga taaatctgga gccggtgagc
841 gtgggtctcg cggtatcatt gcagcactgg ggccagatgg taagccctcc cgtatcgtag
901 ttatctacac gacggggagt caggcaacta tggatgaacg aaatagacag atcgctgaga
961 taggtgcctc actgattaag cattggtaac tgtcagacca agtttactca tatatacttt
1021 agattgattt aaaacttcat ttttaattta aaaggatcta ggtgaagatc ctttttgata
1081 atctcatgac caaaatccct taacgtgagt tttcgttcca ctgagcgtca gaccccgtag
1141 aaaagatcaa aggatcttct tgagatcctt tttttctgcg cgtaatctgc tgcttgcaaa
1201 caaaaaaacc accgctacca gcggtggttt gtttgccgga tcaagagcta ccaactcttt
1261 ttccgaaggt aactggcttc agcagagcgc agataccaaa tactgtcctt ctagtgtagc
1321 cgtagttagg ccaccacttc aagaactctg tagcaccgcc tacatacctc gctctgctaa
1381 tcctgttacc agtggctgct gccagtggcg ataagtcgtg tcttaccggg ttggactcaa
1441 gacgatagtt accggataag gcgcagcggt cgggctgaac ggggggttcg tgcacacagc
1501 ccagcttgga gcgaacgacc tacaccgaac tgagatacct acagcgtgag ctatgagaaa
1561 gcgccacgct tcccgaaggg agaaaggcgg acaggtatcc ggtaagcggc agggtcggaa
1621 caggagagcg cacgagggag cttccagggg gaaacgcctg gtatctttat agtcctgtcg
1681 ggtttcgcca cctctgactt gagcgtcgat ttttgtgatg ctcgtcaggg gggcggagcc
1741 tatggaaaaa cgccagcaac gcggcctttt tacggttcct ggccttttgc tggccttttg
1801 ctcacatgtt ctttcctgcg ttatcccctg attctgtgga taaccgtatt accgcctttg
1861 agtgagctga taccgctcgc cgcagccgaa cgaccgagcg cagcgagtca gtgagcgagg
1921 aagcggaaga gcgcccaata cgcaaaccgc ctctccccgc gcgttggccg attcattaat
1981 gcagctggca cgacaggttt cccgactgga aagcgggcag tgagcgcaac gcaattaatg
2041 tgagttagct cactcattag gcaccccagg ctttacactt tatgcttccg gctcgtatgt
2101 tgtgtggaat tgtgagcgga taacaatttc acacaggaaa cagctatgac catgattacg
2161 ccaagcgcgc aattaaccct cactaaaggg aacaaaagct gggtaccggg ccccccctcg
2221 aggtcgacgg tatcgataag cttgatatcg aattcctgca gcccctggcc tctagataaa
2281 aaacgcccgg cggcaaccga gcgttctgaa caaatccaga tggagtaaaa agcaccgact
2341 cggtgccact ttttcaagtt gataacggac tagccttatt ttaacttgct atttctagct
2401 ctaaaacatg tcttcaccgg tggaaagcgg gcagtgagcg caacgcaatt aatgtgagtt
2461 agctcactca ttaggcaccc caggctttac actttatgct tccggctcgt atgttgtgtg
2521 gaattgtgag cggataacaa tttcacacag gaaacagcta tgaccatgat tacgccaagc
2581 gcgcaattaa ccctcactaa agggaacaaa agctggagga cctcgagggg gggcccggta
2641 cccaattcgc cctatagtga gtcgtattac gcgcgctcac tggccgtcgt tttacaacgt
2701 cgtgactggg aaaaccctgg cgttacccaa cttaatcgcc ttgcagcaca tccccctttc
2761 gccagctggc gtaatagcga agaggcccgc accgatcgcc cttcccaaca gttgcgcagc
2821 ctgaatggcg aatgggacgc gccctgtagc ggctgagaag acttgcgtat cccctttcag
2881 atactcgcac taagaattgc aggaacgccc cgatcatagc ggtagccgcc cagatgctgc
2941 aagccttatc tggcagccgt ataaaaaaag caaccgaaca ggccattcac agaagtttca
3001 caccgctcgc cgaggggctc cgcacccggt gcgcgccgta cagcagcgcg cttctcccag
3061 catcggccag ttcgcccgat ccgtccgtgt cgcacagccg acggctgcgg taaggtgccc
3121 gtagacgcac gtccgaccga aggagcagca aaaaaaggct ccaaaaggag cctttaattg
3181 tatcggttta tcagcttgct ttcgaggtga atttcttaaa cagcttgata ccgatagttg
3241 cgccgacaat gacaacaacc atcgcccacg cataaccgat atattcggtc gctgaggctt
3301 gcagggagtc aaaggccgct tttgcgggga acgcgtagat ctgaattctc agtcgccgcc
3361 gagctgggag aggtcgatgc gggtttcgta caggccggtg atggactggt ggatcagggt
3421 cgcgtcgagg acctccttgg tgctggtgta ccgcttgcgg tcgatggtgg tgtcgaagta

3481 cttgaacgcg gcgggggcgc ccaggttggt gagggtgaac aggtggatga tgttctccgc
3541 ctgctcccgg atcggcttgt cgcggtgctt gttgtaggcg gacagcacct tgtcgaggtt
3601 cgcgtcggcc aggatgaccc gcttgctgaa ctccgagatc tgctcgatga tctcgtccag
3661 gtagtgcttg tgctgctcca cgaagagctg cttctgctcg ttgtcctcgg gcgagccctt
3721 cagcttctcg tagtgggacg cgaggtacag gaagttgacg tacttggacg ggagggccag
3781 ctcgttgccc ttctgcagct cgcccgcgct ggcgagcatc cgcttgcggc cgttctcgag
3841 ctcgaacagc gagtacttgg gcagcttgat gatgaggtcc ttcttgacct ccttgtagcc
3901 cttggcctcc aggaagtcga tcgggttctt ctcgaacgag gagcgctcca tgatggtgat
3961 gccgagcagc tccttcacgg acttcagctt cttgctcttg cccttctcga ccttcgccac
4021 gaccagcacg gagtaggcga cggtggggct gtcgaagccg ccgtacttct tcgggtccca
4081 gtccttcttc cgggcgatca gcttgtccga gttgcgcttg gggaggatgg actccttgct
4141 gaagccgccg gtctgcacct cggttttctt gacgatgttg acctgcggca tggacagcac
4201 cttccggacg gtggcgaagt cgcggccctt gtcccagacg atctcgccgg tttcgccgtt
4261 ggtttcgatc aggggccgct tgcggatctc gccgttggcc agggtgatct cggtcttgaa
4321 gaagttcatg atgttgctgt agaagaagta cttcgcggtg gccttgccga tctcctgctc
4381 cgacttggcg atcatcttgc gcacgtcgta gaccttgtag tcgccgtaga cgaactcgga
4441 ctccagcttc gggtacttct tgatcagcgc ggtgcccacg acggcgttca ggtacgcgtc
4501 gtgggcgtgg tggtagttgt tgatctcccg gaccttgtag aactggaagt ccttgcggaa
4561 gtcggacacc agcttgctct tgagggtgat caccttgacc tcgcggatca gcttgtcgtt
4621 ctcgtcgtac ttggtgttca tccggctgtc caggatctgg gccacgtgct tggtgatctg
4681 ccgggtttcg acgagctggc gcttgatgaa gcccgccttg tcgagctccg acaggccgcc
4741 ccgctcggcc ttggtcaggt tgtcgaactt gcgctgggtg atgagcttgg cgttgagcag
4801 ctggcgccag tagttcttca tcttcttcac gacctcctcc gagggcacgt tgtcggactt
4861 gccccggttc ttgtcggagc gggtcaggac cttgttgtcg atcgagtcgt ccttcaggaa
4921 ggactgcggc acgatgtggt cgacgtcgta gtcgctcagc cggttgatgt cgagctcctg
4981 gtccacgtac atgtcgcggc cgttctgcag gtagtagagg tacagcttct cgttctggag
5041 ctgggtgttc tcgaccgggt gctccttcag gatctgcgag ccgagctcct tgatgccctc
5101 ctcgatgcgc ttcatccgct cgcgggagtt cttctggccc ttctgggtgg tctggttctc
5161 ccgggccatc tcgatcacga tgttctcggg cttgtggcgg cccatcacct tgaccagctc
5221 gtccacgacc ttgacggtct ggaggatgcc cttcttgatc gccggggagc ccgccaggtt
5281 ggcgatgtgc tcgtggaggc tgtcgccctg gccggacacc tgggccttct ggatgtcctc
5341 cttgaaggtc agcgagtcgt cgtggatgag ctgcatgaag ttgcggttgg cgaagccgtc
5401 ggacttgagg aagtccagga tggtcttgcc gctctgcttg tcccggatgc cgttgatcag
5461 cttccgggag aggcggcccc agccggtgta ccggcgccgc ttcagctgct tcatcacctt
5521 gtcgtcgaac aggtgggcgt aggtcttgag ccgctcctcg atcatctcgc ggtcctcgaa
5581 gagggtcagg gtgaggacga tgtcctccag gatgtcctcg ttctcctcgt tgtcgaggaa
5641 gtccttgtcc ttgatgatct tgagcaggtc gtggtaggtg cccagggagg cgttgaagcg
5701 gtcctccacg cccgagatct cgacggagtc gaagcactcg atcttcttga agtagtcctc
5761 cttcagctgc ttcacggtga ccttgcggtt ggtcttgaag agcaggtcga cgatcgcctt
5821 cttctgctcg ccggacagga aggccggctt ccgcatgccc tcggtcacgt acttgacctt
5881 ggtcagctcg ttgtacacgg tgaagtactc gtagagcagg ctgtgcttgg gcaggacctt
5941 ctcgttcggg aggttcttgt cgaagttggt catgcgctcg atgaacgact gcgcggaggc
6001 gcccttgtcc acgacctcct cgaagttcca cggggtgatg gtttcctccg acttccgggt
6061 catccacgcg aaccgggagt tgccgcgggc cagggggccg acgtagtacg ggatccggaa
6121 ggtcaggatc ttctcgatct tctcgcggtt gtccttcagg aaggggtaga agtcctcctg
6181 gcgccggagg atggcgtgga gctcgcccag gtggatctgg tgcgggatgg agccgttgtc
6241 gaaggtccgc tgcttgcgga gcaggtcctc gcggttcagc ttgacgagca gctcctcggt
6301 gccgtccatc ttctccagga tcggcttgat gaacttgtag aactcctcct gcgacgcgcc
6361 gccgtcgatg tagccggcgt agccgttctt ggactggtcg aagaagatct ccttgtactt
6421 ctcgggcagc tgctggcgca cgagggcctt gagcagggtc aggtcctggt ggtgctcgtc
6481 gtaccgcttg atcatgctcg ccgacagcgg ggccttggtg atctcggtgt tgacccgcag
6541 gatgtcgctg agcaggatgg cgtccgagag gttcttcgcg gccaggaaga ggtccgcgta
6601 ctggtcgccg atctgggcga gcaggttgtc caggtcgtcg tcgtaggtgt ccttggacag
6661 ctggagcttc gcgtcctcgg ccaggtcgaa gttgctcttg aagttggggg tcaggccgag
6721 cgacagcgcg atcaggttgc cgaagaggcc gttcttcttc tcgcccggca gctgggcgat
6781 gaggttctcc aggcgccggg acttgctcag gcgcgcggag aggatcgcct tggcgtcgac
6841 gccgctggcg ttgatggggt tctcctcgaa cagctggttg taggtctgca ccagctggat
6901 gaagagcttg tcgacgtcgg agttgtccgg gttcaggtcg ccctcgatga ggaagtggcc
6961 gcggaacttg atcatgtgcg cgagggccag gtagatgagc cgcaggtccg ccttgtcggt

7021 cgagtcgacc agcttcttgc ggaggtggta gatggtgggg tacttctcgt ggtaggccac
7081 ctcgtcgacg atgttgccga agatcgggtg gcgctcgtgc ttcttgtcct cctccaccag
7141 gaagctctcc tcgagccggt ggaagaacga gtcgtcgacc ttggccatct cgttggagaa
7201 gatctcctgc aggtagcaga tgcggttctt gcgccgggtg tagcggcgac gggcggtccg
7261 cttcaggcgg gtcgcctcgg cggtttcgcc gctgtcgaag agcagggcgc cgatcaggtt
7321 cttcttgatc gagtgccggt cggtgttgcc caggaccttg aacttcttgg aggggacctt
7381 gtactcgtcg gtgatgaccg cccagcccac gctgttggtg ccgatgtcca ggccgatgct
7441 gtacttcttg tccatATGAC TAGTggggga tccactagtt ctagagcggc cgccaccgcg
7501 gtggagctcc aattcgccct atagtgagtc gtattacgcg cgctcactgg ccgtcgtttt
7561 acaacgtcgt gactgggaaa accctggcgt tacccaactt aatcgccttg cagcacatcc
7621 ccctttcgcc agctggcgta atagcgaaga ggcccgcacc gatcgccctt cccaacagtt
7681 gcgcagcctg aatggcgaat gggacgcgcc ctgtagcggc gcattaagcg cggcgggtgt
7741 ggtggttacg cgcagcgtga ccgctacact tgccagcgcc ctagcgcccg ctcctttcgc
7801 tttcttccct tcctttctcg ccacgttcgc cggctttccc cgtcaagctc taaatcgggg
7861 gctcccttta gggttccgat ttagtgcttt acggcacctc gaccccaaaa aacttgatta
7921 gggtgatggt tcacgtagtg ggccatcgcc ctgatagacg gtttttcgcc ctttgacgtt
7981 ggagtccacg ttctttaata gtggactctt gttccaaact ggaacaacac tcaaccctat
8041 ctcggtctat tcttttgatt tataagggat tttgccgatt tcggcctatt ggttaaaaaa
8101 tgagctgatt taacaaaaat ttaacgcgaa ttttaacaaa atattaacgc ttacaattta
8161 g
//

pIJ13104

```
LOCUS       pIJ13104    8370 bp ds-DNA   circular   16-AUG-2017
DEFINITION  pBluescript II KS(+) with cas9-tracrRNA system from pCRISPomyces-2
      and ermEp*+theophylline riboswitch from pGusT-ermEp1-E*
ACCESSION
VERSION
KEYWORDS   artificial sequence; cloning vector; expression vector; vector.
SOURCE    synthetic construct
  ORGANISM  synthetic construct other sequences; artificial sequences.
COMMENT
COMMENT    Based on original sequence accession X52327.1 and that of pIJ13103
FEATURES         Location/Qualifiers
   source        complement(1..2264)
            /organism="synthetic construct"
            /mol_type="other DNA"
            /db_xref="taxon:32630"
            /label=source:synthetic construct
   misc_feature   complement(1..2264)
            /note="phagemid pBluescriptII KS(+)"
            /label=phagemid pBluescriptII KS(+)
   primer_bind    2172..2191
            /label=T3
   primer_bind    complement(7755..7772)
            /label=M13-fwd
   primer_bind    2134..2154
            /label=M13-rev
   primer_bind    complement(7726..7746)
            /label=T7
   rep_origin     1084..1766
            /label=ColE1 origin
   rep_origin     7930..8236
            /label=F1 ori
   CDS          7843..7911
            /label=LacZ alpha
   misc_binding   2106..2128
            /label=LacO
   CDS          327..986
            /label=AmpR
   source        complement(7683..8370)
            /organism="synthetic construct"
            /mol_type="other DNA"
            /db_xref="taxon:32630"
            /label=source:synthetic construct(1)
   misc_feature   complement(7683..8370)
            /note="phagemid pBluescriptII KS(+)"
            /label=phagemid pBluescriptII KS(+)(1)
   source        2265..7455
            /label=JP237-212_amplicon
   rep_origin     complement(2265..2270)
            /vntifkey="33"
            /label=ColE1
   misc_feature   complement(2271..2276)
            /vntifkey="21"
            /label=unique\XbaI\site
   CDS          complement(3349..7455)
            /vntifkey="4"
            /label=codon-optimized\SpCas9
```

```
terminator      complement(3150..3179)
                /vntifkey="43"
                /label=fd-ter
promoter        complement(2865..3149)
                /vntifkey="30"
                /label=gapdhp(EL)
misc_feature    complement(7475..7512)
                /vntifkey="21"
                /label=Theophyllin-Aptamer
misc_feature    complement(2857..2862)
                /vntifkey="21"
                /label=BbsI\>
promoter        complement(7541..7581)
                /vntifkey="30"
                /label=ermE
misc_feature    complement(2410..2415)
                /vntifkey="21"
                /label=<\BbsI
misc_feature    2486..2835
                /vntifkey="21"
                /label=LacZ\cassette
misc_feature    complement(2327..2407)
                /vntifkey="21"
                /label=gRNA-tracr
terminator      complement(2277..2326)
                /vntifkey="43"
                /label=oop\ter
primer_bind     7456..7475
                /label=JP239
primer_bind     2506..2531
                /label=pIB_RP
primer_bind     complement(2727..2753)
                /label=pIB_FP
primer_bind     complement(2699..2722)
                /label=M13F_24mer
primer_bind     2543..2564
                /label=M13R_22mer
primer_bind     2586..2605
                /label=T3(1)
primer_bind     complement(2679..2696)
                /label=M13-fwd(1)
primer_bind     2548..2568
                /label=M13-rev(1)
primer_bind     complement(2650..2670)
                /label=T7(1)
CDS             2767..2835
                /label=LacZ alpha(1)
misc_binding    2520..2542
                /label=LacO(1)
primer_bind     complement(2930..2949)
                /label=CRPMY2_F
primer_bind     2341..2360
                /label=CRPMY2_R
primer_bind     7052..7071
                /label=JP230
primer_bind     2265..2286
                /label=JP212
primer_bind     7052..7071
                /label=JP232
```

primer_bind complement(7439..7455)
/label=JP237_NdeI
primer_bind 2129..2150
/label=M13-R_22mer
primer_bind 7775..7798
/label=M13F_24mer(1)
primer_bind 7052..7071
/label=JP230-232
ORIGIN
1 gtggcacttt tcggggaaat gtgcgcggaa cccctatttg tttatttttc taaatacatt
61 caaatatgta tccgctcatg agacaataac cctgataaat gcttcaataa tattgaaaaa
121 ggaagagtat gagtattcaa catttccgtg tcgcccttat tccctttttt gcggcatttt
181 gccttcctgt ttttgctcac ccagaaacgc tggtgaaagt aaaagatgct gaagatcagt
241 tgggtgcacg agtgggttac atcgaactgg atctcaacag cggtaagatc cttgagagtt
301 ttcgccccga agaacgtttt ccaatgatga gcacttttaa agttctgcta tgtggcgcgg
361 tattatcccg tattgacgcc gggcaagagc aactcggtcg ccgcatacac tattctcaga
421 atgacttggt tgagtactca ccagtcacag aaaagcatct tacggatggc atgacagtaa
481 gagaattatg cagtgctgcc ataaccatga gtgataacac tgcggccaac ttacttctga
541 caacgatcgg aggaccgaag gagctaaccg cttttttgca caacatgggg gatcatgtaa
601 ctcgccttga tcgttgggaa ccggagctga atgaagccat accaaacgac gagcgtgaca
661 ccacgatgcc tgtagcaatg gcaacaacgt tgcgcaaact attaactggc gaactactta
721 ctctagcttc ccggcaacaa ttaatagact ggatggaggc ggataaagtt gcaggaccac
781 ttctgcgctc ggcccttccg gctggctggt ttattgctga taaatctgga gccggtgagc
841 gtgggtctcg cggtatcatt gcagcactgg ggccagatgg taagccctcc cgtatcgtag
901 ttatctacac gacggggagt caggcaacta tggatgaacg aaatagacag atcgctgaga
961 taggtgcctc actgattaag cattggtaac tgtcagacca agtttactca tatatacttt
1021 agattgattt aaaacttcat ttttaattta aaaggatcta ggtgaagatc ctttttgata
1081 atctcatgac caaaatccct taacgtgagt tttcgttcca ctgagcgtca gaccccgtag
1141 aaaagatcaa aggatcttct tgagatcctt tttttctgcg cgtaatctgc tgcttgcaaa
1201 caaaaaaacc accgctacca gcggtggttt gtttgccgga tcaagagcta ccaactcttt
1261 ttccgaaggt aactggcttc agcagagcgc agataccaaa tactgtcctt ctagtgtagc
1321 cgtagttagg ccaccacttc aagaactctg tagcaccgcc tacatacctc gctctgctaa
1381 tcctgttacc agtggctgct gccagtggcg ataagtcgtg tcttaccggg ttggactcaa
1441 gacgatagtt accggataag gcgcagcggt cgggctgaac ggggggttcg tgcacacagc
1501 ccagcttgga gcgaacgacc tacaccgaac tgagatacct acagcgtgag ctatgagaaa
1561 gcgccacgct tcccgaaggg agaaaggcgg acaggtatcc ggtaagcggc agggtcggaa
1621 caggagagcg cacgagggag cttccagggg gaaacgcctg gtatctttat agtcctgtcg
1681 ggtttcgcca cctctgactt gagcgtcgat ttttgtgatg ctcgtcaggg gggcggagcc
1741 tatggaaaaa cgccagcaac gcggcctttt tacggttcct ggccttttgc tggccttttg
1801 ctcacatgtt ctttcctgcg ttatcccctg attctgtgga taaccgtatt accgcctttg
1861 agtgagctga taccgctcgc cgcagccgaa cgaccgagcg cagcgagtca gtgagcgagg
1921 aagcggaaga gcgcccaata cgcaaaccgc ctctccccgc gcgttggccg attcattaat
1981 gcagctggca cgacaggttt cccgactgga aagcgggcag tgagcgcaac gcaattaatg
2041 tgagttagct cactcattag gcaccccagg ctttacactt tatgcttccg gctcgtatgt
2101 tgtgtggaat tgtgagcgga taacaatttc acacaggaaa cagctatgac catgattacg
2161 ccaagcgcgc aattaaccct cactaaaggg aacaaaagct gggtaccggg ccccccctcg
2221 aggtcgacgg tatcgataag cttgatatcg aattcctgca gcccctggcc tctagataaa
2281 aaacgcccgg cggcaaccga gcgttctgaa caaatccaga tggagtaaaa agcaccgact
2341 cggtgccact ttttcaagtt gataacggac tagccttatt ttaacttgct atttctagct
2401 ctaaaacatg tcttcaccgg tggaaagcgg gcagtgagcg caacgcaatt aatgtgagtt
2461 agctcactca ttaggcaccc caggctttac actttatgct tccggctcgt atgttgtgtg
2521 gaattgtgag cggataacaa tttcacacag gaaacagcta tgaccatgat tacgccaagc
2581 gcgcaattaa ccctcactaa agggaacaaa agctggagga cctcgagggg gggcccggta
2641 cccaattcgc cctatagtga gtcgtattac gcgcgctcac tggccgtcgt tttacaacgt
2701 cgtgactggg aaaaccctgg cgttacccaa cttaatcgcc ttgcagcaca tccccctttc
2761 gccagctggc gtaatagcga agaggcccgc accgatcgcc cttcccaaca gttgcgcagc
2821 ctgaatggcg aatgggacgc gccctgtagc ggctgagaag acttgcgtat cccctttcag
2881 atactcgcac taagaattgc aggaacgccc cgatcatagc ggtagccgcc cagatgctgc
2941 aagccttatc tggcagccgt ataaaaaaag caaccgaaca ggccattcac agaagtttca

3001 caccgctcgc cgaggggctc cgcacccggt gcgcgccgta cagcagcgcg cttctcccag
3061 catcggccag ttcgcccgat ccgtccgtgt cgcacagccg acggctgcgg taaggtgccc
3121 gtagacgcac gtccgaccga aggagcagca aaaaaaggct ccaaaaggag cctttaattg
3181 tatcggttta tcagcttgct ttcgaggtga atttcttaaa cagcttgata ccgatagttg
3241 cgccgacaat gacaacaacc atcgcccacg cataaccgat atattcggtc gctgaggctt
3301 gcagggagtc aaaggccgct tttgcgggga acgcgtagat ctgaattctc agtcgccgcc
3361 gagctgggag aggtcgatgc gggtttcgta caggccggtg atggactggt ggatcagggt
3421 cgcgtcgagg acctccttgg tgctggtgta ccgcttgcgg tcgatggtgg tgtcgaagta
3481 cttgaacgcg gcgggggcgc ccaggttggt gagggtgaac aggtggatga tgttctccgc
3541 ctgctcccgg atcggcttgt cgcggtgctt gttgtaggcg gacagcacct tgtcgaggtt
3601 cgcgtcggcc aggatgaccc gcttgctgaa ctccgagatc tgctcgatga tctcgtccag
3661 gtagtgcttg tgctgctcca cgaagagctg cttctgctcg ttgtcctcgg gcgagccctt
3721 cagcttctcg tagtgggacg cgaggtacag gaagttgacg tacttggacg ggagggccag
3781 ctcgttgccc ttctgcagct cgcccgcgct ggcgagcatc cgcttgcggc cgttctcgag
3841 ctcgaacagc gagtacttgg gcagcttgat gatgaggtcc ttcttgacct ccttgtagcc
3901 cttggcctcc aggaagtcga tcgggttctt ctcgaacgag gagcgctcca tgatggtgat
3961 gccgagcagc tccttcacgg acttcagctt cttgctcttg cccttctcga ccttcgccac
4021 gaccagcacg gagtaggcga cggtggggct gtcgaagccg ccgtacttct tcgggtccca
4081 gtccttcttc cggcgatca gcttgtccga gttgcgcttg gggaggatgg actccttgct
4141 gaagccgccg gtctgcacct cggttttctt gacgatgttg acctgcggca tggacagcac
4201 cttccggacg gtggcgaagt cgcggccctt gtcccagacg atctcgccgg tttcgccgtt
4261 ggtttcgatc aggggccgct tgcggatctc gccgttggcc agggtgatct cggtcttgaa
4321 gaagttcatg atgttgctgt agaagaagta cttcgcggtg gccttgccga tctcctgctc
4381 cgacttggcg atcatcttgc gcacgtcgta gaccttgtag tcgccgtaga cgaactcgga
4441 ctccagcttc gggtacttct tgatcagcgc ggtgcccacg acggcgttca ggtacgcgtc
4501 gtgggcgtgg tggtagttgt tgatctcccg gaccttgtag aactggaagt ccttgcggaa
4561 gtcggacacc agcttgctct tgagggtgat caccttgacc tcgcggatca gcttgtcgtt
4621 ctcgtcgtac ttggtgttca tccggctgtc caggatctgg gccacgtgct tggtgatctg
4681 ccgggtttcg acgagctggc gcttgatgaa gcccgccttg tcgagctccg acaggccgcc
4741 ccgctcggcc ttggtcaggt tgtcgaactt gcgctgggtg atgagcttgg cgttgagcag
4801 ctggcgccag tagttcttca tcttcttcac gacctcctcc gagggcacgt tgtcggactt
4861 gccccggttc ttgtcggagc gggtcaggac cttgttgtcg atcgagtcgt ccttcaggaa
4921 ggactgcggc acgatgtggt cgacgtcgta gtcgctcagc cggttgatgt cgagctcctg
4981 gtccacgtac atgtcgcggc cgttctgcag gtagtagagg tacagcttct cgttctggag
5041 ctgggtgttc tcgaccgggt gctccttcag gatctgcgag ccgagctcct tgatgccctc
5101 ctcgatgcgc ttcatccgct cgcgggagtt cttctggccc ttctgggtgg tctggttctc
5161 ccgggccatc tcgatcacga tgttctcggg cttgtggcgg cccatcacct tgaccagctc
5221 gtccacgacc ttgacggtct ggaggatgcc cttcttgatc gccggggagc ccgccaggtt
5281 ggcgatgtgc tcgtggaggc tgtcgccctg gccggacacc tgggccttct ggatgtcctc
5341 cttgaaggtc agcgagtcgt cgtggatgag ctgcatgaag ttgcggttgg cgaagccgtc
5401 ggacttgagg aagtccagga tggtcttgcc gctctgcttg tcccggatgc cgttgatcag
5461 cttccgggag aggcggcccc agccggtgta ccggcgccgc ttcagctgct tcatcacctt
5521 gtcgtcgaac aggtgggcgt aggtcttgag ccgctcctcg atcatctcgc ggtcctcgaa
5581 gagggtcagg gtgaggacga tgtcctccag gatgtcctcg ttctcctcgt tgtcgaggaa
5641 gtccttgtcc ttgatgatct tgagcaggtc gtggtaggtg cccagggagg cgttgaagcg
5701 gtcctccacg cccgagatct cgacggagtc gaagcactcg atcttcttga agtagtcctc
5761 cttcagctgc ttcacggtga ccttgcggtt ggtcttgaag agcaggtcga cgatcgcctt
5821 cttctgctcg ccggacagga aggccggctt ccgcatgccc tcggtcacgt acttgacctt
5881 ggtcagctcg ttgtacacgg tgaagtactc gtagagcagg ctgtgcttgg gcaggacctt
5941 ctcgttcggg aggttcttgt cgaagttggt catgcgctcg atgaacgact gcgcggaggc
6001 gcccttgtcc acgacctcct cgaagttcca cggggtgatg gtttcctccg acttccgggt
6061 catccacgcg aaccgggagt tgccgcgggc caggggggccg acgtagtacg ggatccggaa
6121 ggtcaggatc ttctcgatct tctcgcggtt gtccttcagg aaggggtaga agtcctcctg
6181 gcgccggagg atggcgtgga gctcgcccag gtggatctgg tgcgggatgg agccgttgtc
6241 gaaggtccgc tgcttgcgga gcaggtcctc gcggttcagc ttgacgagca gctcctcggt
6301 gccgtccatc ttctccagga tcggcttgat gaacttgtag aactcctcct gcgacgcgcc
6361 gccgtcgatg tagccggcgt agccgttctt ggactggtcg aagaagatct ccttgtactt
6421 ctcgggcagc tgctggcgca cgagggcctt gagcagggtc aggtcctggt ggtgctcgtc
6481 gtaccgcttg atcatgctcg ccgacagcgg ggccttggtg atctcggtgt tgacccgcag

6541 gatgtcgctg agcaggatgg cgtccgagag gttcttcgcg gccaggaaga ggtccgcgta
6601 ctggtcgccg atctgggcga gcaggttgtc caggtcgtcg tcgtaggtgt ccttggacag
6661 ctggagcttc gcgtcctcgg ccaggtcgaa gttgctcttg aagttggggg tcaggccgag
6721 cgacagcgcg atcaggttgc cgaagaggcc gttcttcttc tcgcccggca gctgggcgat
6781 gaggttctcc aggcgccggg acttgctcag gcgcgcggag aggatcgcct tggcgtcgac
6841 gccgctggcg ttgatggggt tctcctcgaa cagctggttg taggtctgca ccagctggat
6901 gaagagcttg tcgacgtcgg agttgtccgg gttcaggtcg ccctcgatga ggaagtggcc
6961 gcggaacttg atcatgtgcg cgagggccag gtagatgagc cgcaggtccg ccttgtcggt
7021 cgagtcgacc agcttcttgc ggaggtggta gatggtgggg tacttctcgt ggtaggccac
7081 ctcgtcgacg atgttgccga agatcgggtg gcgctcgtgc ttcttgtcct cctccaccag
7141 gaagctctcc tcgagccggt ggaagaacga gtcgtcgacc ttggccatct cgttggagaa
7201 gatctcctgc aggtagcaga tgcggttctt gcgccgggtg tagcggcgac gggcggtccg
7261 cttcaggcgg gtcgcctcgg cggtttcgcc gctgtcgaag agcagggcgc cgatcaggtt
7321 cttcttgatc gagtgccggt cggtgttgcc caggaccttg aacttcttgg aggggacctt
7381 gtactcgtcg gtgatgaccg cccagcccac gctgttggtg ccgatgtcca ggccgatgct
7441 gtacttcttg tccatAtGgt tgcctcctta gcagggtgct gccaagggca tcaagacgat
7501 gctggtatca ccggaaccta tagtgagtcg tattggtacc cgctggatcc taccaaccgg
7561 cacgattgtc cagcccacaa cagatctcaa catccccgtt ttccctactt ctatttttaa
7621 gttgaagacc ctattaacac ttgacgatga cgaggtcgag aatcgcttta ctagcagtgg
7681 caactagttc tagagcggcc gccaccgcgg tggagctcca attcgcccta tagtgagtcg
7741 tattacgcgc gctcactggc cgtcgtttta caacgtcgtg actgggaaaa ccctggcgtt
7801 acccaactta atcgccttgc agcacatccc cctttcgcca gctggcgtaa tagcgaagag
7861 gcccgcaccg atcgcccttc ccaacagttg cgcagcctga atggcgaatg ggacgcgccc
7921 tgtagcggcg cattaagcgc ggcgggtgtg gtggttacgc gcagcgtgac cgctacactt
7981 gccagcgccc tagcgcccgc tcctttcgct ttcttccctt cctttctcgc cacgttcgcc
8041 ggctttcccc gtcaagctct aaatcggggg ctccctttag ggttccgatt tagtgcttta
8101 cggcacctcg accccaaaaa acttgattag ggtgatggtt cacgtagtgg gccatcgccc
8161 tgatagacgg tttttcgccc tttgacgttg gagtccacgt tctttaatag tggactcttg
8221 ttccaaactg gaacaacact caaccctatc tcggtctatt cttttgattt ataagggatt
8281 ttgccgattt cggcctattg gttaaaaaat gagctgattt aacaaaaatt taacgcgaat
8341 tttaacaaaa tattaacgct tacaatttag
//

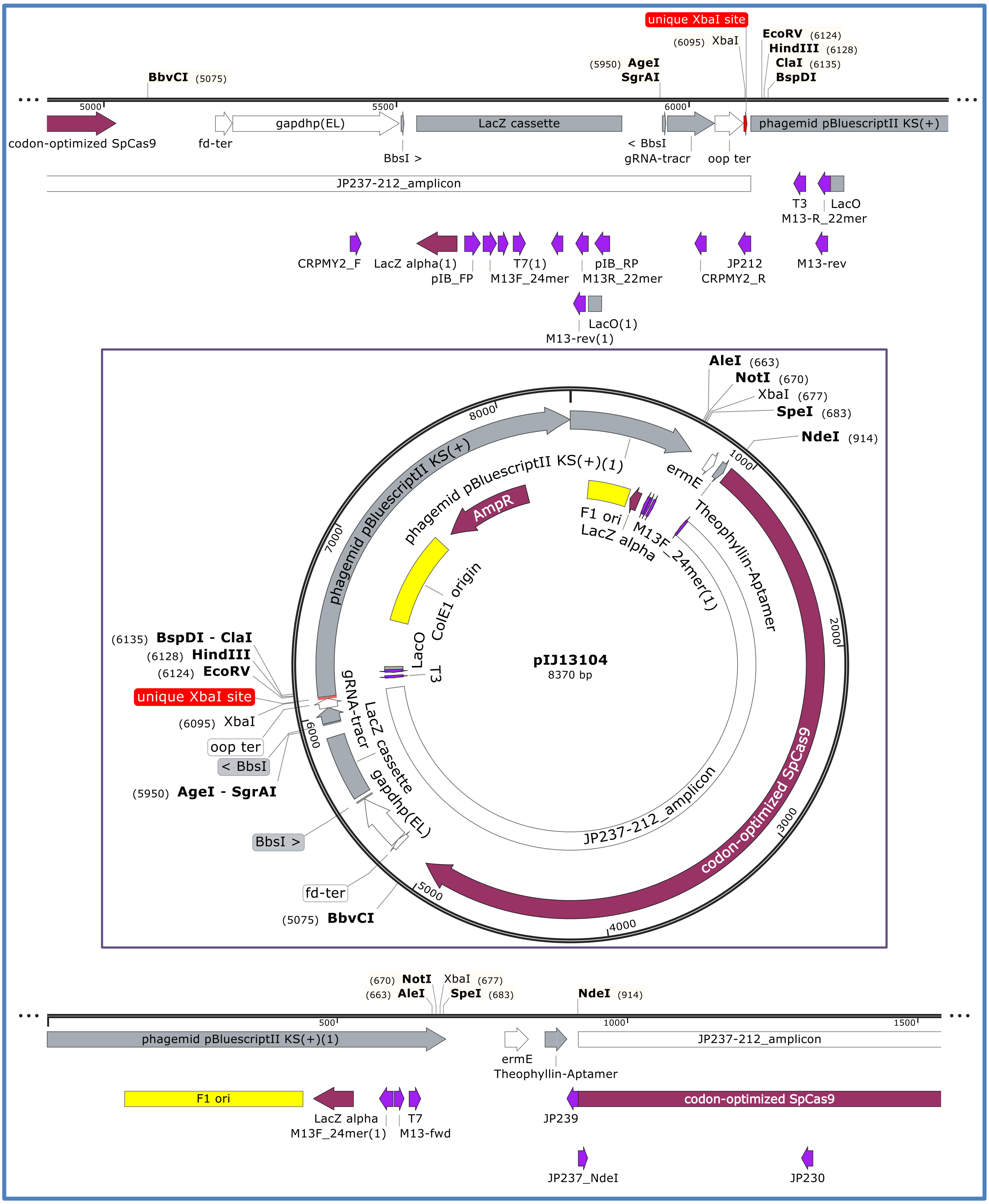
unique XbaI site
(6095) XbaI
EcoRV (6124)
HindIII (6128)
ClaI (6135)
BspDI
(5950) AgeI
SgrAI
BbvCI (5075)
5000
5500
6000
codon-optimized SpCas9
fd-ter
gapdhp(EL)
BbsI >
LacZ cassette
< BbsI
gRNA-tracr
oop ter
phagemid pBluescriptII KS(+)
JP237-212_amplicon
T3
LacO
M13-R_22mer
CRPMY2_F
LacZ alpha(1)
pIB_FP
T7(1)
M13F_24mer
pIB_RP
M13R_22mer
JP212
CRPMY2_R
M13-rev
LacO(1)
M13-rev(1)
AleI (663)
NotI (670)
XbaI (677)
SpeI (683)
NdeI (914)
8000
1000
7000
2000
3000
4000
5000
6000
phagemid pBluescriptII KS(+)
phagemid pBluescriptII KS(+)(1)
AmpR
ermE
F1 ori
LacZ alpha
M13F_24mer(1)
Theophyllin-Aptamer
ColE1 origin
LacO
T3
pIJ13104
8370 bp
(6135) BspDI - ClaI
(6128) HindIII
(6124) EcoRV
unique XbaI site
(6095) XbaI
oop ter
< BbsI
(5950) AgeI - SgrAI
gRNA-tracr
LacZ cassette
gapdhp(EL)
BbsI >
fd-ter
(5075) BbvCI
JP237-212_amplicon
codon-optimized SpCas9
(670) NotI
(663) AleI
XbaI (677)
SpeI (683)
NdeI (914)
500
1000
1500
phagemid pBluescriptII KS(+)(1)
ermE
Theophyllin-Aptamer
JP237-212_amplicon
F1 ori
LacZ alpha
M13F_24mer(1)
T7
M13-fwd
JP239
codon-optimized SpCas9
JP237_NdeI
JP230

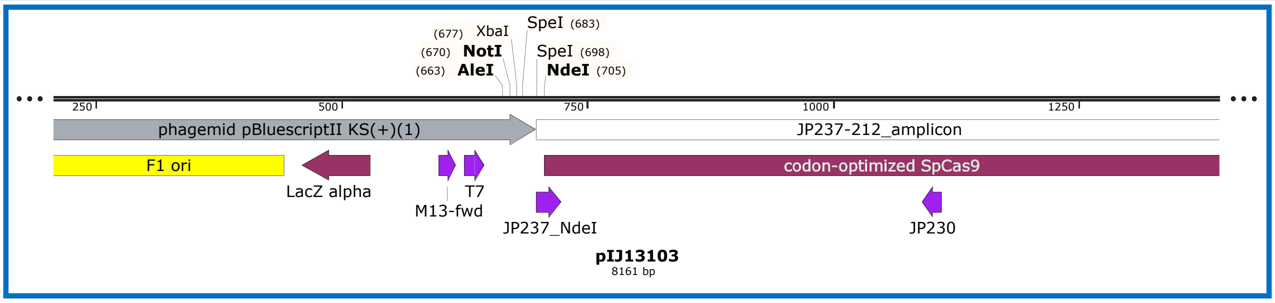
(677) XbaI
(670) NotI
(663) AleI
SpeI (683)
SpeI (698)
NdeI (705)
250
500
750
1000
1250
phagemid pBluescriptII KS(+)(1)
JP237-212_amplicon
F1 ori
LacZ alpha
T7
M13-fwd
codon-optimized SpCas9
JP237_NdeI
JP230
pIJ13103
8161 bp

REFERENCES

1. **Gomez-Escribano Juan Pablo, Liras Paloma, Pisabarro Agustín, Martín Juan F.** An rplKΔ29-PALG-32 mutation leads to reduced expression of the regulatory genes ccaR and claR and very low transcription of the ceaS2 gene for clavulanic acid biosynthesis in Streptomyces clavuligerus. *Molecular Microbiology* 2006;61:758–770, doi:10.1111/j.1365-2958.2006.05266.x.

2. **Gomez-Escribano JP, Martín JF, Hesketh A, Bibb MJ, Liras P**. Streptomyces clavuligerus relA-null mutants overproduce clavulanic acid and cephamycin C: negative regulation of secondary metabolism by (p)ppGpp. *Microbiology* 2008;154:744–755, doi:10.1099/mic.0.2007/011890-0.

3. **Sanchez L, Brana AF**. Cell density influences antibiotic biosynthesis in Streptomyces clavuligerus. *Microbiology* 1996;142:1209–1220, doi:10.1099/13500872-142-5-1209.

4. **Sambrook J, Fritsch EF, Maniatis T**. *Molecular cloning: a laboratory manual*. Cold Spring Harbor, N.Y.: Cold Spring Harbor Laboratory; 1989 ISBN 978-0-87969-309-1.

5. **Kieser T, Bibb MJ, Buttner MJ, Chater KF, Hopwood DA**. *Practical Streptomyces genetics*. Norwich: The John Innes Foundation; 2000 ISBN 978-0-7084-0623-6.

6. **Alting-Mees MA, Short JM**. pBluescript II: gene mapping vectors. *Nucleic Acids Res* 1989;17:9494–9494, doi:10.1093/nar/17.22.9494.

7. **Muth G**. The pSG5-based thermosensitive vector family for genome editing and gene expression in actinomycetes. *Appl Microbiol Biotechnol* 2018;102:9067–9080, doi:10.1007/s00253-018-9334-5.

8. **Cobb RE, Wang Y, Zhao H**. High-Efficiency Multiplex Genome Editing of Streptomyces Species Using an Engineered CRISPR/Cas System. *ACS Synth Biol* 2015;4:723–728, doi:10.1021/sb500351f.

9. **Tong Y, Charusanti P, Zhang L, Weber T, Lee SY**. CRISPR-Cas9 Based Engineering of Actinomycetal Genomes. *ACS Synth Biol* 2015;4:1020–1029, doi:10.1021/acssynbio.5b00038.

10. **Rudolph MM, Vockenhuber M-P, Suess B**. Synthetic riboswitches for the conditional control of gene expression in Streptomyces coelicolor. *Microbiology* 2013;159:1416–1422, doi:10.1099/mic.0.067322-0.

11. **Rudolph MM, Vockenhuber M-P, Suess B**. Chapter Fourteen - Conditional Control of Gene Expression by Synthetic Riboswitches in Streptomyces coelicolor. In: Burke-Aguero DH (editor). *Methods in Enzymology*. Academic Press. pp. 283–299.

12. **Wang Y, Cobb RE, Zhao H**. Chapter Twelve - High-Efficiency Genome Editing of Streptomyces Species by an Engineered CRISPR/Cas System. In: O'Connor SE (editor). *Methods in Enzymology*. Academic Press. pp. 271–284.

13. **Gomez-Escribano JP, Castro JF, Razmilic V, Chandra G, Andrews B, Asenjo JA, Bibb MJ**. The Streptomyces leeuwenhoekii genome: de novo sequencing and assembly in single contigs of the chromosome, circular plasmid pSLE1 and linear plasmid pSLE2. *BMC Genomics* 2015;16:485, doi:10.1186/s12864-015-1652-8.

14. **Rutherford K, Parkhill J, Crook J, Horsnell T, Rice P, Rajandream M-A, Barrell B**. Artemis: sequence visualization and annotation. *Bioinformatics* 2000;16:944–945, doi:10.1093/bioinformatics/16.10.944.

15. **Carver TJ, Rutherford KM, Berriman M, Rajandream M-A, Barrell BG, Parkhill J**. ACT: the Artemis comparison tool. *Bioinformatics* 2005;21:3422–3423, doi:10.1093/bioinformatics/bti553.

16. **Li H, Durbin R**. Fast and accurate short read alignment with Burrows-Wheeler transform. *Bioinformatics* 2009;25:1754–1760, doi:10.1093/bioinformatics/btp324.

17. **Li H, Durbin R**. Fast and accurate long-read alignment with Burrows–Wheeler transform. *Bioinformatics* 2010;26:589–595, doi:10.1093/bioinformatics/btp698.

18. **Li H, Handsaker B, Wysoker A, Fennell T, Ruan J, Homer N, Marth G, Abecasis G, Durbin R, 1000 Genome Project Data Processing Subgroup**. The Sequence Alignment/Map format and SAMtools. *Bioinformatics* 2009;25:2078–2079, doi:10.1093/bioinformatics/btp352.

19. **Carver T, Bohme U, Otto TD, Parkhill J, Berriman M**. BamView: viewing mapped read alignment data in the context of the reference sequence. *Bioinformatics* 2010;26:676–677, doi:10.1093/bioinformatics/btq010.

20. **García-Alcalde F, Okonechnikov K, Carbonell J, Cruz LM, Götz S, Tarazona S, Dopazo J, Meyer TF, Conesa A**. Qualimap: evaluating next-generation sequencing alignment data. *Bioinformatics* 2012;28:2678–2679, doi:10.1093/bioinformatics/bts503.

21. **Okonechnikov K, Conesa A, García-Alcalde F**. Qualimap 2: advanced multi-sample quality control for high-throughput sequencing data. *Bioinformatics* 2016;32:292–294, doi:10.1093/bioinformatics/btv566.

22. **Staden R, Beal KF, Bonfield JK**. The Staden Package, 1998. In: Misener S, Krawetz SA (editors). *Bioinformatics Methods and Protocols*. Totowa, NJ: Humana Press. pp. 115–130 ISBN 978-1-59259-192-3.

23. **Bonfield JK, Whitwham A**. Gap5—editing the billion fragment sequence assembly. *Bioinformatics* 2010;26:1699–1703, doi:10.1093/bioinformatics/btq268.

24. **Altschul SF, Madden TL, Schäffer AA, Zhang J, Zhang Z, Miller W, Lipman DJ**. Gapped BLAST and PSI-BLAST: a new generation of protein database search programs. *Nucleic Acids Res* 1997;25:3389–3402, doi:10.1093/nar/25.17.3389.

25. **Santiago-Sotelo P, Ramirez-Prado JH**. prfectBLAST: a platform-independent portable front end for the command terminal BLAST+ stand-alone suite. *BioTechniques* 2012;53:299–300, doi:10.2144/000113953.

26. **Aziz RK, Bartels D, Best AA, DeJongh M, Disz T, Edwards RA, Formsma K, Gerdes S, Glass EM, Kubal M, Meyer F, Olsen GJ, Olson R, Osterman AL, Overbeek RA, McNeil LK, Paarmann D, Paczian T, Parrello B, Pusch GD, Reich C, Stevens R, Vassieva O, Vonstein V, Wilke A, Zagnitko O**. The RAST Server: Rapid Annotations using Subsystems Technology. *BMC Genomics* 2008;9:75, doi:10.1186/1471-2164-9-75.

27. **Overbeek R, Olson R, Pusch GD, Olsen GJ, Davis JJ, Disz T, Edwards RA, Gerdes S, Parrello B, Shukla M, Vonstein V, Wattam AR, Xia F, Stevens R**. The SEED and the Rapid Annotation of microbial genomes using Subsystems Technology (RAST). *Nucl Acids Res* 2014;42:D206–D214, doi:10.1093/nar/gkt1226.

28. **Blin K, Wolf T, Chevrette MG, Lu X, Schwalen CJ, Kautsar SA, Suarez Duran HG, de Los Santos ELC, Kim HU, Nave M, Dickschat JS, Mitchell DA, Shelest E, Breitling R, Takano E, Lee SY, Weber T, Medema MH**. antiSMASH 4.0-improvements in chemistry prediction and gene cluster boundary identification. *Nucleic Acids Res* 2017;45:W36–W41, doi:10.1093/nar/gkx319.

29. **Simão FA, Waterhouse RM, Ioannidis P, Kriventseva EV, Zdobnov EM**. BUSCO: assessing genome assembly and annotation completeness with single-copy orthologs. *Bioinformatics* 2015;31:3210–3212, doi:10.1093/bioinformatics/btv351.

30. **Waterhouse RM, Tegenfeldt F, Li J, Zdobnov EM, Kriventseva EV**. OrthoDB: a hierarchical catalog of animal, fungal and bacterial orthologs. *Nucleic Acids Research* 2013;41:D358–D365, doi:10.1093/nar/gks1116.

31. **Medema MH, Trefzer A, Kovalchuk A, Berg M van den, Müller U, Heijne W, Wu L, Alam MT, Ronning CM, Nierman WC, Bovenberg RAL, Breitling R, Takano E**. The Sequence of a 1.8-Mb Bacterial Linear Plasmid Reveals a Rich Evolutionary Reservoir of Secondary Metabolic Pathways. *Genome Biol Evol* 2010;2:212–224, doi:10.1093/gbe/evq013.

32. **Yang C-C, Sun W-C, Wang W-Y, Huang C-H, Lu F-S, Tseng S-M, Chen CW**. Mutational Analysis of the Terminal Protein Tpg of Streptomyces Chromosomes: Identification of the Deoxynucleotidylation Site. *PLOS ONE* 2013;8:e56322, doi:10.1371/journal.pone.0056322.
